# Supplementary material for: From stem cells to somites: Revealing genetic and exogenous factors of human embryogenesis
Source: Stem Cell Reports. 2026 Aug 6;21(9):103023. doi: 10.1016/j.stemcr.2026.103023 (PMC13555414; doi:10.1016/j.stemcr.2026.103023)
Supplement: Supplementary file 1 [file mmc1.pdf]

Stem Cell Reports, Volume *21*

Supplemental Information

From stem cells to somites: Revealing genetic and exogenous factors of human embryogenesis

Maria Teresa Alejo-Vinogradova, Yunna E. Strøm, Lieke A. Lamers, Yolanda W. Chang, Martijn J. Hendriksen, Mariya Moosajee, Michiel Vermeulen, and Suzan Stelloo

Supplemental figures and legends

Figure S1

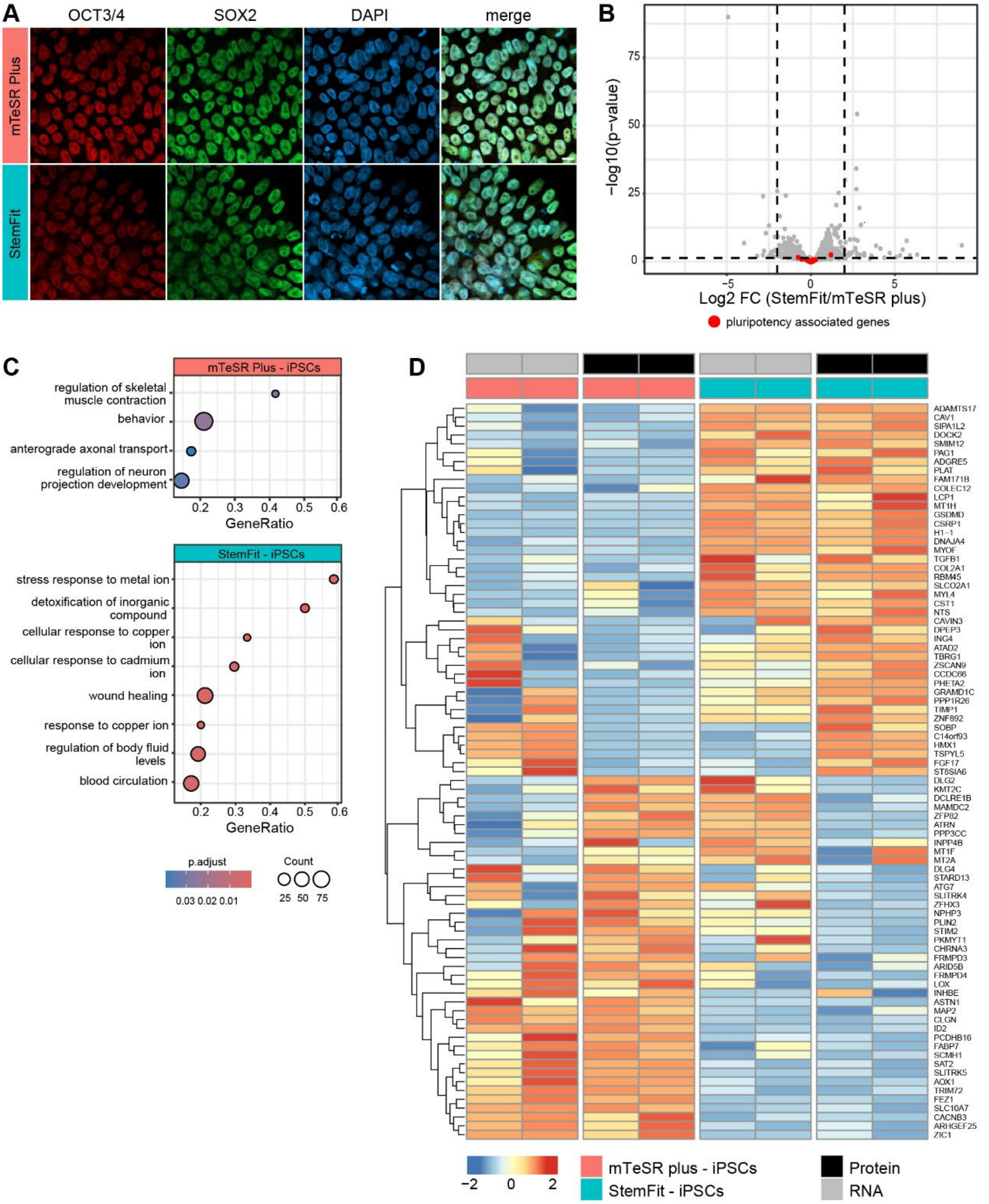

Figure S1. Comparing iPSCs cultured in mTeSR and StemFit media, related to Figure 1.
(A) Immunofluorescence staining of OCT3/4 and SOX2 with DAPI in undifferentiated wild-type EBL.cl3 iPSCs cultured in mTeSR Plus or StemFit medium. Scale bar = 10 µm.
(B) Volcano plot showing differential gene expression between iPSCs cultured in StemFit media versus iPSCs cultured in mTeSR Plus. Red dots represent 28 genes associated with the transcriptional regulation of pluripotent stem cells, based on the Reactome pathway database.

(C) GSEA performed on a ranked list of all genes comparing iPSCs cultured in StemFit versus mTeSR Plus medium. The plot displays the top activated (enriched in StemFit cultured iPSCs) and suppressed (enriched in mTeSR Plus cultured iPSCs) GO Biological Process terms.
(D) Heatmap displaying scaled RNA expression and corresponding protein expression. Only genes or proteins identified as differentially expressed in either modality (RNA-seq or proteomics) with a $\log_2$ FC > 2 and meeting significance thresholds are included.

Figure S2

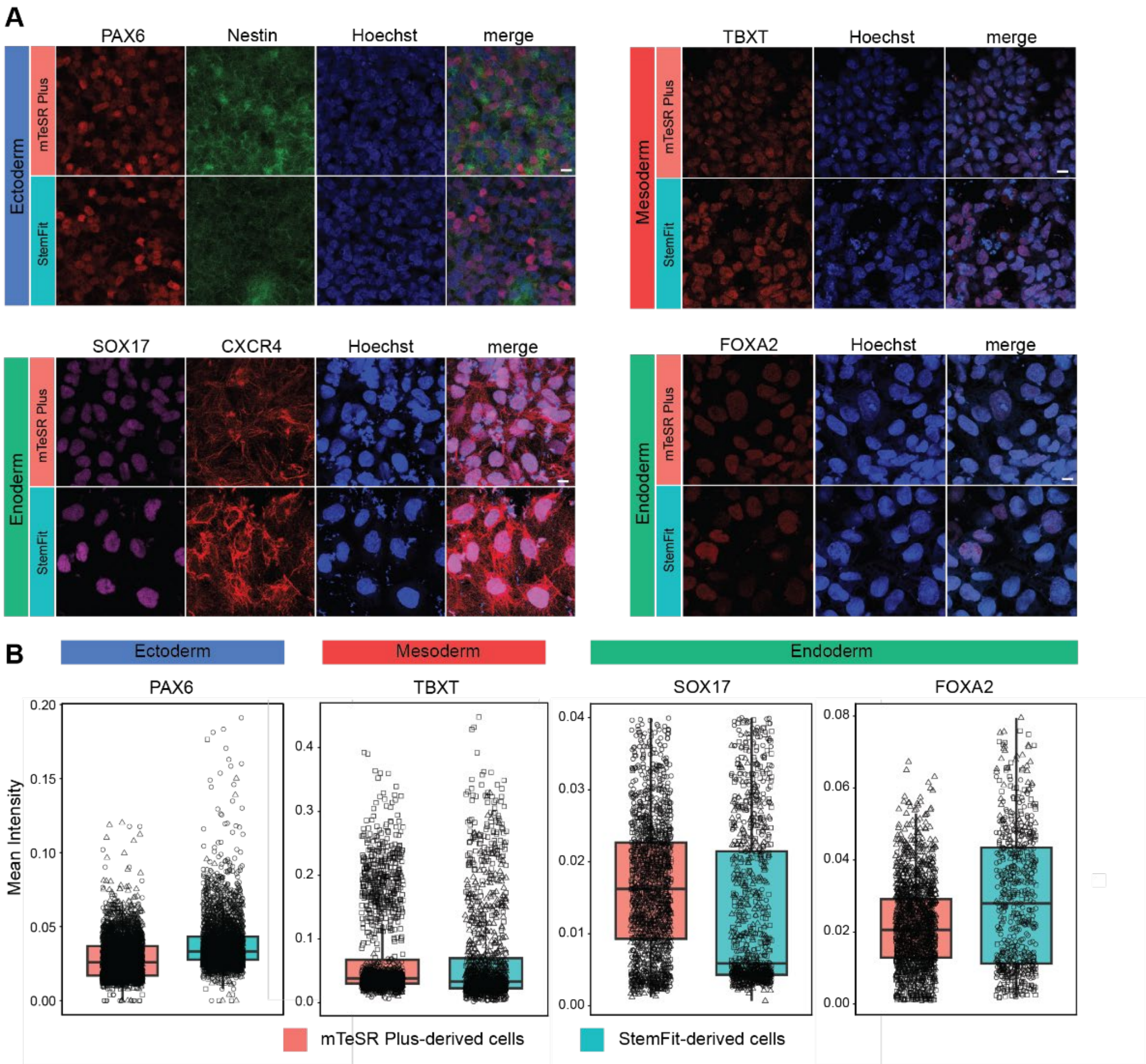

Figure S2. Trilineage differentiation potential of iPSCs maintained in mTeSR plus or StemFit medium, related to Figure 1.

(A) Wild-type EBL.cl3 iPSCs cultured in mTeSR Plus or StemFit medium were subjected to directed differentiation toward ectoderm, mesoderm, and endoderm lineages. Representative immunofluorescence images show expression of lineage-specific markers: ectoderm (PAX6/Nestin), mesoderm (Brachyury/TBXT), and endoderm (SOX17/FOXA2/CXCR4). Nuclei were stained with Hoechst. Scale bar = 10 µm.

(B) Quantification of marker-positive cells is shown as mean fluorescence intensity with each dot representing a single nucleus and different symbols denoting independent experiments.

Figure S3

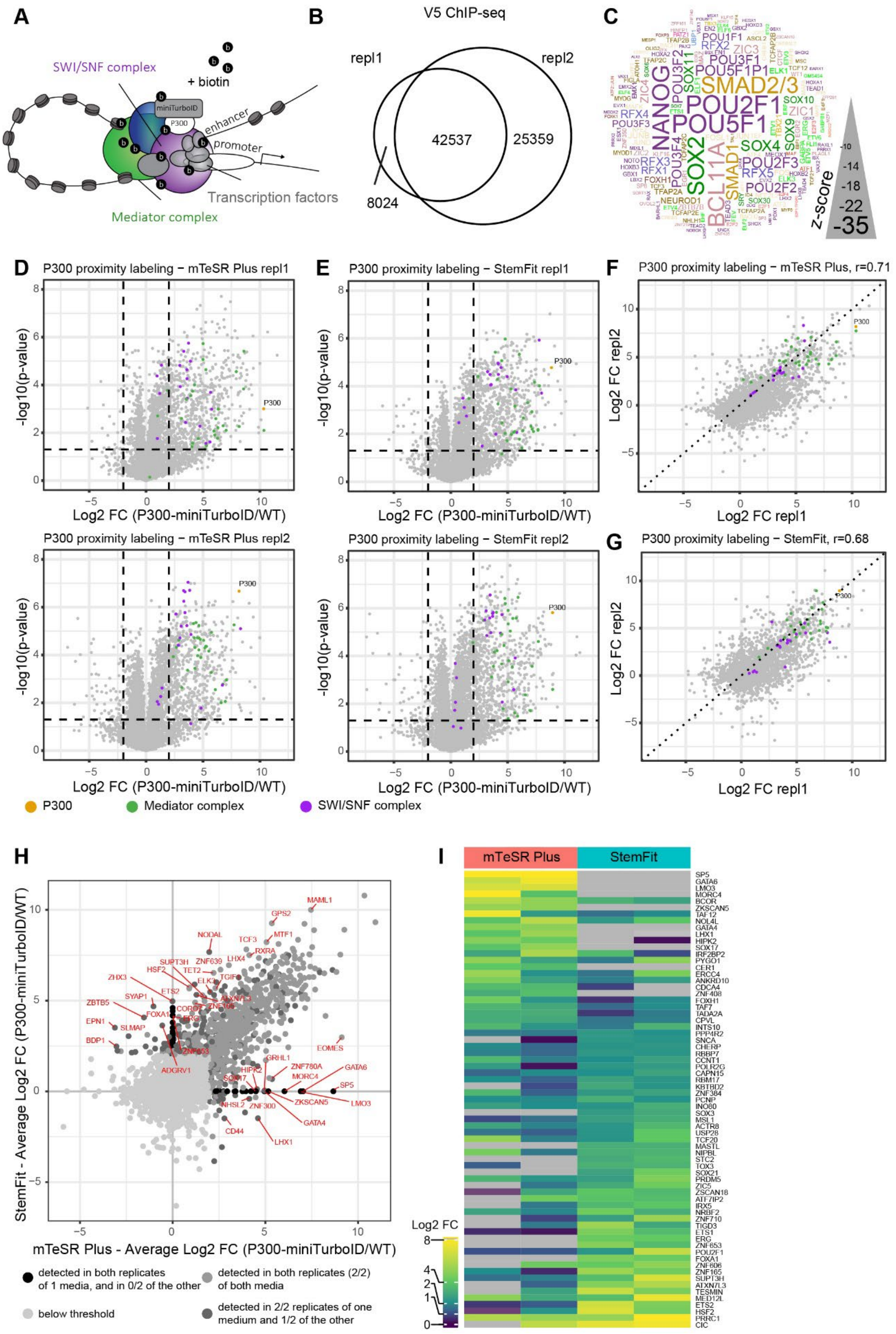
A
SWI/SNF complex
+ biotin
miniTurboID
P300
enhancer
promoter
Transcription factors
Mediator complex
B
V5 ChIP-seq
repl1
repl2
42537
25359
8024
C
NANOG
SOX2
POU5F1
POU2F1
SMAD2/3
BCL11A
z-score
-10
-14
-18
-22
-35
D
P300 proximity labeling − mTeSR Plus repl1
P300 proximity labeling − mTeSR Plus repl2
E
P300 proximity labeling − StemFit repl1
P300 proximity labeling − StemFit repl2
-log10(p-value)
Log2 FC (P300-miniTurboID/WT)
F
P300 proximity labeling − mTeSR Plus, r=0.71
G
P300 proximity labeling − StemFit, r=0.68
Log2 FC repl1
Log2 FC repl2
P300
Mediator complex
SWI/SNF complex
H
StemFit - Average Log2 FC (P300-miniTurboID/WT)
mTeSR Plus - Average Log2 FC (P300-miniTurboID/WT)
detected in both replicates of 1 media, and in 0/2 of the other
detected in both replicates (2/2) of both media
below threshold
detected in 2/2 replicates of one medium and 1/2 of the other
I
mTeSR Plus
StemFit
Log2 FC

Figure S3. P300 proximity labeling reveals culture medium specific transcription factors, related to Figure 1.

(A) Schematic of P300 proximity labeling. Upon biotin stimulation, all proteins in proximity to the fusion protein P300-miniTurboID become biotinylated, including P300 itself through self-biotinylation.

(B) Venn diagram illustrating the overlap of P300 binding sites between replicate 1 and replicate 2.

(C) Transcription factor motif enrichment at P300 binding sites. Font size represents the *Z* score (the smaller the *Z* score, the more enriched, the larger the font size), and colors represent transcription factor families.

(D) Volcano plot of streptavidin affinity purifications of biotin-treated P300-miniTurboID-expressing EBL.cl3 cells compared to biotin-treated wild-type EBL.cl3 iPSCs cultured in mTeSR Plus. Each plot corresponds to an individual biological replicate (with three technical replicates). Horizontal dashed lines represent a *p* value threshold of 0.05 and vertical dashed lines mark a $\log_2$ FC threshold of 2. Members of the SWI/SNF chromatin remodeling complex are highlighted in purple and components of the members of the Mediator complex are shown in green.

(E) Volcano plot of streptavidin affinity purifications of biotin-treated P300-miniTurboID-expressing EBL.cl3 cells compared to biotin-treated wild-type EBL.cl3 iPSCs cultured in StemFit. Each plot corresponds to an individual biological replicate (with three technical replicates). Horizontal dashed lines represent a *p* value threshold of 0.05 and vertical dashed lines mark a $\log_2$ FC threshold of 2. Members of the SWI/SNF chromatin remodeling complex are highlighted in purple and components of the members of the Mediator complex are shown in green.

(F) Correlation plot showing the $\log_2$ FC in protein enrichment from streptavidin affinity purifications of biotin-treated P300-miniTurboID-expressing cells compared to biotin-treated wild-type cells, cultured in mTeSR Plus medium.

(G) Correlation plot showing the $\log_2$ FC in protein enrichment from streptavidin affinity purifications of biotin-treated P300-miniTurboID-expressing cells compared to biotin-treated wild-type cells, cultured in StemFit medium.

(H) Scatter plot showing the mean $\log_2$ FC for proteins enriched in streptavidin affinity purifications of biotin-treated P300-miniTurboID-expressing cells compared to biotin-treated wild-type cells, comparing two different media conditions. $\log_2$ FC in each condition is calculated relative to a biotin-treated wild-type control and averaged across two independent replicates. Only proteins identified in both replicates are included, or retained if identified in both replicates of the other condition. The x-axis indicates the average $\log_2$ FC of iPSCs cultured in mTeSR Plus and the y-axis indicates the average $\log_2$ FC of iPSCs cultured in StemFit. Proteins enriched in either medium are labeled in red.

(I) Heatmap displaying $\log_2$ FC values for proteins enriched in streptavidin affinity purifications of biotin-treated P300-miniTurboID-expressing cells compared to biotin-treated wild-type cells. Proteins included have a $\log_2$ FC > 2 and *p* value <0.05 in both replicates of at least one condition but a $\log_2$ FC <1 in the other condition.

Figure S4

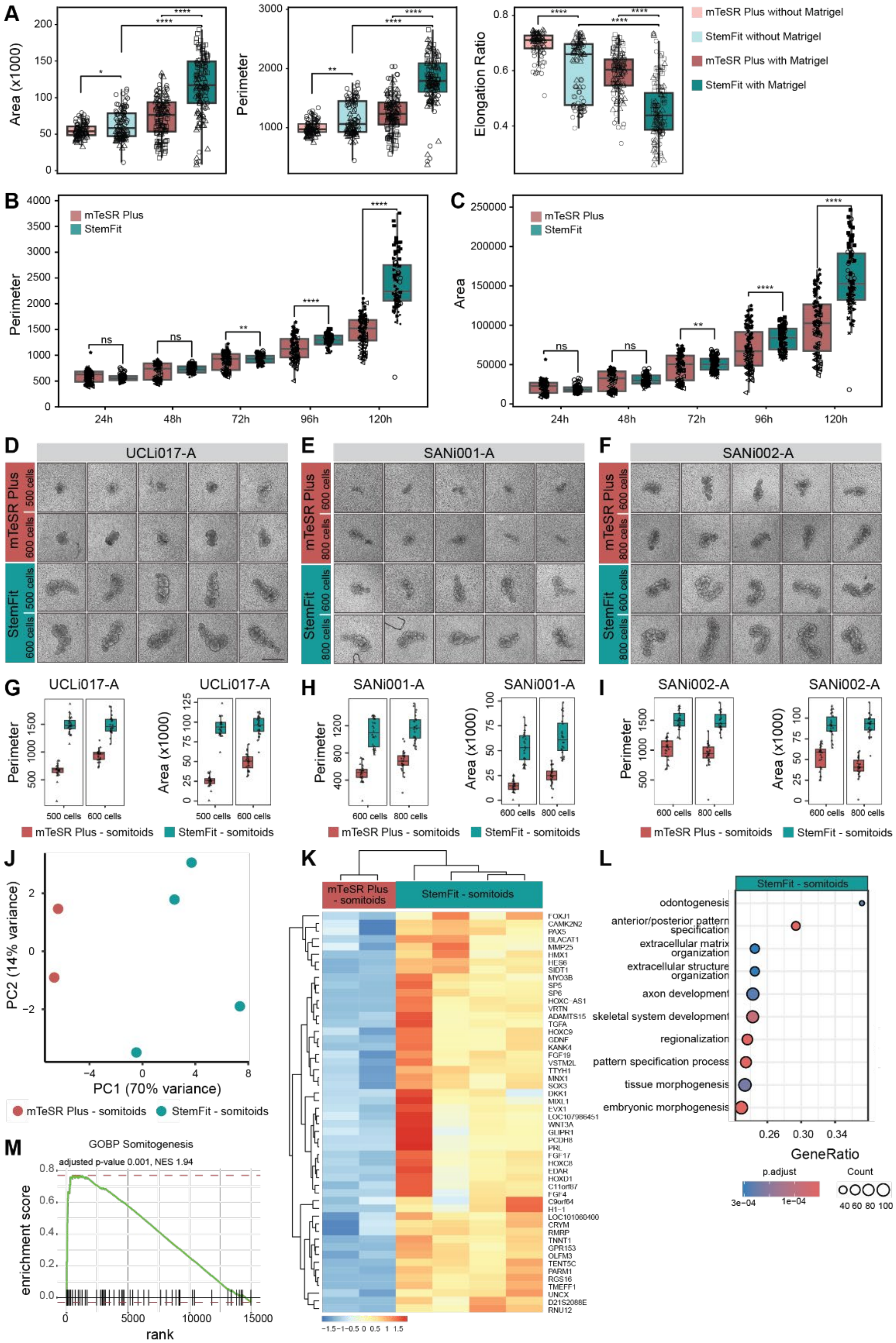

A
Area (x1000)
Perimeter
Elongation Ratio
mTeSR Plus without Matrigel
StemFit without Matrigel
mTeSR Plus with Matrigel
StemFit with Matrigel
B
Perimeter
mTeSR Plus
StemFit
24h
48h
72h
96h
120h
C
Area
D
UCLi017-A
E
SANi001-A
F
SANi002-A
mTeSR Plus
StemFit
500 cells
600 cells
800 cells
G
H
I
mTeSR Plus - somitoids
StemFit - somitoids
J
PC1 (70% variance)
PC2 (14% variance)
K
mTeSR Plus - somitoids
StemFit - somitoids
FOXJ1
CAMK2N2
PAX5
BLACAT1
MMP25
HMX1
HES6
SIDT1
MYO3B
SP5
SP6
HOXC-AS1
VRTN
ADAMTS15
TGFA
HOXC9
GDNF
KANK4
FGF19
VSTM2L
TTYH1
MNX1
SOX3
DKK1
MIXL1
EVX1
LOC107986451
WNT3A
GLIPR1
PCDH8
PRL
FGF17
HOXC8
EDAR
HOXD1
C11orf87
FGF4
C9orf64
H1-1
LOC101060400
CRYM
RMRP
TNNT1
GPR153
OLFM3
TENT5C
PARM1
RGS16
TMEFF1
UNCX
D21S2088E
RNU12
L
StemFit - somitoids
odontogenesis
anterior/posterior pattern specification
extracellular matrix organization
extracellular structure organization
axon development
skeletal system development
regionalization
pattern specification process
tissue morphogenesis
embryonic morphogenesis
GeneRatio
p.adjust
Count
M
GOBP Somitogenesis
adjusted p-value 0.001, NES 1.94
enrichment score
rank

Figure S4. Impact of maintenance medium on somitoid formation efficiency, related to Figure 2.
(A) Quantification of the area, perimeter and elongation ratio of somitoids generated from wild-type EBL.cl3 iPSCs pre-cultured in either mTeSR Plus or StemFit medium with or without Matrigel during the final 24 hours. Each dot represents a single somitoid. Different shapes indicate distinct biological replicates. Reported *p* values are determined with Mann-Whitney-Wilcoxon test two-sided with Bonferroni correction.
(B) Quantification of the perimeter of somitoids across time points generated from wild-type EBL.cl3 iPSCs pre-cultured in either mTeSR Plus or StemFit medium. Somitoids were embedded in Matrigel for the final 24h (120h_mg). Each dot represents a single somitoid. Different shapes indicate distinct biological replicates. Reported *p* values are determined with Mann-Whitney-Wilcoxon test two-sided with Bonferroni correction.
(C) Quantification of the area of somitoids across time points generated from wild-type EBL.cl3 iPSCs pre-cultured in either mTeSR Plus or StemFit medium. Somitoids were embedded in Matrigel for the final 24h (120h_mg). Each dot represents a single somitoid. Different shapes indicate distinct biological replicates. Reported *p* values are determined with Mann-Whitney-Wilcoxon test two-sided with Bonferroni correction.
(D) Representative images of day 5 Matrigel-embedded somitoids derived from UCLi017-A iPSCs pre-cultured in mTeSR Plus or StemFit medium. Differentiation was started with the indicated amount of cells. Scale bar = 200 µm.
(E) Representative images of day 5 somitoids derived from SANi001-A iPSCs pre-cultured in mTeSR Plus or StemFit medium. Differentiation was started with the indicated amount of cells. Scale bar = 200 µm.
(F) Representative images of day 5 somitoids derived from SANi002-A iPSCs pre-cultured in mTeSR Plus or StemFit medium. Differentiation was started with the indicated amount of cells. Scale bar = 200 µm.
(G) Quantification of the perimeter and area of somitoids generated from UCLi017-A iPSCs pre-cultured in either mTeSR Plus or StemFit medium. Each dot represents a single somitoid at day 5.
(H) Quantification of the perimeter and area of somitoids generated from SANi001-A iPSCs pre-cultured in either mTeSR Plus or StemFit medium. Each dot represents a single somitoid at day 5.
(I) Quantification of the perimeter and area of somitoids generated from SANi002-A iPSCs pre-cultured in either mTeSR Plus or StemFit medium. Each dot represents a single somitoid at day 5.
(J) PCA based on the top 500 most variable expressed genes in somitoids derived from iPSCs pre-cultured in either mTeSR Plus or StemFit medium. Each dot represents an independent biological replicate.
(K) Heatmap showing scaled expression of 52 differentially expressed genes (p value <0.05 and log2 FC >2) between somitoids derived from iPSCs maintained in different pre-differentiation media (mTeSR Plus vs. StemFit).
(L) GSEA performed on a ranked list of all genes comparing somitoids derived from iPSCs cultured in StemFit versus mTeSR Plus medium. The plot displays the top activated (enriched in StemFit cultured iPSCs) and suppressed (enriched in mTeSR Plus cultured iPSCs) GO Biological Process terms.
(M) Enrichment plot for the gene set “GOBP Somitogenesis” from the MSigDB database. The vertical black lines indicate the positions of genes from the set (67 genes) within the ranked list. Genes are ranked from most upregulated in somitoids derived from StemFit cultured iPSCs compared to somitoids derived from mTeSR Plus cultured iPSCs on the left to most downregulated on the right.

Figure S5

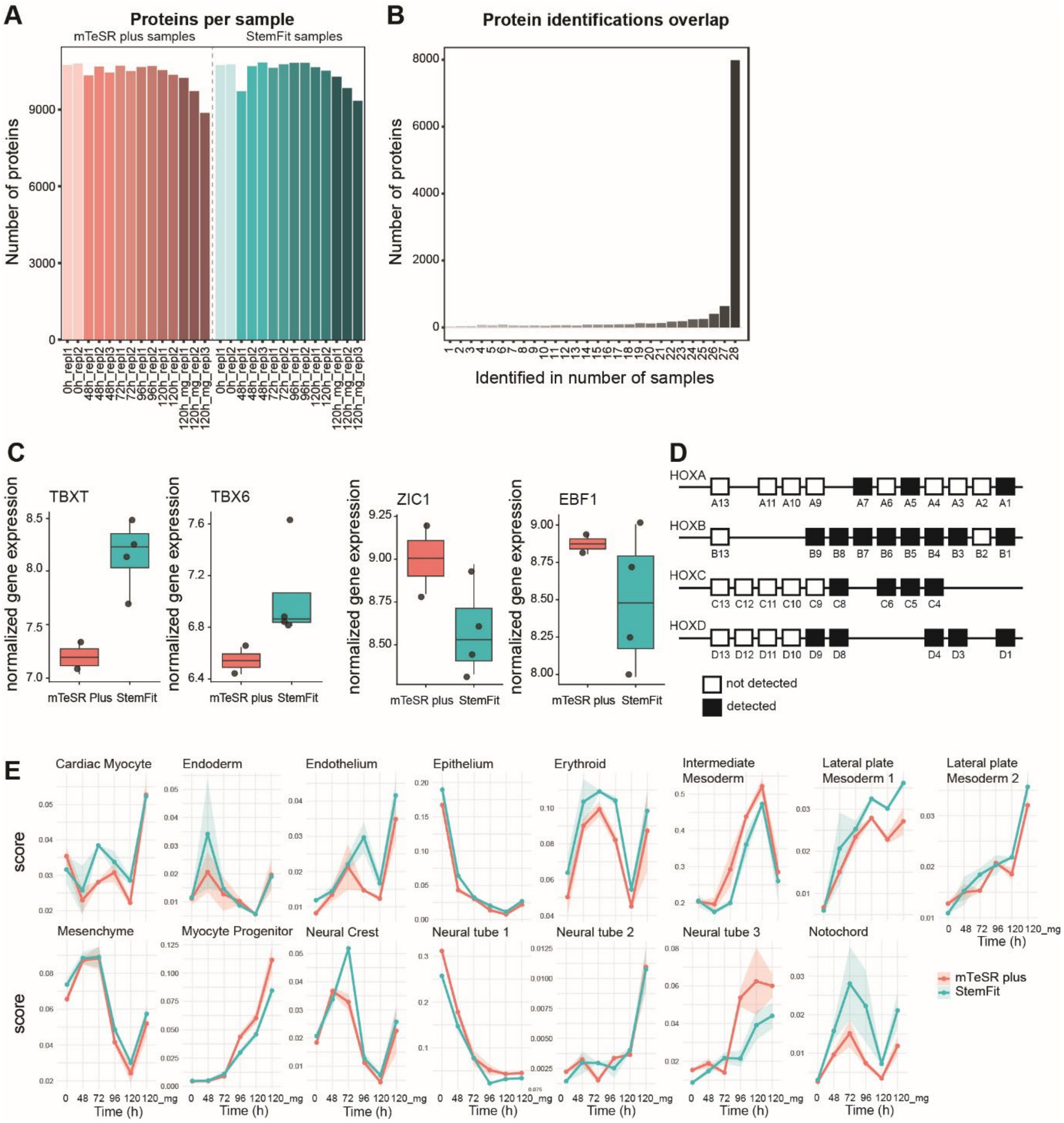

Figure S5. Temporal proteomic analysis of somitoid differentiation, related to Figure 3.
(A) Number of proteins retained after removal of contaminants and retaining proteins identified with at least two peptides.
(B) Distribution of protein overlap across the 28 samples.
(C) RNA expression levels corresponding to four selected differentially expressed proteins (DEPs).
(D) HOX protein detection across clusters HOXA–D. Black boxes indicate detection.
(E) Scaden-based deconvolution of proteomics data showing relative cell type contributions. Values represent relative abundances and are intended for comparison across time points rather than absolute quantification.

Figure S6

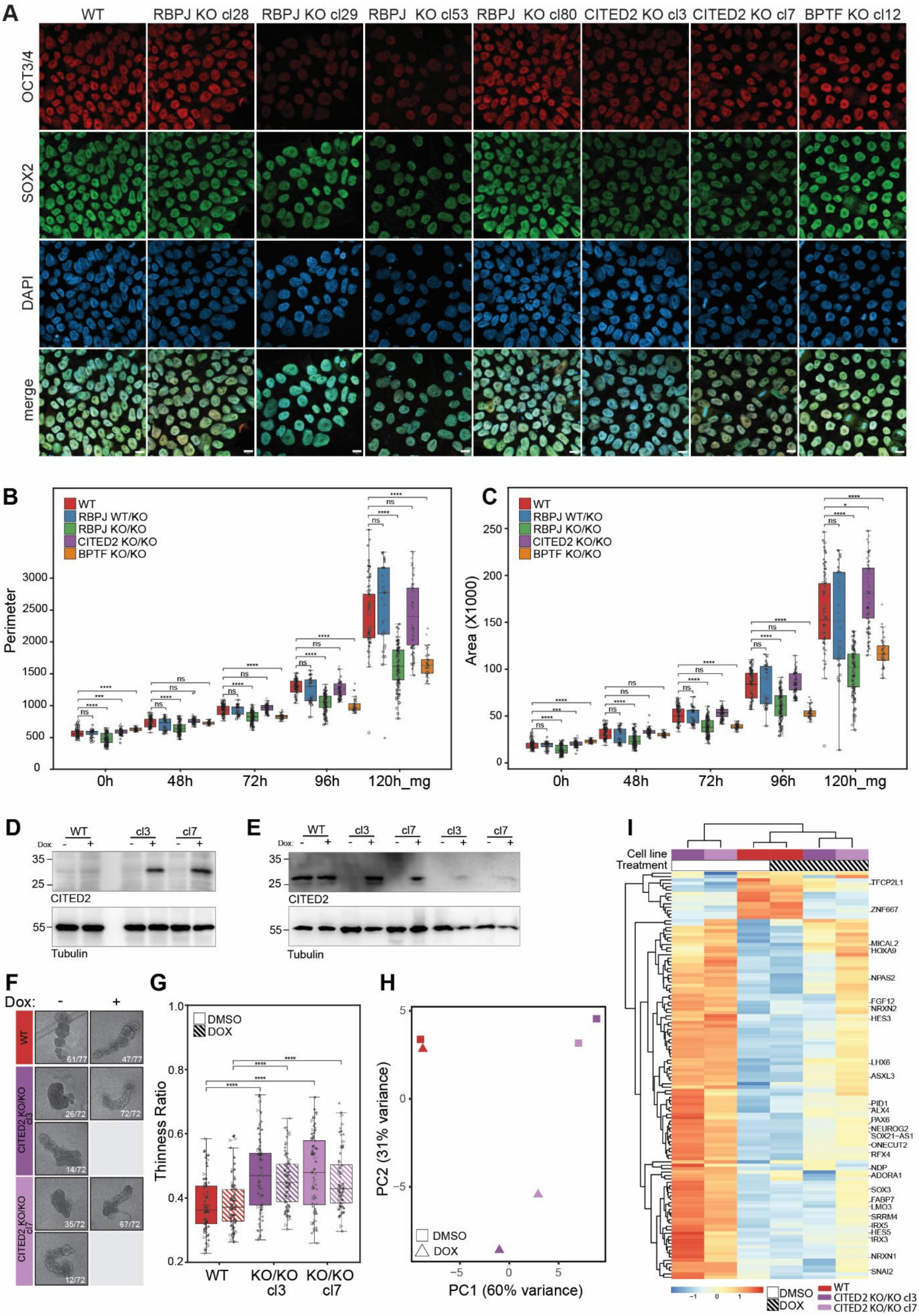

A
WT
RBPJ KO cl28
RBPJ KO cl29
RBPJ KO cl53
RBPJ KO cl80
CITED2 KO cl3
CITED2 KO cl7
BPTF KO cl12
OCT3/4
SOX2
DAPI
merge
B
WT
RBPJ WT/KO
RBPJ KO/KO
CITED2 KO/KO
BPTF KO/KO
Perimeter
0h
48h
72h
96h
120h_mg
C
Area (X1000)
D
Dox:
CITED2
Tubulin
E
F
G
DMSO
DOX
Thinness Ratio
WT
KO/KO cl3
KO/KO cl7
H
PC2 (31% variance)
PC1 (60% variance)
I
Cell line
Treatment
TFCP2L1
ZNF667
MICAL2
HOXA9
NPAS2
FGF12
NRXN2
HES3
LHX6
ASXL3
PID1
ALX4
PAX6
NEUROG2
SOX21-AS1
ONECUT2
RFX4
NDP
ADORA1
SOX3
FABP7
LMO3
SRRM4
IRX5
HES5
IRX3
NRXN1
SNAI2
CITED2 KO/KO cl3
CITED2 KO/KO cl7

Figure S6. Pluripotency and morphological characterization of BPTF, RBPJ, and CITED2 knockout iPSCs and somitoids, related to Figure 5.

(A) Immunofluorescence staining of OCT3/4 and SOX2 with DAPI in undifferentiated wild-type EBL.cl3 iPSCs and BPTF, RBPJ, and CITED2 knockout iPSCs. Scale bar = 10 μm.

(B) Quantification of the perimeter of somitoids across time points generated from wild-type EBL.cl3 iPSCs and RBPJ, CITED2, and BPTF knockout iPSCs. Somitoids were embedded in Matrigel for the final 24h (120h_mg). Each dot represents a single somitoid. Different shapes indicate distinct biological replicates. Reported *p* values are determined with Mann-Whitney-Wilcoxon test two-sided with Bonferroni correction.

(C) Quantification of the area of somitoids across time points generated from wild-type EBL.cl3 iPSCs and RBPJ, CITED2, and BPTF knockout iPSCs. Somitoids were embedded in Matrigel for the final 24h (120h_mg). Each dot represents a single somitoid. Different shapes indicate distinct biological replicates. Reported *p* values are determined with Mann-Whitney-Wilcoxon test two-sided with Bonferroni correction.

(D) Western blot of protein extracts from wild-type EBL.cl3 iPSCs and CITED2 knockout clones with a doxycycline inducible expression for CITED2 integrated. Cells were untreated (DMSO) or treated with doxycycline for 24 hours before collection. Blots were incubated with anti-CITED2 and anti-Tubulin antibodies.

(E) Western blot of protein extracts from Matrigel-embedded somitoids derived from wild-type EBL.cl3 iPSCs and CITED2 knockout clones with a doxycycline inducible expression for CITED2 integrated. Cells were untreated (DMSO) or treated with doxycycline over the course of differentiation. Blots were incubated with anti-CITED2 and anti-Tubulin antibodies.

(F) Representative images of Matrigel-embedded somitoids derived from wild-type EBL.cl3 iPSCs and CITED2 knockout clones with a doxycycline inducible expression for CITED2 integrated. The notation *x/x* indicates the number of somitoids displaying the representative phenotype out of the total number analyzed. If the total is not accounted for, remaining samples exhibited mixed or wild-type-like phenotypes.

(G) Quantification of the elongation ratio of Matrigel-embedded somitoids generated from wild-type EBL.cl3 iPSCs and CITED2 knockout iPSCs with a doxycycline inducible expression for CITED2 integrated. Cells were untreated (DMSO) or treated with doxycycline over the course of differentiation. Each dot represents a single somitoid. Different shapes indicate distinct biological replicates, comprising both knockout clones. Reported *p* values are determined with Mann-Whitney-Wilcoxon test two-sided with Bonferroni correction.

(H) PCA based on the most variable expressed genes in somitoids generated from wild-type EBL.cl3 iPSCs and CITED2 knockout iPSCs with a doxycycline inducible expression for CITED2 integrated. Cells were untreated (DMSO; squared) or treated with doxycycline (triangles) over the course of differentiation.

(I) Heatmap showing scaled expression of differentially expressed genes (p value <0.05 and $\log_2$ FC >2) between Matrigel-embedded somitoids derived from wild-type cells and CITED2 knockout iPSCs, untreated (DMSO) or treated with doxycycline.

Figure S7

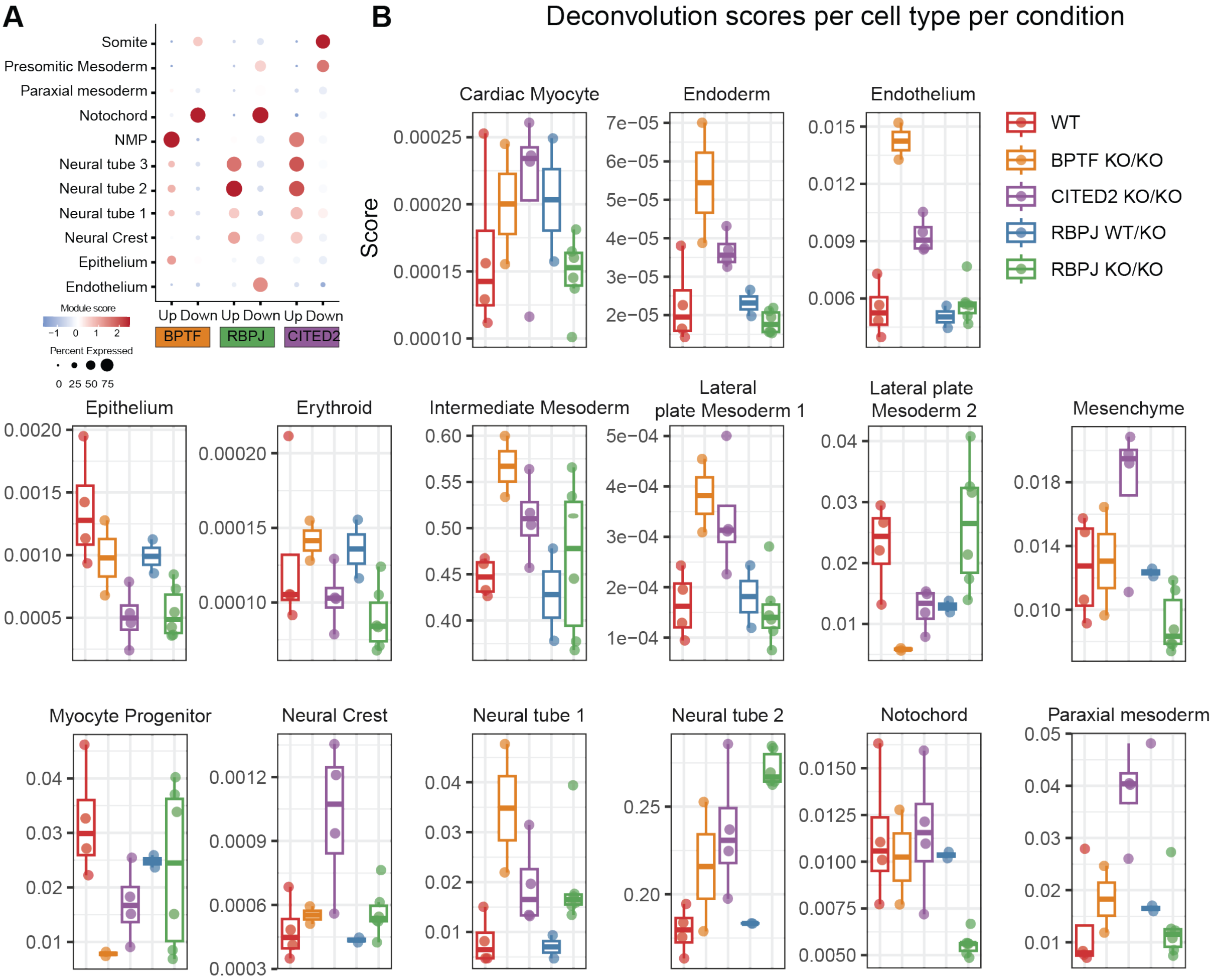

Figure S7. Targeted knockout of BPTF, RBPJ and CITED2 affects gene expression in somitoids, related to Figure 5.

(A) Dot plot of AddModuleScore enrichment across cell types in scRNA-seq of human embryos at gastrulation stage (GSE155121). Module scores were computed from gene sets defined by the differentially expressed genes, separated into up- and downregulated subsets for each knockout. Dot color indicates module score, and dot size reflects the fraction of expressing cells. Only enriched cell types are shown.

(B) Boxplot of cell type predictions inferred by Scaden in knockout samples, using human embryo scRNA-seq as reference. Distributions reflect relative cell type abundances and enable comparison between conditions rather than absolute quantification.

Figure S8

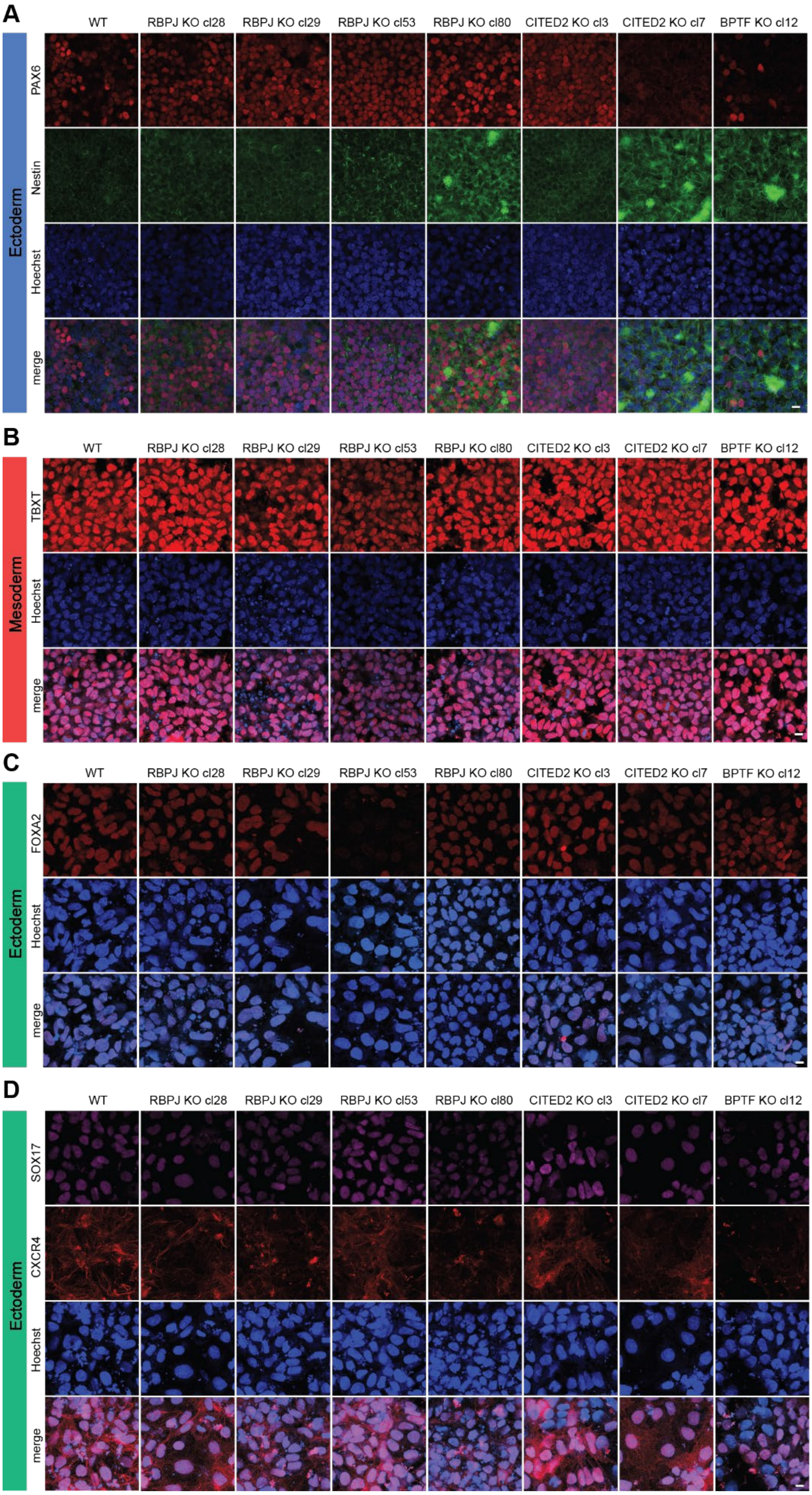
A
WT
RBPJ KO cl28
RBPJ KO cl29
RBPJ KO cl53
RBPJ KO cl80
CITED2 KO cl3
CITED2 KO cl7
BPTF KO cl12
Ectoderm
PAX6
Nestin
Hoechst
merge
B
Mesoderm
TBXT
Hoechst
merge
C
Ectoderm
FOXA2
Hoechst
merge
D
Ectoderm
SOX17
CXCR4
Hoechst
merge

Figure S8. Trilineage differentiation potential of BPTF, CITED2 and RBPJ knockout iPSCs, related to Figure 5.
wild-type EBL.cl3 iPSCs (WT) and BPTF, CITED2 and RBPJ knockout iPSCs cultured in mTeSR Plus medium were subjected to directed differentiation toward ectoderm, mesoderm, and endoderm lineages. Representative immunofluorescence images show expression of lineage-specific markers: (A) ectoderm (PAX6/Nestin), (B) mesoderm (Brachyury/TBXT), and (C and D) endoderm (SOX17/FOXA2/CXCR4). Nuclei were stained with Hoechst. Scale bar = 10 µm.

Figure S9

A

	Wild-type iPSCs
D8S1179	13,15
D21S11	28,31.2
D7S820	8,10
CSF1PO	11,12
D3S1358	16,18
TH01	7,9.3
D13S317	13,13
D16S539	13,14
D2S1338	18,24
D19S433	14,14
vWA	14,18
TPOX	8,8
D18S51	13,15
AMEL	X,X
D5S818	11,12
FGA	22,24

B

	SANi001-A	SANi002-A	UCLi017-A
D8S1179	13,14	12,14	11,14
D21S11	28,29	30,30	28,30
D7S820	10,11	9,12	10,11 **
CSF1PO	9,11	10,11	-*
D3S1358	17,18	14,15	15,17
TH01	8,9.3	7,7	7,9.3
D13S317	12,12	12,13	12,12
D16S539	11,12	11,12	12,13
D2S1338	19,20	19,23	-*
D19S433	13,15	15,15	14,15**
vWA	16,17	16,17	16,19
TPOX	8,8	8,11	8,8
D18S51	13,15	12,16	-*
AMEL	X,Y	X,X	X,Y
D5S818	11,12	11,12	11,11
FGA	22,23	23,23	-*

C

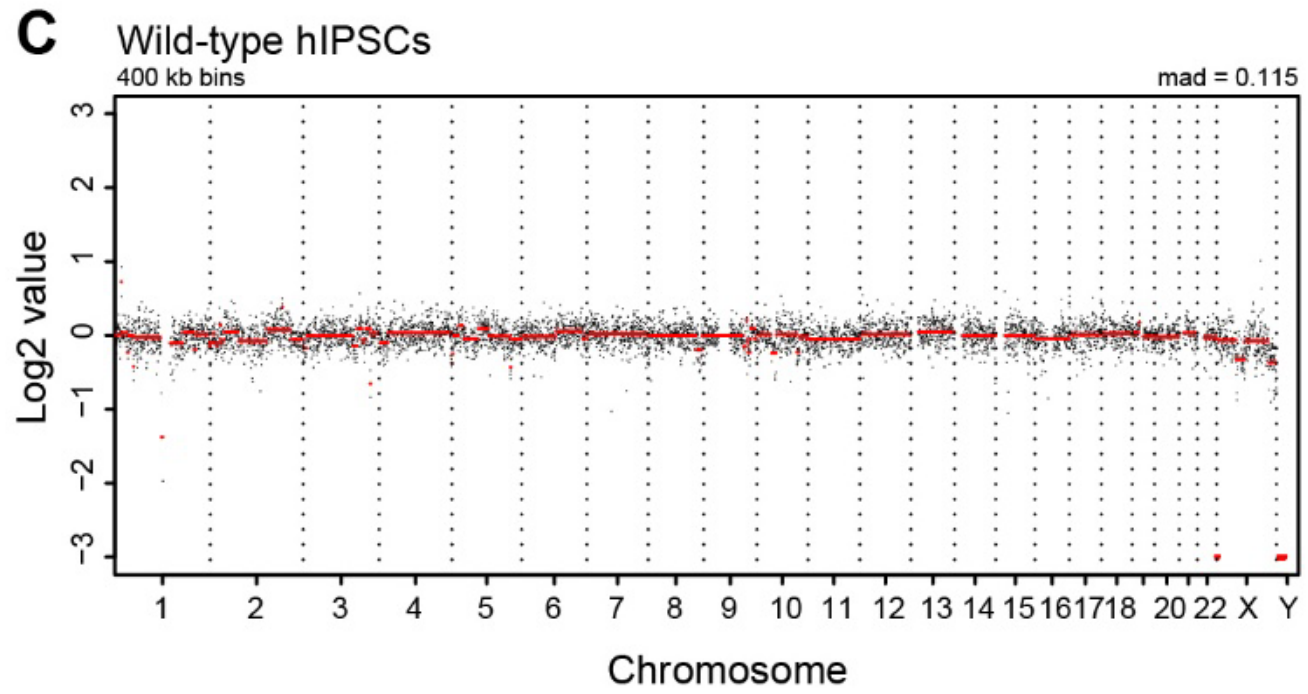

D

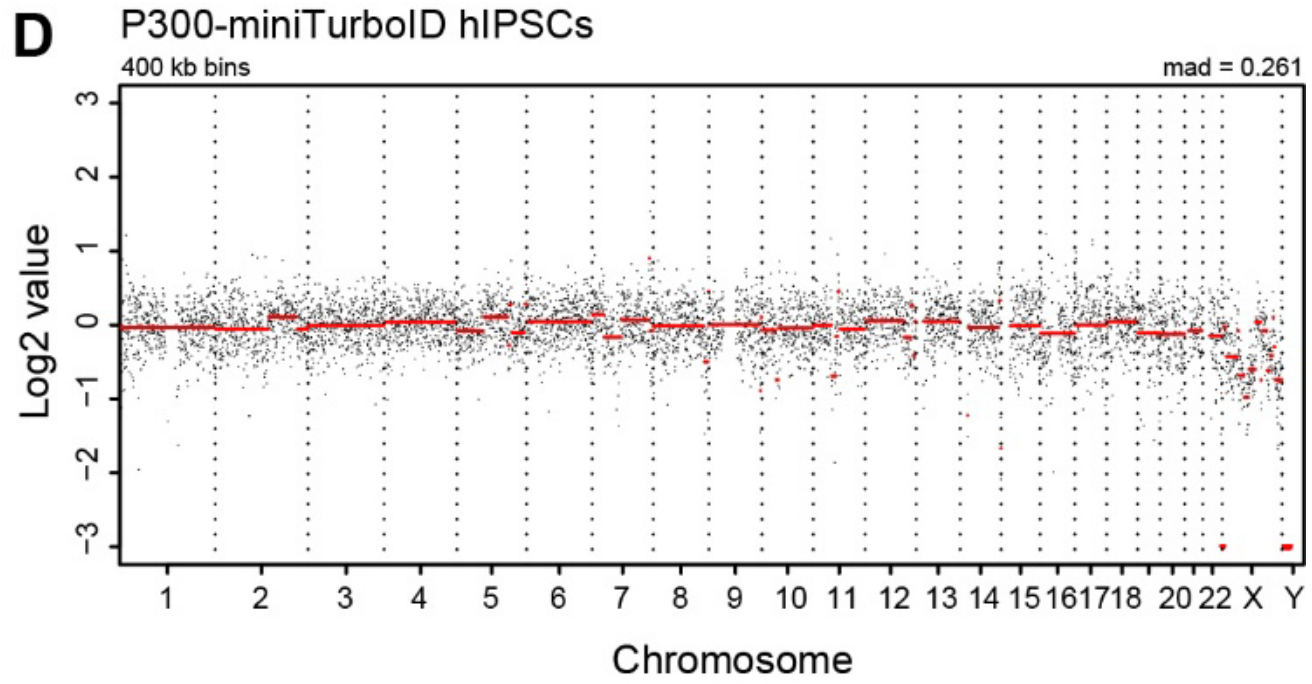

Figure S9. STR and copy number profile validation in iPSCs, related to Methods.
(A) STR analysis of the wild-type EBL.cl3 iPSCs used in the main figures of this study.
(B) STR analysis of SANi001-A, SANi001-2-A, and UCLi017-A iPSCs. ** weak signal, * no signal.
(C) Copy number profile of wild-type EBL.cl3 iPSCs.
(D) Copy number profile of EBL.cl3 iPSCs expressing p300-V5-miniTurboID-T2A-puro.

Supplemental tables

Table S8. Primer sequences, related to Methods.

Name	Sequence 5'-3'
169_RBPJ_outside_F	TTCTTTTGCTCCCTCCCCAC
170_RBPJ_outside_R	ACATTGGGATATGGCAGGTACC
171_RBPJ_inside_R	AAAAGGCTCCCCACTGCTG
175_BPTF_outside_F	ATCCCGCCCCTTGAATTTCC
176_BPTF_outside_R	GCTCTGCTACATCAATCACGAAG
177_BPTF_inside_F	TGGGGATTTGCTTTGCTGTG
190_CITED2_outside_F	TTCACGCCGAAGAAGTTGG
191_CITED2_outside_R	GCCCTTTTCACTTCCAGGAC
