# Supplementary material for: From stem cells to somites: Revealing genetic and exogenous factors of human embryogenesis
Source: Stem Cell Reports. 2026 Aug 6;21(9):103023. doi: 10.1016/j.stemcr.2026.103023 (PMC13555414; doi:10.1016/j.stemcr.2026.103023)
Supplement: Supplementary file 2 [file mmc9.pdf]

STEM CELL REPORTS
Article

ISSCR

OPEN ACCESS

From stem cells to somites: Revealing genetic and exogenous factors of human embryogenesis

Maria Teresa Alejo-Vinogradova,[1,7] Yunna E. Strøm,[1,7] Lieke A. Lamers,[1,7] Yolanda W. Chang,[2,7] Martijn J. Hendriksen,[2] Mariya Moosajee,[3,4,5] Michiel Vermeulen,[1,6,8,*] and Suzan Stelloo[1,2,8,9,*]

[1]Department of Molecular Biology, Faculty of Science, Radboud Institute for Molecular Life Sciences, Oncode Institute, Radboud University Nijmegen, 6525 GA Nijmegen, the Netherlands
[2]Department of Urology, Radboud Institute for Medical Innovation, Radboud University Medical Center, 6525 GA Nijmegen, the Netherlands
[3]UCL Institute of Ophthalmology, University College London, London EC1V 9EL, UK
[4]The Francis Crick Institute, London NW1 1AT, UK
[5]Moorfields Eye Hospital NHS Foundation Trust, London EC1V 2PD, UK
[6]Division of Molecular Genetics, Netherlands Cancer Institute, 1066 CX Amsterdam, the Netherlands
[7]These authors contributed equally
[8]These authors contributed equally
[9]Lead contact
*Correspondence: michiel.vermeulen@ru.nl (M.V.), suzan.stelloo@radboudumc.nl (S.S.)
https://doi.org/10.1016/j.stemcr.2026.103023

SUMMARY

Stem-cell-based human embryo models offer an ethically tractable platform for studying early human development. This study employs somitoids, three-dimensional models of human somitogenesis, to investigate how transcriptional programs and culture conditions influence somite formation and segmentation. We show that pre-differentiation culture medium impacts the developmental potential of induced pluripotent stem cells (iPSCs), with StemFit medium and Matrigel embedding outperforming mTeSR Plus medium in generating robust somite-like structures. Strikingly, these differences arise despite only subtle changes in transcriptomic and time-resolved proteomic profiles. P300-based proximity labeling also reveals a largely overlapping set of chromatin-associated regulators across iPSC conditions. In somitoids, enhancer-associated profiling highlights factors linked to somitogenesis, including MESP2 and TBX6. Knockout of three identified regulators, BPTF, RBPJ, and CITED2, demonstrate their essential roles in somite formation. Together, these findings highlight how culture conditions and enhancer-associated networks influence early human development and demonstrate somitoids as a scalable system for functional genomics.

INTRODUCTION

In recent years, the study of embryonic development has advanced significantly through the use of *in vitro* models derived from induced pluripotent stem cells (iPSCs). These models recapitulate key stages of mammalian development and are especially valuable in human research, where ethical restrictions limit access to embryos at early stages. Among these models are somitoids, three-dimensional structures that mimic somite formation. Somites are epithelial blocks derived from the paraxial mesoderm that give rise to tissues such as the vertebral column, skeletal muscles, and dermis (Miao and Pourquie, 2024).

Several protocols have been developed to generate 3D somite-like structures and neuronal lineages from iPSCs (Miao et al., 2023; Sanaki-Matsumiya et al., 2022; Yamanaka et al., 2023). While these approaches yield similar morphological outcomes, the protocols vary considerably in culture conditions and differentiation strategies, reflecting the still poorly understood nature of the mechanisms guiding human axial development. These studies have contributed to further characterizing key transcription factors involved in somite formation, including HES7, TBX6, and MESP2, which regulate the segmentation clock and presomitic mesoderm identity. Additionally, genes such as UNCX, TBX18, and TCF15, which mark segment polarity and somite maturation, are also characteristically present in *in vitro* models of somitogenesis (Miao et al., 2023; Sanaki-Matsumiya et al., 2022; Yamanaka et al., 2023). Comparative analysis of single-cell RNA sequencing (scRNA-seq) data from human embryos demonstrates that these *in vitro* models recapitulate a broad spectrum of cell types observed during early somitogenesis (Yamanaka et al., 2023). This cellular diversity underscores the relevance of these models for studying lineage specification and identifying novel factors important for neurogenesis and segmentation during development.

Importantly, environmental inputs such as extracellular matrix components and culture medium composition have emerged as critical modulators of somitoid formation (Sanaki-Matsumiya, 2022; Yamanaka et al., 2023). The addition of Matrigel enhances the structural organization and segmentation of somitogenesis models in both human and mouse, indicating that the microenvironment significantly influences morphogenesis (Miao et al., 2023; Sanaki-Matsumiya et al., 2022; van den Brink et al., 2020; Veenvliet et al., 2020; Yamanaka et al., 2023). Interestingly,

Published by Elsevier Inc. on behalf of International Society for Stem Cell Research.
This is an open access article under the CC BY license (http://creativecommons.org/licenses/by/4.0/).

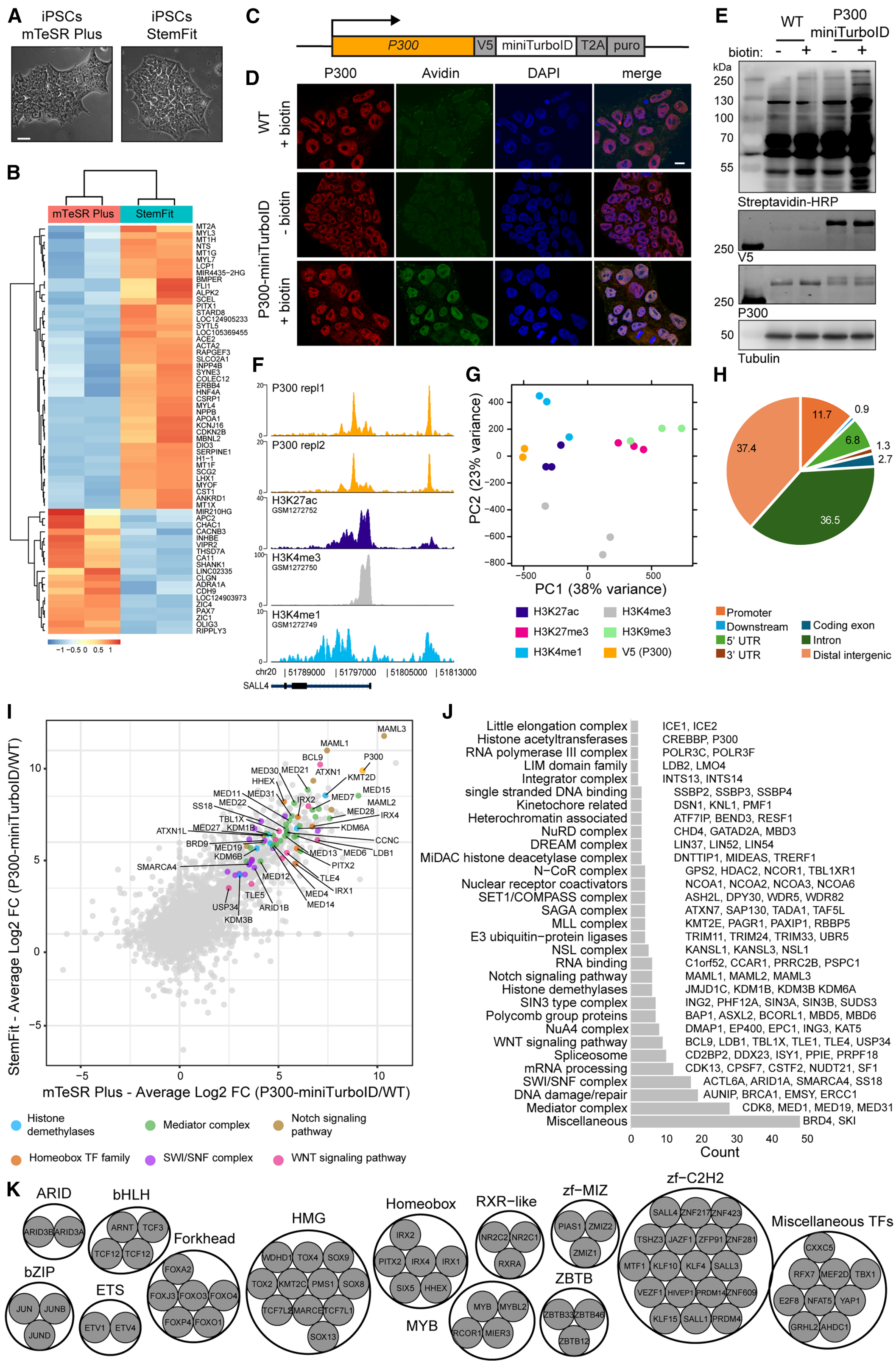

(legend on next page)

while Matrigel improves the architectural fidelity of these structures, somitogenesis models generated without Matrigel still give rise to comparable cell populations (Miao et al., 2023; Sanaki-Matsumiya et al., 2022; Yamanaka et al., 2023). This highlights the broader relevance of biophysical and biochemical inputs in early human development and emphasizes the importance of systematically dissecting how specific culture conditions shape morphogenetic outcomes.

In this study, we evaluated how stem cell maintenance conditions affect the competence of iPSCs to differentiate into somitoids. We observed that although both mTeSR Plus and StemFit Basic04CT (hereafter referred to as StemFit) medium maintain pluripotency at the transcriptomic level, cells cultured in StemFit show a greater capacity to form segmented structures, showing how pre-differentiation conditions impact developmental potential. Together, these findings underscore the importance of environmental cues, showing that even subtle differences in medium composition shape developmental outcomes *in vitro*. To more precisely explore which transcription factors are active during somite formation, we employed a strategy based on the fusion of the acetyltransferase P300 with the biotin ligase miniTurboID, enabling proximity labeling of proteins present at transcriptionally active regions in the genome. From this dataset, we selected three factors—BPTF, RBPJ, and CITED2—whose knockout resulted in disrupted somite formation, confirming their functional roles in human somitogenesis. Altogether, this work demonstrates how culture conditions and targeted molecular profiling can uncover regulators of somitogenesis and deepen our understanding in somite formation *in vitro*.

RESULTS

iPSC pluripotency and enhancer interactome are largely maintained under different culture conditions

Previous research has reported differences in stem cell behavior under varying maintenance conditions (Sanaki-Matsumiya, 2022). To evaluate the impact of maintenance conditions on pluripotency and somitoid differentiation efficiency, human iPSCs were cultured in either mTeSR Plus or StemFit medium (Nakagawa et al., 2014) prior to differentiation. Both media support long-term maintenance of undifferentiated human iPSCs as confirmed by compact colony morphology (Figure 1A) and stable expression of core pluripotency markers, including OCT4, SOX2, and NANOG (Figures S1A and S1B). Despite this, differential

Figure 1. iPSC pluripotency and enhancer interactome are largely maintained under different culture conditions
(A) Brightfield images of iPSCs cultured in mTeSR Plus or StemFit medium. Scale bars: 20 μm.
(B) Heatmap showing scaled expression of 62 differentially expressed genes (p value < 0.05 and $\log_2$ FC > 2) between iPSCs cultured in mTeSR Plus and StemFit medium.
(C) Schematic representation of CRISPR-Cas9-mediated endogenous gene tagging of the C-terminus of *EP300* gene with V5-miniTurboID followed by a cleaving peptide (T2A) and puromycin coding sequence.
(D) Immunofluorescence staining for P300 and biotinylated proteins in wild-type iPSCs (WT) stimulated with biotin and P300-miniTurboID expressing iPSCs stimulated without or with biotin. Scale bars: 10 μm.
(E) Western blot of protein extracts from wild-type iPSCs and P300-miniturboID-expressing cell line without (−) and with (+) treatment of biotin. Blots were incubated with Streptavidin-HRP, anti-P300, anti-V5, and anti-Tubulin antibodies.
(F) Genome browser snapshot showing P300, H3K27ac, H3K4me3, and H3K4me1 ChIP-seq tracks at the *SALL4* gene locus. The *y* axes indicate the ChIP-seq signal in fragments per kilobase per million (FPKM) reads mapped.
(G) PCA of ChIP-seq samples based on the read counts at merged binding sites (consensus peaks) across all samples. Two publicly available ChIP-seq datasets from iPSCs were included, covering histone modifications associated with repressed chromatin (H3K27me3 and H3K9me3), active chromatin (H3K27ac), active enhancers (H3K4me1), and active promoters (H3K4me3).
(H) Genomic distribution of P300 binding sites across defined genomic features, based exclusively on peaks shared between replicate 1 and replicate 2.
(I) Scatterplot showing the mean $\log_2$ FC for proteins enriched in streptavidin affinity purifications of biotin-treated P300-miniTurboID-expressing cells compared to biotin-treated wild-type cells, comparing two different media conditions. $\log_2$ FC in each condition is calculated relative to a biotin-treated wild-type control and averaged across two independent replicates. Only proteins identified in both replicates are included or retained if identified in both replicates of the other condition. The *x* axis indicates the average $\log_2$ FC of iPSCs cultured in mTeSR Plus, and the *y* axis indicates the average $\log_2$ FC of iPSCs cultured in StemFit. Preselected proteins are color-coded based on their association with specific protein complexes, signaling pathways, or protein families.
(J) Bar chart grouping non-transcription factors identified in all independent replicates (in both replicates of each medium with a $\log_2$ FC > 2 and p value <0.05) based on their molecular function, in association with specific protein complexes, signaling pathways, or protein families. Each bar represents the number of interactors within a given category.
(K) Nested bubble tree displaying transcription factors identified in all independent replicates (in both replicates of each medium with a $\log_2$ FC > 2 and p value <0.05) grouped by family. Transcription factors belonging to families represented by only a single identified member are grouped under a "Miscellaneous."

gene expression analysis identified 62 significant genes between mTeSR Plus and StemFit-maintained cells (Figure 1B). Gene set enrichment analysis (GSEA) revealed that these genes were associated with biological processes related to cellular metal homeostasis (Figure S1C). Notably, several metallothionein (MT) family members, including *MT1F*, *MT1G*, *MT1H*, *MT1X*, and *MT2A*, were markedly upregulated in StemFit-cultured iPSCs (Figure 1B). These MT genes are activated in response to various stimuli, including exposure to heavy metals (e.g., zinc and copper), oxidative stress, and steroid hormones, and are primarily regulated by transcription factors such as metal-responsive transcription factor 1 (MTF1) and the glucocorticoid receptor (Coyle et al., 2002; Gunther et al., 2012). In contrast, iPSCs cultured in mTeSR Plus showed enrichment of neurodevelopmental and skeletal-muscle-related pathways (Figure S1C), with upregulation of key genes including *OLIG3*, *ZIC1*, and *PAX7* (Figure 1B). Consistent with the differential gene expression analysis, whole proteome profiling showed largely similar proteomes between the conditions (Figure S1D). To assess whether maintenance medium influences differentiation potential, iPSCs cultured in either mTeSR Plus or StemFit were subjected to 2D trilineage differentiation toward ectoderm, mesoderm, and endoderm (Figure S2). Both conditions supported efficient trilineage differentiation, with no major differences observed between ectodermal and mesodermal marker expression. However, endoderm differentiation revealed differences, with more homogeneous SOX17 and FOXA2 expression in mTeSR-Plus-derived iPSCs as compared to StemFit-derived iPSCs (Figure S2B). These changes, although modest, suggest that iPSC maintenance medium may subtly influence lineage bias and possibly shape the cells' differentiation potential even before overt lineage commitment.

To further investigate gene expression regulation in iPSCs in both culture conditions, we profiled the proximal proteome of active enhancers using P300 as a bait protein, a well-established marker of active enhancers (Figure S3A). Previously, we made use of this approach in mouse embryonic stem cells and gastruloids (Stelloo et al., 2024). To this end, we tagged endogenous P300 with miniTurboID and confirmed the functionality of the fusion protein (Figure 1C). Immunofluorescence analysis revealed nuclear avidin-FITC staining after biotin stimulation, confirming successful biotinylation of proteins proximal to P300 at its expected localization (Figure 1D). Biotin ligase activity was further confirmed by western blotting using streptavidin-HRP, which revealed a characteristic smear of biotinylated proteins in the presence of exogenous biotin (Figure 1E). In addition, a distinct band corresponding to the tagged P300 protein was also detected, confirming expression in the heterozygously tagged line (Figure 1E). Importantly, tagging did not interfere with P300's chromatin binding activity, as evidenced by tens of thousands of high-confidence peaks identified by chromatin immunoprecipitation sequencing (ChIP-seq), predominantly located in distal intergenic and intronic regions (Figures 1F–1H; Figures S3B and S3C). Transcription factor motif enrichment analysis revealed a significant overrepresentation of binding sites for pluripotency-associated transcription factors, including OCT4, SOX2, and NANOG, alongside motifs for RFX and SMAD family members (Figure S3C). Together, these results validate the functionality of the tagged P300 transgene.

Following the validation of the P300-miniTurboID endogenous tagging, iPSCs were cultured in either mTeSR Plus or StemFit medium for several passages prior to performing P300 proximity labeling experiments and subsequent quantitative mass spectrometry analysis. To identify proteins specifically enriched in the proximity of P300, we compared biotin-treated P300-miniTurboID-expressing cells to biotin-treated wild-type controls (Figures S3D and S3E). Correlation analysis of the resulting $\log_2$ fold change ($\log_2$ FC) values revealed strong concordance between biological replicates (Figures S3F and S3G). Proteins consistently enriched in both biological replicates were compiled into a high-confidence consensus list (p value <0.05 and a $\log_2$ FC > 2 in at least one biological replicate and a $\log_2$ FC > 1 in the other replicate; Table S1 [mTeSR Plus] and Table S2 [StemFit]). As expected, P300 itself emerged as the most prominent outlier in both datasets (Figure 1I; Figures S3D and S3G). The majority of P300-associated proteins were shared between mTeSR Plus and StemFit conditions, consistent with the expectation that fundamental components of the enhancer regulatory machinery are broadly overlapping across culture conditions, cellular contexts, and even species (Figure 1I). For example, from the well-established protein complexes known to function at enhancers, we identified 28 Mediator subunits and 17 SWI/SNF complex members (Figure 1J). The dataset also includes a rich repertoire of chromatin-associated proteins, including histone modifiers (CBP [CREBBP], KDM1B, KDM3B, KDM6B), transcriptional coactivators (NCOA1/2/3/6), and components of additional regulatory complexes, such as NuRD, DREAM, N-CoR, and NuA4 (Figure 1J). We also identified coregulators of WNT (BCL9, TLE1/4/5) and Notch (MAML1/2/3) signaling within the P300 interactome (Figure 1J). In addition, numerous transcription factors were identified, many of which are well characterized in the context of stem cell biology (Figure 1K). These include pluripotency-associated factors such as NANOG and FOXO4, cell fate regulators like RFX7, as well as members of the HOX family and nuclear receptors (NR2C1/2 and RXRa). Beyond overlapping P300 proximal proteins, a subset of proteins showed clear medium-specific enrichment (Figures S3H and S3I). In mTeSR

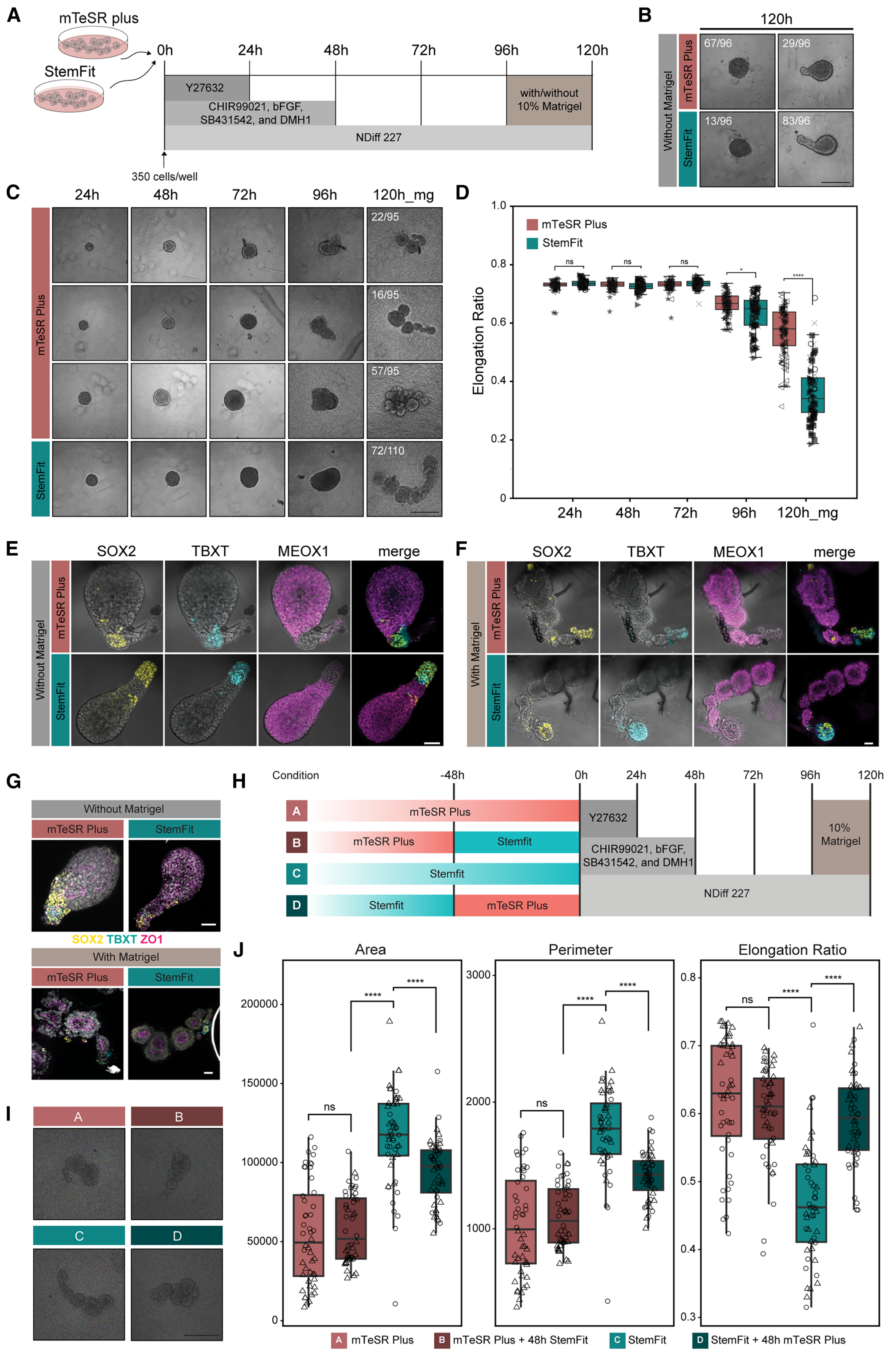

(legend on next page)

Plus, transcription factors such as GATA4 and GATA6 were selectively enriched, along with transcription factors linked to WNT signaling (SOX17, WNT5B, and CER1). In contrast, StemFit-specific enrichments included FOXA1, SOX21, and members of the ETS family of transcription factors (ETS1, ETS2, and ERG) (Figures S3H and S3I). Taken together, our results highlight gene expression programs and P300 interactomes that are shared across conditions, as well as medium-specific genes and interactors. In summary, we observed subtle differences on gene expression and a largely shared P300 interactome between the different media.

Impact of maintenance medium on somitoid formation efficiency

To assess the impact of maintenance medium on somitoid formation efficiency, iPSCs cultured in either mTeSR Plus or StemFit were differentiated toward somitoids and monitored daily by brightfield microscopy to assess morphological changes and somite-like structure formation. Somitoid differentiation was performed both in the absence and presence of Matrigel, with Matrigel added only in the last 24 h of differentiation (Figure 2A). Both mTeSR-Plus- and StemFit-pre-cultured iPSCs formed aggregates; however, distinct somitoid morphologies were observed in the absence and presence of Matrigel (Figures 2B and 2C). Without the addition of Matrigel in the last 24 h, differentiated StemFit-derived iPSCs showed a more elongated morphology compared to mTeSR-Plus-derived iPSCs, as quantified by perimeter and area measurements as well as the calculated elongation ratio, where an elongation ratio closer to one reflects a more rounded morphology (Figure 2B; Figure S4A). In the presence of Matrigel, mTeSR-Plus-derived cultures often failed to develop a posterior "body" with a string of somites by day 5 (Figure 2C). These aggregates from mTeSR-Plus-derived cultures on day 5 appeared more compact with limited axial elongation (Figure 2D; Figures S4B and S4C). Cells maintained in StemFit formed segmentation-like structures, typically producing 4–6 segments per aggregate. No additional somites were observed beyond day 5. Additionally, immunostaining confirmed the expression of MEOX1, a somite marker, in both Matrigel-free and Matrigel-present conditions (Figures 2E and 2F). In the presence of Matrigel, ZO1 was enriched at the inner surface of somite-like structures (Figure 2G). This is consistent with previous reports indicating that somite cell populations are specified independent of Matrigel (Miao et al., 2023; Sanaki-Matsumiya et al., 2022). Overall, this indicates that, even though both media support the maintenance of iPSCs in an undifferentiated, pluripotent state, the choice of medium can still influence downstream differentiation outcomes. To

Figure 2. Pre-culturing iPSCs in stemFit medium enhances somitoid formation and coincides with enrichment of somitogenesis regulators at enhancers

(A) iPSCs were maintained in either mTeSR Plus or StemFit medium. To induce differentiation, cells were cultured in non-adherent conditions in somitoid induction medium (NDiff227 supplemented SB431542 CHIR99021, DMH1, bFGF, and Y-27632 ROCK inhibitor) and at 96 h in NDiff227 medium either with or without 10% Matrigel.

(B) Representative images of somitoids derived from iPSCs cultured in mTeSR Plus or StemFit medium, not embedded in Matrigel during the final 24 h. The notation *x/x* indicates the number of somitoids displaying the representative phenotype out of the total number analyzed. Scale bars: 200 μm.

(C) Representative images of somitoids derived from iPSCs cultured in mTeSR Plus or StemFit medium over a 5-day time course. The day 5 time point corresponds to somitoids cultured in Matrigel (120h_mg). The notation *x/x* indicates the number of somitoids displaying the representative phenotype out of the total number analyzed. If the total is not accounted for, remaining samples exhibited other phenotypes. Scale bars: 200 μm.

(D) Quantification of the elongation ratio of somitoids generated from wild-type cells pre-cultured in either mTeSR Plus or StemFit medium. The day 5 time point corresponds to somitoids cultured in Matrigel (120h_mg). Each dot represents a single somitoid. Different shapes indicate distinct biological replicates. Reported *p* values are determined with Mann-Whitney-Wilcoxon test two-sided with Bonferroni correction.

(E) Immunofluorescence staining of SOX2, TBXT, and MEOX1 with DAPI in somitoids at 120 h cultured without Matrigel. Scale bars: 50 μm.

(F) Immunofluorescence staining of SOX2, TBXT, and MEOX1 with DAPI in Matrigel-embedded somitoids at 120 h. Scale bars: 50 μm.

(G) Immunofluorescence staining of SOX2, TBXT, and ZO1 with DAPI in somitoids at 120 h, cultured with or without Matrigel. Scale bars: 50 μm.

(H) Schematic of experimental design showing medium switch 48 h prior to aggregation and somitoid differentiation or continuous iPSC culture in mTeSR Plus or StemFit medium.

(I) Representative images of day 5 Matrigel-embedded somitoids generated from wild-type EBL.cl3 iPSCs pre-cultured in different culture conditions (A–D). Scale bars: 200 μm.

(J) Quantification of the area, perimeter, and elongation ratio of somitoids generated from wild-type EBL.cl3 iPSCs pre-cultured in different culture conditions (A–D), as outlined in the experimental schematic in (H). Each dot represents a single somitoid. Different shapes indicate distinct biological replicates. Reported *p* values are determined with Mann-Whitney-Wilcoxon test two-sided with Bonferroni correction.

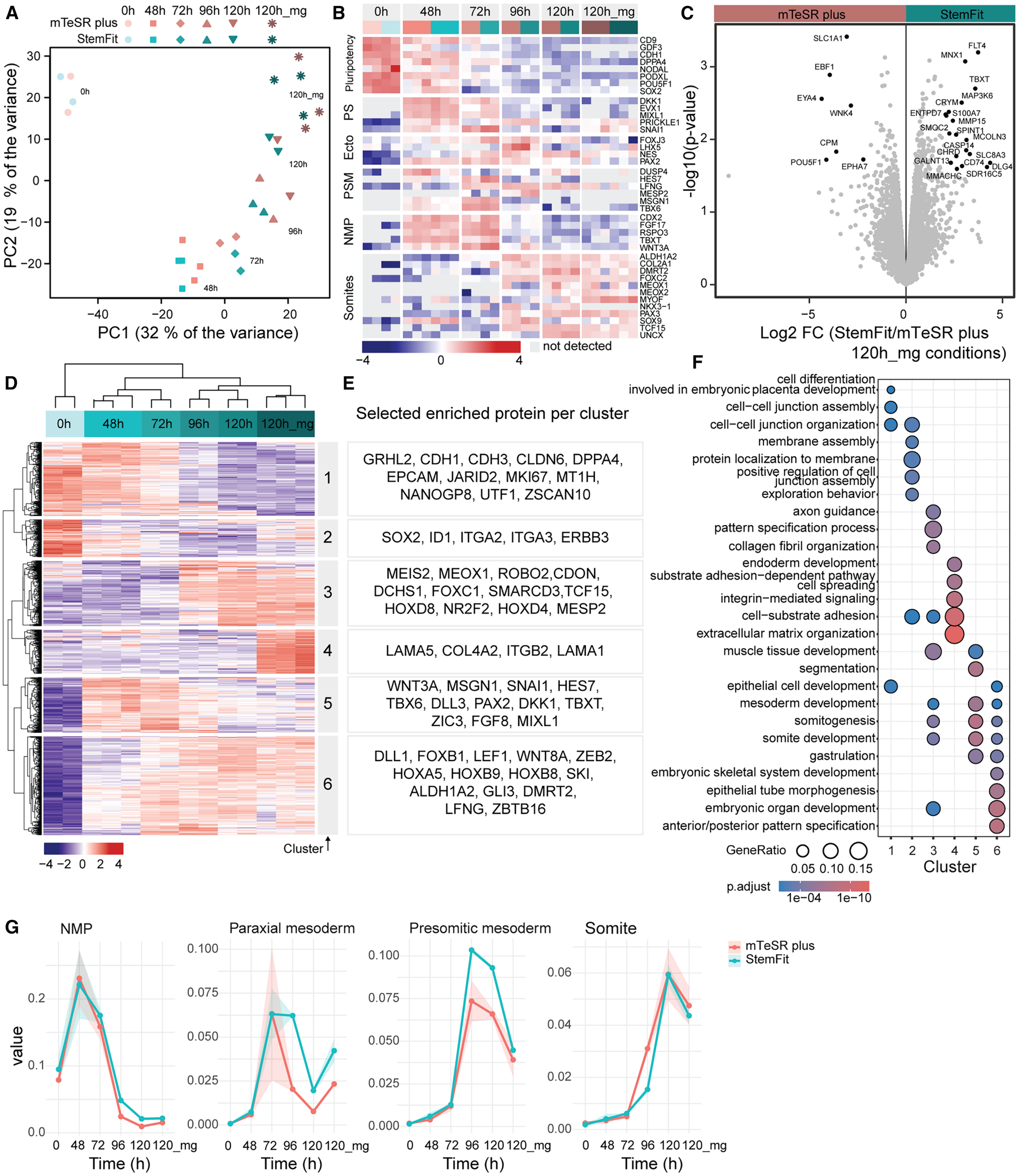

Figure 3. Dynamic proteome changes during somitoid differentiation

(A) PCA based on the 1,000 most variably expressed proteins, with samples colored by condition and shapes indicate time points. 120h_mg indicates the time point corresponding to somitoids cultured in Matrigel.

(B) Heatmap showing scaled expression of selected proteins associated with embryonic development.

(legend continued on next page)

further distinguish whether these effects reflect long-term adaptation or short-term exposure to culture conditions, we performed 48-h medium-switch experiments prior to differentiation (Figure 2H). StemFit-to-48-h mTeSR Plus switching reduced somitoid formation, while mTeSR Plus-to-StemFit switching did not fully restore segmentation (Figures 2I and 2J). These results indicate that robust somite formation requires continuous culture in StemFit conditions. Importantly, experiments in three additional iPSC lines independently confirmed improved somitoid morphology and segmentation using StemFit-derived iPSCs compared to mTeSR-Plus-derived iPSCs (Figures S4A–S4F).

Given the clear morphological differences in our iPSCs derived structures, we performed RNA-seq on somitoids derived from both maintenance conditions. Principal-component analysis (PCA) revealed clear transcriptional separation between somitoids derived from mTeSR Plus and those from StemFit (Figure S4J). Although a limited number of genes, including *FSHR*, *KDR*, and *SYT2*, were upregulated in the mTeSR Plus condition, these genes did not surpass the statistical significance threshold (p value < 0.05 and $\log_2$ FC > 2) following log fold change shrinkage (lfcShrink). Nonetheless, their differential expression may reflect a transcriptional bias toward neurovascular lineage that potentially contributes to the observed phenotype. In contrast, somitoids derived from iPSCs maintained in StemFit medium showed expression of genes involved in anterior-posterior patterning and somitogenesis (Figures S4K and S4L). In particular, several HOX genes, including *HOXC8*, *HOXC9*, and *HOXD1* were upregulated. Additionally, multiple secreted factors associated with the segmentation clock and wavefront signaling, including *FGF4* (Anderson et al., 2020), *WNT3A* (Aulehla et al., 2003), and *DKK1* (Pedersen et al., 2011), also showed increased expression. These findings correspond with the presence of somite-like morphology observed at day 5 of the differentiation protocol. In summary, the relatively small number of differentially expressed genes indicates that the overall transcriptomic profiles of somitoids from both conditions are largely similar, suggesting that iPSCs cultured in either medium retain their differentiation potential. However, the notably lower expression of key somitogenic markers (Figure S4M), such as WNT3A and UNCX, in the somitoids derived from mTeSR-Plus-cultured iPSCs suggests that critical lineage-specifying pathways are not effectively activated, which may underlie the observed morphological differences. These findings highlight the critical role of pre-differentiation culture conditions in ensuring proper activation of developmental programs and successful somitoid formation.

Dynamic proteome changes during somitoid differentiation

To complement the RNA-seq analysis and capture molecular dynamics, we performed time-resolved quantitative proteomics across somitoid differentiation. On average, 10,445 proteins were quantified per time point, with nearly 8,000 proteins consistently detected across all samples (Figures S5A and S5B). PCA revealed a clear progression over time, with samples organizing according to differentiation stage rather than culture condition (Figure 3A). Analysis of lineage-specific markers further supported this differentiation. Proteins associated with pluripotency were abundantly expressed in undifferentiated iPSCs (time point 0 h), whereas early mesoderm markers, such as TBXT, were enriched at intermediate time points, followed by the emergence of somite-associated proteins at later stages (Figure 3B). At 120 h, relatively few proteins were differentially expressed between somitoids derived from mTeSR-Plus- and StemFit-cultured iPSCs (Figure 3C). These differences included mesodermal markers such as TBXT and TBX6, which showed consistent directional changes at the RNA level (Figure S5C). mTeSR-Plus-derived somitoids showed higher expression levels of proteins such as ZIC1 and EBF1 (Figure S5C). Overall, these data indicate that molecular differences between conditions are modest. Given the canonical bead-on-a-string morphology from StemFit-derived iPSCs, we focused subsequent analyses on time-resolved dynamics within this condition. Differential protein expression analysis identified distinct groups of proteins with coordinated temporal expression patterns, corresponding to stage-specific biological processes (Figures 3D–3F). Early time points were enriched for proteins associated with pluripotency and cell-cell junction organization, whereas intermediate and later time points

(C) Volcano plot showing changes in protein expression between Matrigel-embedded somitoids generated from iPSCs maintained in mTeSR Plus or StemFit medium (120h_mg). Log_2 fold change of LFQ intensities is plotted against $-\log_{10}(p \text{ value})$. Proteins with an absolute $\log_2$ FC > 2 and adjusted p value <0.05 are highlighted in black.
(D) Heatmap showing scaled expression of 980 significantly differentially expressed proteins across all time points in somitoids generated from StemFit-cultured iPSCs, organized into six clusters.
(E) Text boxes displaying selected representative proteins for each cluster identified in the heatmap as shown in (D).
(F) Gene ontology enrichment analysis for each protein cluster identified in the heatmap (D).
(G) Line plot showing Scaden-inferred cell-type composition across differentiation time points using scRNA-seq from human embryos at gastrulation stage as a reference (GSE155121), where values represent relative cell-type proportions.

were enriched for processes related to mesoderm development, somitogenesis, and anterior/posterior pattern specification. This included multiple HOX proteins, predominantly associated with trunk identity (Figure S5D). Cluster 4, characterized by highest expression in Matrigel-cultured somitoids at 120 h, was dominated by extracellular matrix proteins, including laminins and collagens, which were also detected in Matrigel-only controls, indicating that part of this signal likely reflects matrix-derived proteins (Table S3). Nonetheless, some proteins within this cluster may still represent *bona fide* biological signals. To further resolve the cellular composition underlying these proteomic dynamics, we performed deconvolution analysis using scRNA-seq of human embryos at gastrulation stage (Zeng et al., 2023). This revealed a progressive shift from neuromesodermal progenitors (NMPs) toward presomitic mesoderm (PSM) and somitogenic identities over time (Figures 3G and S5E). Across conditions (somitoids from mTeSR-Plus- and StemFit-cultured iPSCs), these trajectories were largely conserved, with only minor differences in the relative contribution of PSM and paraxial mesoderm cell populations (Figure 3G).

Enhancer-associated profiling in somitoids reveals regulators of human somitogenesis

To gain insight into chromatin-associated regulators that are active during somite formation, we applied P300-based proximity labeling to somitoids at day 5 of differentiation. Mass-spectrometry-based analysis revealed a broad set of P300 proximal transcription factors, co-factors, and chromatin remodelers (Figure 4A; Table S4). Among these were core components of enhancer-associated complexes, including members of the SWI/SNF chromatin remodeling family and the Mediator complex, both of which were also identified in iPSCs (Figures 1I and 4B), Several factors previously linked to somitogenesis were detected, including MESP2 and TBX6, as well as ZEB2, consistent with our prior findings that ZEB2 is essential for the formation of segmented structures during mouse and human somitogenesis (Stelloo et al., 2024). In addition, we identified regulators of key signaling pathways that orchestrate somite formation, including components of the Wnt pathway (e.g., CTNNB1, AXIN1, and LEF1) and the Notch pathway (e.g., RBPJ, NOTCH1, NOTCH2, and NRARP) (Figure 4C). Among these interactors, some proteins appear at enhancers concomitantly with increased expression or protein abundance during the transition from iPSCs to somitoids, such as ZEB2, PYGO1, and TCF15, whereas others, including beta-catenin (CTNNB1), appear to be recruited in an activity-dependent manner (Figures 4D and 4E). These findings highlight the integration of enhancer-associated complexes with developmental signaling networks during early human somitogenesis.

Defective somitoid development following targeted knockout of BPTF, RBPJ, and CITED2

To assess the functional relevance of enhancer-associated regulators in iPSCs and somitoids, we selected BPTF, RBPJ, and CITED2, as these genes are known to be viable when knocked out in iPSCs, enabling analysis of their function during subsequent differentiation (Figures 5A–5C). These proteins were also identified as P300 proximity interactors in mouse gastruloids in our previous study (Stelloo et al., 2024). For BPTF, we obtained one validated knockout clone (Figure 5A). For RBPJ, one heterozygous (cl29) and three homozygous knockout clones were generated (Figure 5B). For CITED2, multiple knockout clones were obtained, and two independent clones were selected for downstream analysis (Figure 5C). SOX2 and OCT3/4 expression is maintained following gene knockouts (Figure S6A). These knockout lines were subsequently used to generate somitoids, assessing the impact of each gene on differentiation potential and segmentation. Somitoid development was monitored by daily imaging, and samples were collected at day 5 for bulk RNA-seq.

BPTF is a chromatin remodeler implicated in transcriptional regulation and embryonic development. In mouse embryos, loss of *Bptf* results in clear developmental defects, including failure to establish the anterior-posterior axis formation, mesoderm formation, and arrested development at E9 (Landry et al., 2008). Similar to the observation in mouse embryos, the BPTF-knockout-derived somitoids exhibited a severe developmental phenotype, characterized by a complete loss of axial elongation and segmentation (Figures 5D and 5E; Figures S6B and S6C). Transcriptional profiling revealed a distinct gene expression profile between BPTF knockouts and wild-type controls (Figures 5F and 5G). Differential expression analysis identified 412 significant genes (Table S5), including key regulators of anterior posterior pattern specification (*HOXA9*, *HEY2*, *HES3*, *HOXC9*, *HES5*, and *HOXC6*) and epithelial organization through altered expression of cell junction components (*CDH6*, *CDH3*, *FLRT3*, *NRXN1*, *PCDHB16*, *CDH12*, *NRXN2*, *PCDHB5*, and *SHANK2*) (Figures 5G and 5H). These molecular changes align with the observed morphological defects in mouse embryos and underscore the possible essential function of BPTF in regulating spatial patterning and tissue architecture during early human development.

We next examined the role of RBPJ, a core transcriptional regulator within the Notch signaling pathway, which is essential for somite boundary formation and segmentation (Oka et al., 1995). Mice lacking RBPJ die before embryonic day E10.5, due to defects in neural and somitic development (Oka et al., 1995). Somitoids derived from the heterozygous RBPJ knockout iPSCs showed normal morphology (Figures 5D and 5E; Figures S6B and S6C) and clustered closely with wild-type samples in transcriptomic space

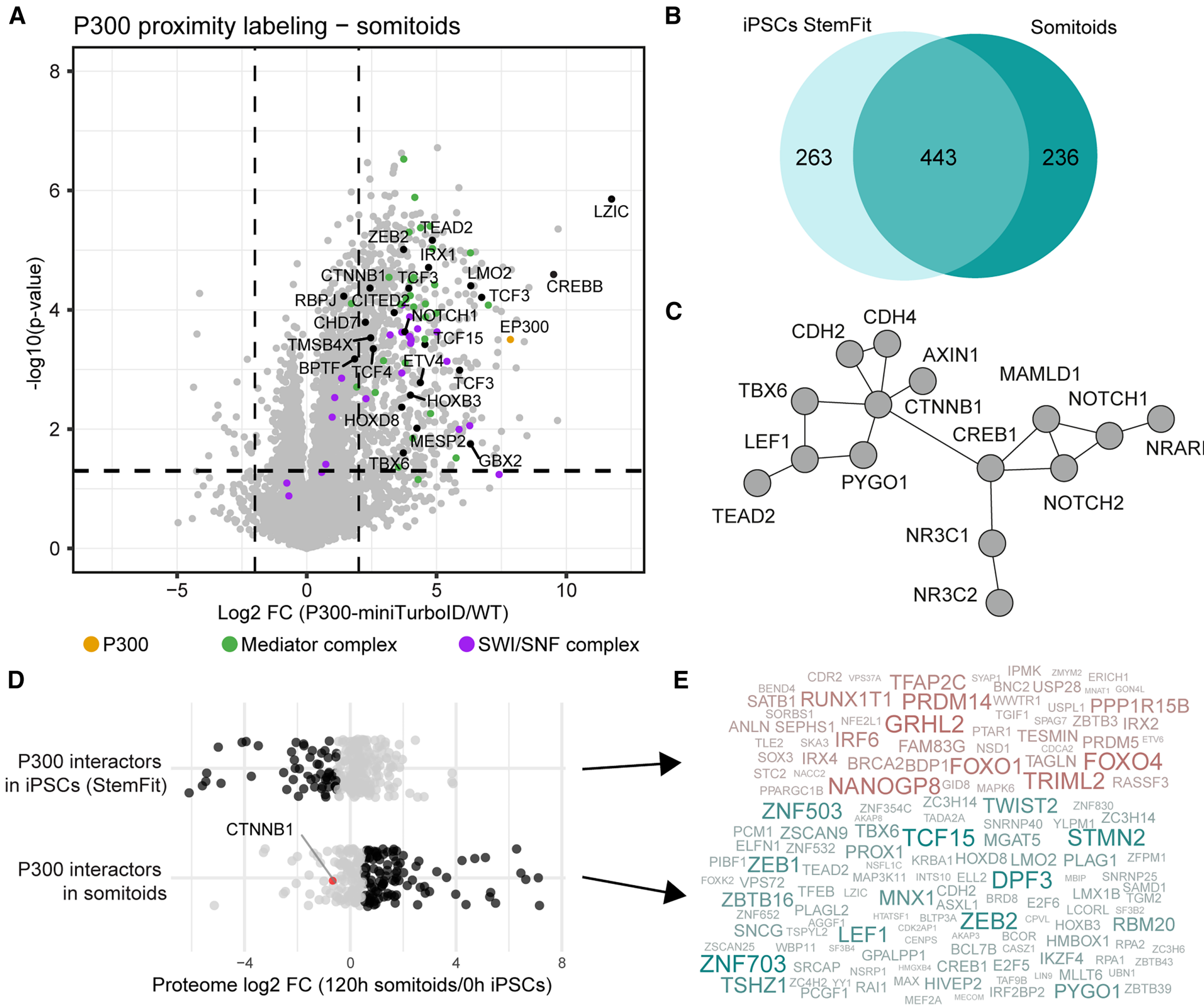

Figure 4. Enhancer-associated profiling in somitoids reveals regulators of human somitogenesis

(A) Volcano plot of streptavidin affinity purifications of biotin-treated P300-miniTurboID-expressing somitoids compared to biotin-treated wild-type somitoids pre-cultured in StemFit medium. Somitoids were cultured under Matrigel-free (suspension) conditions. The plot corresponds to three biological experiments each with one technical replicate. Horizontal dashed lines represent a *p* value threshold of 0.05, and vertical dashed lines mark a log2 FC threshold of 2. Members of the SWI/SNF chromatin remodeling complex are highlighted in purple, members of the Mediator complex are shown in green, and selected proteins are shown in black.

(B) Venn diagram illustrating the overlap of enhancer-interacting proteins between somitoids (p value < 0.05 and $\log_2$ FC > 2) and wild-type iPSCs pre-cultured in StemFit medium (Consensus P300 proximity labeling interactome).

(C) STRING-based network (Szklarczyk et al., 2025) of selected proteins in somitoids, identified through P300 proximity labeling.

(D) P300 interactors derived from the Venn diagram subsets, grouped according to their presence in somitoids or iPSCs. Corresponding protein abundance changes from whole proteome measurements are shown as $\log_2$ fold change values.

(E) Word cloud highlighting p300 proximity interactors with increased protein expression, where font size reflects $\log_2$ fold change from whole proteome data.

(Figure 5F). This suggests that partial loss of RBPJ does not significantly impact somitoid development and gene expression. In contrast, homozygous knockouts displayed a clear developmental phenotype. While axial elongation was preserved, these structures failed to form distinct segments. Transcriptomic profiling identified 144 differentially expressed genes (Table S6), including many transcriptional regulators (Figures 5I and 5J) involved in neurogenesis (e.g., *SOX3*, *SOX21*, *OLIG3*, *NEUROG2*, *IRX3*, *EN2*, *SIM2*, *PAX6*, *RFX4*, *FOXJ1*, and *GRHL3*) and somitogenesis (*MIXL1*, *TBX18*, *HES3*, *HES4*, *HOXB1*, *WNT3A*, *EDN1*, *DMRTA2*, and *PAX7*). In conclusion, RBPJ is partially dispensable, but complete RBPJ loss disrupts segmentation.

CITED2 is a transcriptional co-regulator implicated in embryonic development as evidenced by defects in neural tube, cardiovascular development, and left-right axis

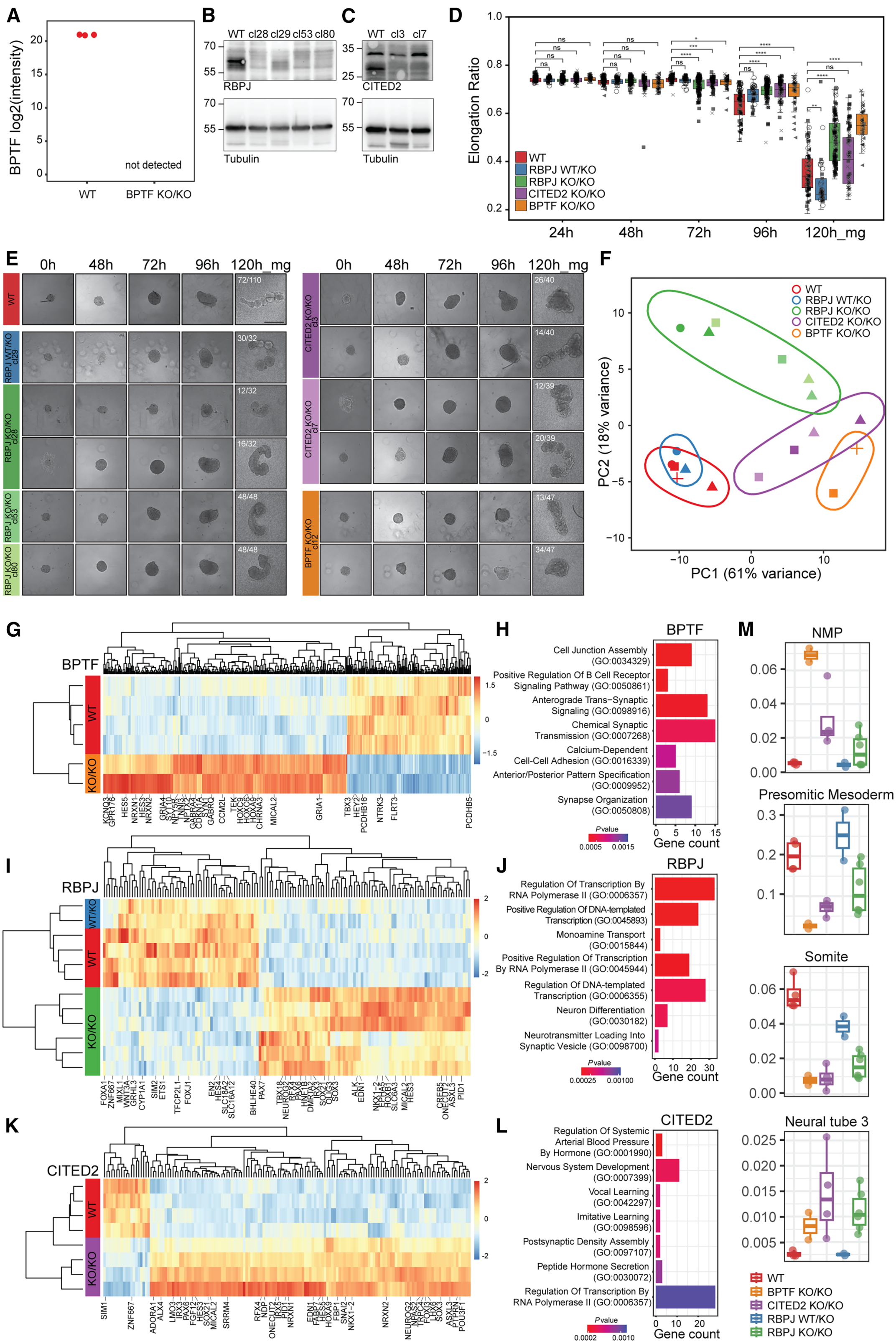

(legend on next page)

specification in knockout mice (Barbera et al., 2002; Lopes Floro et al., 2011; Weninger et al., 2005; Withington et al., 2006). In our somitoid model, CITED2 knockout iPSCs gave rise to structures with largely preserved axial elongation, but segmentation was frequently disrupted or absent (Figures 5D and 5E; Figures S6B and S6C). Bulk RNA-seq identified 150 differentially expressed genes (Figures 5K and 5L; Table S7), including upregulation of genes associated with nervous system development (*SOX3*, *SRRM4*, *NRXN1*, *FABP7*, *ADORA1*, *NRXN2*, *PAX6*, *NDP*, *FGF12*, *NPAS2*, and *HES5*), hinting toward a similar function as observed in mice. To assess whether the segmentation defects observed in the knockout somitoids could be reversed, we performed rescue experiments by reintroducing CITED2 under a doxycycline-inducible promoter into the AAVS1 safe harbor locus. While rescue attempts for RBPJ and BPTF were unsuccessful due to lack of detectable expression and cloning challenges associated with the large (~8 kb) BPTF coding sequence, respectively, CITED2 expression was robustly restored at both the iPSC stage and in somitoids upon doxycycline treatment in both knockout-rescue clones (Figures S6D and S6E). Somitoids were then generated from the wild-type and CITED2 knockout-rescue lines with and without doxycycline throughout the entire differentiation protocol. Induced expression of CITED2 led to partial phenotypic rescue, displaying axial elongation and improved segmentation in some structures (Figures S6F and S6G). Transcriptomic analysis further supported the partial rescue, as CITED2-rescued samples clustered closely with wild-type controls (Figures S6H and S6I). However, the rescue was not complete, suggesting that precise regulation of CITED2 expression, including timing, dosage, and expression across relevant cells may be required for full functional restoration.

To explore shared transcriptional responses underlying the defective segmentation phenotypes observed across all three knockout conditions (BPTF, CITED2, and RBPJ), we performed a comparative analysis. We identified 50 overlapping differentially expressed genes. These genes are enriched for functions in neurogenesis and neural

Figure 5. Defective somitoid development following targeted knockout of BPTF, RBPJ, and CITED2

(A) Generation of BPTF knockout in iPSCs was verified by mass spectrometry. Log_2 transformed LFQ intensities of wild-type (WT) iPSCs and BPTF knockout (KO) iPSCs. Each dot represents a technical replicate.

(B) Western blot of protein extracts from wild-type iPSCs (WT) and RBPJ knockout clones. For RBPJ, clone 29 is heterozygous, while clones 28, 53, and 80 are homozygous knockouts. Blots were incubated with anti-RBPJ and anti-Tubulin antibodies.

(C) Western blot of protein extracts from wild-type iPSCs and CITED2 knockout clones. For CITED2, only homozygous knockouts (clone 3 and clone 7) were analyzed. Blots were incubated with anti-CITED2 and anti-Tubulin antibodies.

(D) Quantification of the elongation ratio of Matrigel-embedded somitoids generated from wild-type cells or RBPJ, BPTF, and CITED2 knockout iPSCs. Each dot represents a single somitoid. Different shapes indicate distinct biological replicates, comprising all clones. More specifically, for BPTF, one knockout clone was analyzed; for CITED2, two knockout clones; and for RBPJ, three homozygous knockout clones and one heterozygous clone. Reported p values are determined with Mann-Whitney-Wilcoxon test two-sided with Bonferroni correction.

(E) Representative images of Matrigel-embedded somitoids derived from wild-type, RBPJ, BPTF, and CITED2 knockout iPSCs over a 5-day time course. Scale bars: 200 μm. The notation *x/x* indicates the number of somitoids displaying the representative phenotype out of the total number analyzed. If the total is not accounted for, remaining samples exhibited mixed or wild-type-like phenotypes.

(F) PCA based on the top 500 most variable expressed genes in Matrigel-embedded somitoids derived from wild-type cells or RBPJ, BPTF, and CITED2 knockout iPSCs. Each dot represents an independent biological replicate comprising all clones. More specifically, for BPTF, one knockout clone was analyzed; for CITED2, two knockout clones; and for RBPJ, three homozygous knockout clones and one heterozygous clone.

(G) Heatmap showing scaled expression of 412 differentially expressed genes (p value < 0.05 and $\log_2$ FC > 2) between BPTF KO somitoids and wild-type somitoids.

(H) Bar plot showing the top enriched Gene Ontology (GO) terms among differentially expressed genes identified in BPTF KO somitoids versus wild-type somitoids.

(I) Heatmap showing scaled expression of 144 differentially expressed genes (p value < 0.05 and $\log_2$ FC > 2) between RBPJ KO somitoids derived from wild-type and RBPJ KO iPSCs.

(J) Bar plot showing the top enriched GO terms among differentially expressed genes identified in RBPJ KO somitoids versus wild-type somitoids.

(K) Heatmap showing scaled expression of 150 differentially expressed genes (p value < 0.05 and $\log_2$ FC > 2) between CITED2 KO somitoids and wild-type somitoids.

(L) Bar plot showing the top enriched GO terms among differentially expressed genes identified in CITED2 KO somitoids versus wild-type somitoids.

(M) Boxplot of cell-type predictions inferred by Scaden in knockout samples, using scRNA-seq from human embryos at gastrulation stage as a reference (GSE155121). Distributions reflect relative cell-type abundances and enable comparison between conditions rather than absolute quantification.

differentiation (*NEUROG2*, *SOX3*, *SOX21*, and *NRXN1*), transcriptional regulation (*HES3*, *IRX3*, *RFX4*, *ONECUT2*, *NKX1-2*, and *ASXL3*), and left-right and axial patterning (*LIX1*, *UNCX*, *IRX3*, and *DNAH11*). The consistent upregulation of neurogenic markers across all knockouts suggests a shift in lineage specification. To further explore this, we calculated a quantitative enrichment score using the differentially expressed genes from each knockout as gene signatures and assessed their representation in individual cells from reference scRNA-seq datasets of human embryos at the gastrulation stage (PCW3) (Figure S7A), complemented by deconvolution analysis using Scaden to infer shifts in cell-type composition (Figures 5M and S7B). From both analyses, we observe that the differentiation state affected differs between perturbations: BPTF loss is associated with retention of progenitor-like (NMP-like) states, whereas CITED2 loss results in an accumulation in early mesoderm (NMP and PSM), with both knockouts showing strongly reduced somite and somitic mesoderm populations. In contrast, RBPJ loss retains somite and somitic mesoderm to a greater extent, although at reduced levels compared to wild type. Across all three perturbations, neural lineage signatures are consistently increased. To assess whether these differentiation impairments arise earlier in development, all three knockout conditions were differentiated into early progenitors of ectoderm, mesoderm, and endoderm. No noticeable differentiation defects were observed (Figure S8). Together, these data support a model in which early mesodermal commitment proceeds largely normally, but subsequent somitic mesoderm specification is perturbed, with increased neural signatures emerging as an indirect consequence of altered mesoderm specification rather than reflecting a true neural fate transition. Overall, our findings demonstrate that somitoids provide a promising and tractable platform for studying the functional consequences of gene knockouts during early human development in a controlled, scalable context.

DISCUSSION

Human embryonic development remains largely inaccessible to investigate due to ethical constraints, particularly during the earliest stages. As a result, *in vitro* models such as somitoids have become essential tools for investigating the cellular and molecular mechanisms underlying early human development. These models offer unique access to processes otherwise inaccessible *in vivo*, yet their reproducibility and robustness can be compromised by technical variables. One critical but often overlooked factor is the choice of maintenance medium during iPSC culture. Our findings demonstrate that commonly used media such as mTeSR Plus, while suitable for general stem cell maintenance, may not support the efficient formation of complex three-dimensional structures like somitoids. This aligns with previous observations by Sanaki-Matsumiya et al., who reported that iPSCs maintained in mTeSR1 medium also fail to generate somitoids efficiently (Sanaki-Matsumiya, 2022). mTeSR1 is very similar to mTeSR Plus except that native FGF2 is stabilized by formulation and has an enhanced buffering capacity compared to mTeSR1 (Ludwig et al., 2006). Importantly, iPSCs cultured in either mTeSR Plus or StemFit are capable of maintaining pluripotency, as well as maintaining core enhancer interactome, suggesting that differences in somitoid formation and efficiency arise from subtle variations in media composition. Although this study focuses on somitoids, similar effects may also apply to other embryo-like models, but this warrants further investigation. While commercially available culture media such as mTeSR1, mTeSR Plus, and StemFit have enabled significant advances in stem cell research, the lack of full disclosure of their formulations poses a challenge to scientific reproducibility and mechanistic insight. Due to intellectual property restrictions, precise formulations remain inaccessible. Given the clear influence of these formulations on differentiation efficiency and developmental outcomes, access to this information would be of high value and could accelerate progress in stem cell research by enabling more standardized and interpretable experimental designs.

The P300 proximity-labeling approach revealed core chromatin-associated networks in stem cells, as well as medium-specific enrichment of distinct protein interactors within the P300 proximity landscape. Importantly, medium-specific enrichment of certain transcription factors in the P300 interactome does not necessarily correlate with expression levels, suggesting post-transcriptional regulation or differential protein recruitment to enhancers. This disconnect between abundance and chromatin association highlights the value of proteomic approaches in uncovering regulatory mechanisms that are invisible to transcriptomic profiling alone. Further functional follow-up will be necessary to determine the biological relevance of these interactions. In somitoids, P300 proximity labeling measurements reflect the combined signal of heterogeneous cell populations within the model and therefore represent an aggregate view of enhancer-associated interactions across cell types rather than cell-type-specific networks. To begin exploring the functional significance of these interactors, we validated the functional relevance of three transcriptional regulators through targeted knockouts and assessed their role in somitoid formation. Our results show that the absence of CITED2 in the human somitoids causes disrupted or absent segmentation. Phenotypes of *Cited2−/−* mouse embryos are also variable, presenting neural tube defects,

exencephaly, and cardiac malformations (Bamforth et al., 2001; Barbera et al., 2002). BPTF knockout in human somitoids shows very drastic effects, with complete loss of elongation. It is reported that the loss of *Bptf* in mouse causes embryonic lethality, with 100% penetrance as early as E7.5 (Landry et al., 2008). Microarray analysis of *Bptf*−/− mouse embryos also revealed upregulation of several HOX genes, similar to the observed effect in human somitoids, where we observed upregulation of *HOXA9*, *HOXC9*, and *HOXC6*. In a similar way, RBPJ in mouse causes embryonic lethality at 10.5 dpc, with reabsorption in some cases starting as early as 8.5 dpc with poorly formed and irregular arranged somites (Oka et al., 1995). Additionally, the mutation of *Notch1* in mouse causes embryo reabsorption by E10 and retarded development, with less somites formed compared to wild-type embryos in the same stage (Ferjentsik et al., 2009). This correlates with our observations in human somitoids, where we observe axial elongation accompanied with a clear absence of segments. Interestingly, heterozygous RBPJ knockout did not exhibit an obvious phenotype *in vitro*, mirroring *in vivo* mouse observations and supporting the fidelity of the somitoid system in modeling early developmental processes (Oka et al., 1995). These findings reinforce the utility of somitoids as a robust and scalable platform for functional genomics in human development.

To build on the insights gained from the targeted knockouts of three transcriptional regulators, a next step would be to perform a focused CRISPR screen in the somitoid model. Such a screen could systematically interrogate a broader set of chromatin-associated factors and transcriptional regulators identified through P300 proximity labeling. This would allow us to move beyond candidate-based approaches and uncover additional genes that modulate segmentation, axial patterning, or mesoderm specification in a context-dependent manner.

While somitoids have previously been used to study somitogenesis, our study expands their application to dissecting gene regulatory networks through integrated proteomic and genetic perturbation approaches. This positions somitoids not only as a model for developmental biology but also as a valuable system for mechanistic studies of transcriptional regulation and disease-relevant gene function.

RESOURCE AVAILABILITY

Lead contact

Further information and requests for resources should be directed to and will be provided by the lead contact, Suzan Stelloo (suzan.stelloo@radboudumc.nl).

Materials availability

Plasmids and cell lines generated in this study are available upon request.

Data and code availability

The sequence data generated in this study have been deposited at the European Genome-phenome Archive (EGA) under the accession number EGAS50000001900. The mass spectrometry proteomics data have been deposited to the ProteomeXchange Consortium via the PRIDE (Perez-Riverol et al., 2025) partner repository with the dataset identifier PXD067912 for P300 proximity labeling in iPSCs, PXD070251 for P300 proximity labeling in somitoids, PXD068407 for whole proteome analysis of BPTF knockout, and PXD079129 for time course proteomics. This paper does not report original code. Any additional information required to reanalyze the data reported in this paper is available from the lead contact upon request.

ACKNOWLEDGMENTS

We thank members of the Vermeulen laboratory for fruitful discussions. We would also like to thank Laura Wingens and Tom van Oorschot for their technical support with cell sorting, Sybren Rinzema for uploading the sequencing data to European Genome-phenome Archive (EGA), the laboratory of Emile van den Akker (Sanquin Research, Amsterdam) for providing the three iPSC lines, and Mariya Moosajee for providing the iPSC line UCLi017-A. In addition, we thank the Radboud Technology Center Microscopy at Radboud University Medical Center for providing access to their microscopy facilities for confocal imaging. The Vermeulen laboratory is part of the Oncode Institute, which is partly funded by the Dutch Cancer Society (KWF) and Oncode Accelerator, a Dutch National Growth Fund project under grant number NGFOP2201. This work was supported by two Pluripotent Stem Cells for Inherited Diseases and Embryonic Research (PSIDER) grants from ZonMw (10250042110004 and 10250052410010) and by two grants funded by the European Commission, European Union's Horizon-2020 Marie Skłodowska Curie EpiSyStem ITN Network (765966), and European Union's Horizon-2021 Marie Skłodowska Curie Chrom_Rare (101073334).

AUTHOR CONTRIBUTIONS

S.S. and M.V. conceived the project. M.M. provided the cell line used in this study. S.S., L.A.L., M.T.A.-V., Y.E.S., and Y.W.C. designed and performed experiments. S.S. and M.T.A.-V. performed sequencing analysis. S.S. and M.J.H. performed the SP3 experiments. S.S., M.T.A.-V., and Y.E.S. wrote the manuscript. All authors reviewed and edited the manuscript.

DECLARATION OF INTERESTS

The authors declare no competing interests.

DECLARATION OF GENERATIVE AI AND AI-ASSISTED TECHNOLOGIES IN THE WRITING PROCESS

During the preparation of this work, the author(s) used Copilot to improve clarity and grammar. The author(s) reviewed and edited the output as needed and take full responsibility for the content of the published article.

STAR★METHODS

Detailed methods are provided in the online version of this paper and include the following:

- KEY RESOURCES TABLE
- EXPERIMENTAL MODEL AND STUDY PARTICIPANT DETAILS
  - Cell culture
  - Generation of P300-miniTurboID knockin cell line
  - Trilineage differentiation
  - Human somitoids
  - Generation of BPTF, RBPJ and CITED2 knockout cell lines
- METHOD DETAILS
  - Western blotting
  - SP3 sample preparation
  - Proximity labeling experiments
  - LC-MS/MS
  - Mass spectrometry data analysis
  - ChIP-seq
  - RNA-seq
  - Immunofluorescence
- QUANTIFICATION AND STATISTICAL ANALYSIS
  - MOrgAna analysis
  - Quantification of lineage marker expression

SUPPLEMENTAL INFORMATION

Supplemental information can be found online at https://doi.org/10.1016/j.stemcr.2026.103023.

Received: November 24, 2025
Revised: July 6, 2026
Accepted: July 6, 2026
Published: August 6, 2026

REFERENCES

Anderson, M.J., Magidson, V., Kageyama, R., and Lewandoski, M. (2020). Fgf4 maintains Hes7 levels critical for normal somite segmentation clock function. eLife *9*, e55608. https://doi.org/10.7554/eLife.55608.

Aulehla, A., Wehrle, C., Brand-Saberi, B., Kemler, R., Gossler, A., Kanzler, B., and Herrmann, B.G. (2003). Wnt3a plays a major role in the segmentation clock controlling somitogenesis. Dev. Cell *4*, 395–406. https://doi.org/10.1016/s1534-5807(03)00055-8.

Bamforth, S.D., Bragança, J., Eloranta, J.J., Murdoch, J.N., Marques, F.I., Kranc, K.R., Farza, H., Henderson, D.J., Hurst, H.C., and Bhattacharya, S. (2001). Cardiac malformations, adrenal agenesis, neural crest defects and exencephaly in mice lacking Cited2, a new Tfap2 co-activator. Nat. Genet. *29*, 469–474. https://doi.org/10.1038/ng768.

Barbera, J.P.M., Rodriguez, T.A., Greene, N.D.E., Weninger, W.J., Simeone, A., Copp, A.J., Beddington, R.S.P., and Dunwoodie, S. (2002). Folic acid prevents exencephaly in Cited2 deficient mice. Hum. Mol. Genet. *11*, 283–293. https://doi.org/10.1093/hmg/11.3.283.

Bernstein, B.E., Stamatoyannopoulos, J.A., Costello, J.F., Ren, B., Milosavljevic, A., Meissner, A., Kellis, M., Marra, M.A., Beaudet, A.L., Ecker, J.R., et al. (2010). The NIH Roadmap Epigenomics Mapping Consortium. Nat. Biotechnol. *28*, 1045–1048. https://doi.org/10.1038/nbt1010-1045.

Coyle, P., Philcox, J.C., Carey, L.C., and Rofe, A.M. (2002). Metallothionein: the multipurpose protein. Cell. Mol. Life Sci. *59*, 627–647. https://doi.org/10.1007/s00018-002-8454-2.

Demichev, V., Messner, C.B., Vernardis, S.I., Lilley, K.S., and Ralser, M. (2020). DIA-NN: neural networks and interference correction enable deep proteome coverage in high throughput. Nat. Methods *17*, 41–44. https://doi.org/10.1038/s41592-019-0638-x.

Dietrich, A., Merotto, L., Pelz, K., Eder, B., Zackl, C., Reinisch, K., Edenhofer, F., Marini, F., Sturm, G., List, M., and Finotello, F. (2026). omnideconv: a unifying framework for using and benchmarking single-cell-informed deconvolution of bulk RNA-seq data. Genome Biol. *27*, 6. https://doi.org/10.1186/s13059-026-03955-w.

Ferjentsik, Z., Hayashi, S., Dale, J.K., Bessho, Y., Herreman, A., De Strooper, B., del Monte, G., de la Pompa, J.L., and Maroto, M. (2009). Notch is a critical component of the mouse somitogenesis oscillator and is essential for the formation of the somites. PLoS Genet. *5*, e1000662. https://doi.org/10.1371/journal.pgen.1000662.

Gafni, O., Weinberger, L., Mansour, A.A., Manor, Y.S., Chomsky, E., Ben-Yosef, D., Kalma, Y., Viukov, S., Maza, I., Zviran, A., et al. (2013). Derivation of novel human ground state naive pluripotent stem cells. Nature *504*, 282–286. https://doi.org/10.1038/nature12745.

Grand, R.S., Burger, L., Gräwe, C., Michael, A.K., Isbel, L., Hess, D., Hoerner, L., Iesmantavicius, V., Durdu, S., Pregnolato, M., et al. (2021). BANP opens chromatin and activates CpG-island-regulated genes. Nature *596*, 133–137. https://doi.org/10.1038/s41586-021-03689-8.

Gritti, N., Lim, J.L., Anlaş, K., Pandya, M., Aalderink, G., Martínez-Ara, G., and Trivedi, V. (2021). MOrgAna: accessible quantitative analysis of organoids with machine learning. Development *148*, dev199611. https://doi.org/10.1242/dev.199611.

Günther, V., Lindert, U., and Schaffner, W. (2012). The taste of heavy metals: gene regulation by MTF-1. Biochim. Biophys. Acta *1823*, 1416–1425. https://doi.org/10.1016/j.bbamcr.2012.01.005.

Hansen, M., Varga, E., Wüst, T., Mellink, C., van der Kevie-Kersemaekers, A.M., von Lindern, M., and van den Akker, E. (2017). Generation and characterization of human iPSC lines SANi001-A and SANi002-A from mobilized peripheral blood derived megakaryoblasts. Stem Cell Res. *25*, 42–45. https://doi.org/10.1016/j.scr.2017.10.002.

Hao, Y., Stuart, T., Kowalski, M.H., et al. (2024). Dictionary learning for integrative, multimodal and scalable single-cell analysis. Nat Biotechnol *42*, 293–304. https://doi.org/10.1038/s41587-023-01767-y.

Jawaid, W. enrichR: Provides an R Interface to ‘Enrichr’. R package version 3.4. https://github.com/wjawaid/enrichr.

Kolde, R. (2025). pheatmap: Pretty Heatmaps. R package version 1.0.13. https://github.com/raivokolde/pheatmap.

Kuilman, T., Velds, A., Kemper, K., Ranzani, M., Bombardelli, L., Hoogstraat, M., Nevedomskaya, E., Xu, G., de Ruiter, J., Lolkema,

M.P., et al. (2015). CopywriteR: DNA copy number detection from off-target sequence data. Genome Biol. *16*, 49. https://doi.org/10.1186/s13059-015-0617-1.

Landry, J., Sharov, A.A., Piao, Y., Sharova, L.V., Xiao, H., Southon, E., Matta, J., Tessarollo, L., Zhang, Y.E., Ko, M.S.H., et al. (2008). Essential role of chromatin remodeling protein Bptf in early mouse embryos and embryonic stem cells. PLoS Genet. *4*, e1000241. https://doi.org/10.1371/journal.pgen.1000241.

Lerdrup, M., and Hansen, K. (2020). User-Friendly and Interactive Analysis of ChIP-Seq Data Using EaSeq. Methods Mol. Biol. *2117*, 35–63. https://doi.org/10.1007/978-1-0716-0301-7_2.

Lopes Floro, K., Artap, S.T., Preis, J.I., Fatkin, D., Chapman, G., Furtado, M.B., Harvey, R.P., Hamada, H., Sparrow, D.B., and Dunwoodie, S.L. (2011). Loss of Cited2 causes congenital heart disease by perturbing left-right patterning of the body axis. Hum. Mol. Genet. *20*, 1097–1110. https://doi.org/10.1093/hmg/ddq554.

Love, M.I., Huber, W., and Anders, S. (2014). Moderated estimation of fold change and dispersion for RNA-seq data with DESeq2. Genome Biol *15*, 550. https://doi.org/10.1186/s13059-014-0550-8.

Ludwig, T.E., Bergendahl, V., Levenstein, M.E., Yu, J., Probasco, M.D., and Thomson, J.A. (2006). Feeder-independent culture of human embryonic stem cells. Nat. Methods *3*, 637–646. https://doi.org/10.1038/nmeth902.

Méjécase, C., Harding, P., Sarkar, H., Eintracht, J., Lima Cunha, D., Toualbi, L., and Moosajee, M. (2020). Generation of two human control iPS cell lines (UCLi016-A and UCLi017-A) from healthy donors with no known ocular conditions. Stem Cell Res. *49*, 102113. https://doi.org/10.1016/j.scr.2020.102113.

Miao, Y., Djeffal, Y., De Simone, A., Zhu, K., Lee, J.G., Lu, Z., Silberfeld, A., Rao, J., Tarazona, O.A., Mongera, A., et al. (2023). Reconstruction and deconstruction of human somitogenesis in vitro. Nature *614*, 500–508. https://doi.org/10.1038/s41586-022-05655-4.

Miao, Y., and Pourquié, O. (2024). Cellular and molecular control of vertebrate somitogenesis. Nat. Rev. Mol. Cell Biol. *25*, 517–533. https://doi.org/10.1038/s41580-024-00709-z.

Nakagawa, M., Taniguchi, Y., Senda, S., Takizawa, N., Ichisaka, T., Asano, K., Morizane, A., Doi, D., Takahashi, J., Nishizawa, M., et al. (2014). A novel efficient feeder-free culture system for the derivation of human induced pluripotent stem cells. Sci. Rep. *4*, 3594. https://doi.org/10.1038/srep03594.

Oka, C., Nakano, T., Wakeham, A., de la Pompa, J.L., Mori, C., Sakai, T., Okazaki, S., Kawaichi, M., Shiota, K., Mak, T.W., and Honjo, T. (1995). Disruption of the mouse RBP-J kappa gene results in early embryonic death. Development *121*, 3291–3301. https://doi.org/10.1242/dev.121.10.3291.

Pedersen, L., Jensen, M.H., and Krishna, S. (2011). Dickkopf1–a new player in modelling the Wnt pathway. PLoS One *6*, e25550. https://doi.org/10.1371/journal.pone.0025550.

Perez-Riverol, Y., Bandla, C., Kundu, D.J., Kamatchinathan, S., Bai, J., Hewapathirana, S., John, N.S., Prakash, A., Walzer, M., Wang, S., and Vizcaíno, J.A. (2025). The PRIDE database at 20 years: 2025 update. Nucleic Acids Res. *53*, D543–D553. https://doi.org/10.1093/nar/gkae1011.

Rappsilber, J., Mann, M., and Ishihama, Y. (2007). Protocol for micro-purification, enrichment, pre-fractionation and storage of peptides for proteomics using StageTips. Nat. Protoc. *2*, 1896–1906. https://doi.org/10.1038/nprot.2007.261.

Ross-Innes, C.S., Stark, R., Teschendorff, A.E., Holmes, K.A., Ali, H.R., Dunning, M.J., Brown, G.D., Gojis, O., Ellis, I.O., Green, A.R., et al. (2012). Differential oestrogen receptor binding is associated with clinical outcome in breast cancer. Nature *481*, 389–393. https://doi.org/10.1038/nature10730.

Sanaki-Matsumiya, M., Matsuda, M., Gritti, N., Nakaki, F., Sharpe, J., Trivedi, V., and Ebisuya, M. (2022). Periodic formation of epithelial somites from human pluripotent stem cells. Nat. Commun. *13*, 2325. https://doi.org/10.1038/s41467-022-29967-1.

Sanaki-Matsumiya, M., and Ebisuya, M. (2022). Human embryonic organoids to recapitulate periodic somitogenesis in vitro. https://doi.org/10.21203/rs.3.pex-1852/v1.

Stelloo, S., Alejo-Vinogradova, M.T., van Gelder, C.A.G.H., Zijlmans, D.W., van Oostrom, M.J., Valverde, J.M., Lamers, L.A., Rus, T., Sobrevals Alcaraz, P., Schäfers, T., et al. (2024). Deciphering lineage specification during early embryogenesis in mouse gastruloids using multilayered proteomics. Cell Stem Cell *31*, 1072–1090.e8. https://doi.org/10.1016/j.stem.2024.04.017.

Stirling, D.R., Swain-Bowden, M.J., Lucas, A.M., Carpenter, A.E., Cimini, B.A., and Goodman, A. (2021). CellProfiler 4: improvements in speed, utility and usability. BMC Bioinf. *22*, 433. https://doi.org/10.1186/s12859-021-04344-9.

Szklarczyk, D., Nastou, K., Koutrouli, M., Kirsch, R., Mehryary, F., Hachilif, R., Hu, D., Peluso, M.E., Huang, Q., Fang, T., et al. (2025). The STRING database in 2025: protein networks with directionality of regulation. Nucleic Acids Res. *53*, D730–D737. https://doi.org/10.1093/nar/gkae1113.

Tyanova, S., Temu, T., Sinitcyn, P., Carlson, A., Hein, M.Y., Geiger, T., Mann, M., and Cox, J. (2016). The Perseus computational platform for comprehensive analysis of (prote)omics data. Nat. Methods *13*, 731–740. https://doi.org/10.1038/nmeth.3901.

van den Brink, S.C., Alemany, A., van Batenburg, V., Moris, N., Blotenburg, M., Vivié, J., Baillie-Johnson, P., Nichols, J., Sonnen, K.F., Martinez Arias, A., and van Oudenaarden, A. (2020). Single-cell and spatial transcriptomics reveal somitogenesis in gastruloids. Nature *582*, 405–409. https://doi.org/10.1038/s41586-020-2024-3.

van der Sande, M., Frölich, S., Schäfers, T., Smits, J.G.A., Snabel, R.R., Rinzema, S., and van Heeringen, S.J. (2023). Seq2science: an end-to-end workflow for functional genomics analysis. PeerJ *11*, e16380. https://doi.org/10.7717/peerj.16380.

Veenvliet, J.V., Bolondi, A., Kretzmer, H., Haut, L., Scholze-Wittler, M., Schifferl, D., Koch, F., Guignard, L., Kumar, A.S., Pustet, M., et al. (2020). Mouse embryonic stem cells self-organize into trunk-like structures with neural tube and somites. Science *370*, eaba4937. https://doi.org/10.1126/science.aba4937.

Weninger, W.J., Lopes Floro, K., Bennett, M.B., Withington, S.L., Preis, J.I., Barbera, J.P.M., Mohun, T.J., and Dunwoodie, S.L. (2005). Cited2 is required both for heart morphogenesis and establishment of the left-right axis in mouse development.

Development *132*, 1337–1348. https://doi.org/10.1242/dev.01696.

Wickham, H. (2016). ggplot2: Elegant Graphics for Data Analysis (New York: Springer-Verlag).

Withington, S.L., Scott, A.N., Saunders, D.N., Lopes Floro, K., Preis, J.I., Michalicek, J., Maclean, K., Sparrow, D.B., Barbera, J.P.M., and Dunwoodie, S.L. (2006). Loss of Cited2 affects trophoblast formation and vascularization of the mouse placenta. Dev. Biol. *294*, 67–82. https://doi.org/10.1016/j.ydbio.2006.02.025.

Yamanaka, Y., Hamidi, S., Yoshioka-Kobayashi, K., Munira, S., Sunadome, K., Zhang, Y., Kurokawa, Y., Ericsson, R., Mieda, A., Thompson, J.L., et al. (2023). Reconstituting human somitogenesis in vitro. Nature *614*, 509–520. https://doi.org/10.1038/s41586-022-05649-2.

Yu, G., Wang, L.G., Han, Y., and He, Q.Y. (2012). clusterProfiler: an R package for comparing biological themes among gene clusters. OMICS *16*, 284–287. https://doi.org/10.1089/omi.2011.0118.

Zeng, B., Liu, Z., Lu, Y., Zhong, S., Qin, S., Huang, L., Zeng, Y., Li, Z., Dong, H., Shi, Y., et al. (2023). The single-cell and spatial transcriptional landscape of human gastrulation and early brain development. Cell Stem Cell *30*, 851–866.e7. https://doi.org/10.1016/j.stem.2023.04.016.

Zhu, A., Ibrahim, J.G., and Love, M.I. (2019). Heavy-tailed prior distributions for sequence count data: removing the noise and preserving large differences. Bioinformatics *35*, 2084–2092. https://doi.org/10.1093/bioinformatics/bty895.

STAR★METHODS

KEY RESOURCES TABLE

REAGENT or RESOURCE	SOURCE	IDENTIFIER
**Antibodies**		
Polyclonal Rabbit Anti-Mouse Immunoglobulins/HRP	Agilent	P0260; RRID:AB_2636929
Polyclonal Swine Anti-Rabbit Immunoglobulins/HRP	Agilent	P0399; RRID:AB_2617141
Streptavidin, horseradish peroxidase conjugate	Invitrogen	S911
anti-RBPJ	Santa Cruz Biotechnology	sc-271128; RRID:AB_10610612
anti-CITED2	Abcam	Ab108345; RRID:AB_10864614
anti-V5	Invitrogen	46-1157; RRID:AB_2792973
anti-P300	Santa Cruz Biotechnology	sc-585; RRID:AB_2231120
anti-Tubulin	Sigma	T6074; RRID:AB_477582
Avidin-FITC	Invitrogen	A821
anti-OCT3/4	Santa Cruz Biotechnology	sc-5279; RRID:AB_628051
anti-SOX2	eBioscience	14-9811-80; RRID:AB_11219070
anti-Nestin	Invitrogen	MA1-110 (10C2); RRID:AB_2536821
anti-PAX6	Biolegend	PRB-278P; RRID:AB_291612
anti-SOX17	R&D Systems	AF1924; RRID:AB_355060
anti-CXCR4	Abcam	ab124824; RRID:AB_10975635
anti-FOXA2	R&D Systems	AF2400; RRID:AB_2294104
anti-MEOX1	OriGene	TA804716
anti-Brachyury	R&D Systems	AF2085; RRID:AB_2200235
anti-ZO-1	Invitrogen	33-9100; RRID:AB_2533147
Alexa Fluor 647-conjugated	Invitrogen	A-31571; RRID:AB_162542
Alexa Fluor 647-conjugated	Invitrogen	A-32795; RRID:AB_2762835
Alexa Fluor 647-conjugated	Invitrogen	A-21245; RRID:AB_2535813
Alexa Fluor 488-conjugated	Invitrogen	A-21208; RRID:AB_2535794
Alexa Fluor 488-conjugated	Invitrogen	A-21202; RRID:AB_141607
Alexa Fluor 488-conjugated	Invitrogen	A-11006; RRID:AB_2534074
Alexa Fluor 594-conjugated	Invitrogen	A-11058; RRID:AB_2534105

(*Continued on next page*)

Continued

REAGENT or RESOURCE	SOURCE	IDENTIFIER
**Chemicals, peptides, and recombinant proteins**		
mTeSR Plus	STEMCELL Technologies	100–0276
StemFit Basic04CT	Ajinomoto	Basic04CT
NDiff227	Takara	Y40002
Vitronectin XF	STEMCELL Technologies	100–0763
Penicillin-Streptomycin	GIBCO	15140122
ReLeSR	STEMCELL Technologies	100–0484
CryoStor CS10	STEMCELL Technologies	100–1061
Y-27632 ROCK inhibitor	Sigma	Y0503
SB431542	STEMCELL Technologies	72232
CHIR99021	Axon	1386
DMH1	STEMCELL Technologies	73632
bFGF	PeproTech	AF-100-18B
Matrigel	Corning	356231
Disuccinimidyl glutarate	Thermo Scientific	11836794
Methanol-free formaldehyde	Thermo Scientific	10751395
Dynabeads Protein A	Invitrogen	10008D
Dynabeads Protein G	Invitrogen	10009D
Glycerol	MP Biomedicals	193996
IGEPAL CA-630	Sigma-Aldrich	I8896
Urea	Sigma	U5378
Sera-Mag™ SpeedBeads	Sigma	GE65152105050250
Sera-Mag™ Carboxylate-Modified Magnetic Particles	Sigma	GE45152105050250
Acetone	Sigma	270725
Acetonitrile	Biosolve	1204101BS
Trypsin	Promega	V5280
Trifluoroacetic acid (TFA)	Biosolve	202341
D-biotin	Life Technologies	B20656
PBS	Thermo Fisher Scientific	14190094
cOmplete Protease Inhibitor Cocktail (CPI)	Roche	4693116001
Streptavidin Sepharose High-Performance beads	Cytiva	15511301
Ethidium bromide	Sigma	E1510
Formaldehyde	Sigma	F8775

(*Continued on next page*)

Continued

REAGENT or RESOURCE	SOURCE	IDENTIFIER
Triton X-100	Sigma-Aldrich	X100
Fluoromount G mounting medium with DAPI	Invitrogen	00-4959-52
**Critical commercial assays**		
P3 Primary Cell 4D-Nucleofector X Kit L	Lonza	V4XP-3024
STEMdiff Trilineage Differentiation Kit	STEMCELL Technologies	05230
Pierce BCA Protein Assay Kit	Thermo Scientific	23225
KAPA HyperPrep Kit	KAPA Biosystems	07962363001
KAPA HyperPrep Kit with RiboErase	KAPA Biosystems	08105952001 and 07962274001
Qubit 1X dsDNA HS Assay Kit	Invitrogen	Q33231
Quick-RNA MicroPrep Kit	Zymo Research	R1051
**Deposited data**		
RNA-seq	This study	EGAS50000001900
ChIP-seq	This study	EGAS50000001900
P300 proximity labeling in undifferentiated iPSCs	This study	PXD067912
P300 proximity labeling in human somitoids	This study	PXD070251
Time course proteomics of human somitoids	This study	PXD079129
Whole proteome analysis BPTF knock-out iPSCs	This study	PXD068407
Publicly available datasets for ChIP-seq analysis	Bernstein et al. (2010)	GSE17312
Publicly available datasets for ChIP-seq analysis	Gafni et al. (2013)	GSE52617
Publicly available single-cell RNA-seq data from human embryos at gastrulation stage	Zeng et al. (2023)	GSE155121
**Experimental models: Cell lines**		
iPSC EBL.cl3 iPSC (female)	Sanquin	N/A
iPSC SANi001-A (male)	Hansen et al. (2017)	RRID:CVCL_QY82
iPSC SANi002-A (female)	Hansen et al. (2017)	RRID:CVCL_QY83
iPSC UCLi017-A (male)	Méjécase et al. (2020)	RRID:CVCL_A4DL
**Oligonucleotides**		
gRNA to linearize donor plasmid 5′-GTGTTGTGGACTGCGGCGGT-3′	Grand et al. (2021)	N/A
p300 sgRNA targeting 5′-TTTGCTCCCAAAATACTACA-3′	This paper	N/A
BPTF sgRNA targeting 5′-TACGAGGTACTGCGGAACTT-3′ and 5′-GCAACACAGTCAGTCACACC-3′	This paper	N/A

(*Continued on next page*)

Continued

REAGENT or RESOURCE	SOURCE	IDENTIFIER
RBPJ sgRNA targeting 5′-TTTGCAGTATGCATTGCCTC-3′ and 5′-TGGCATGGCACTCCCAAGAT-3′	This paper	N/A
CITED2 sgRNA targeting 5′-GTGTATGTGCTCGCCCATTA-3′ and 5′-GTTCGGGCAGCTCCTTGATG-3′	This paper	N/A
PCR primers	Table S8	N/A
**Recombinant DNA**		
pU6-(BbsI)_CBh-Cas9-T2A-mCherry	Addgene	#64324
pSpCas9(BB)-2A-Puro (PX459)	Addgene	#48139
**Software and algorithms**		
ZEN Microscopy Software (v3.6, Blue Edition)	Zeiss	N/A
seq2science pipeline (v0.7.1)	van der Sande et al. (2023)	https://vanheeringen-lab.github.io/seq2science/
Diffbind (v3.14.0)	Ross-Innes et al. (2012)	https://github.com/hnthirima/DiffBind
CopywriteR (v1.0.2)	Kuilman et al. (2015)	https://github.com/PeeperLab/CopywriteR
EaSeq (v1.111)	Lerdrup and Hansen (2020)	N/A
DESeq2 (v1.44.0)	Love et al., 2014	https://github.com/thelovelab/DESeq2
pheatmap (v1.0.13)	Kolde, 2025	https://github.com/raivokolde/pheatmap
ggplot2 (v4.4.1)	Wickham (2016)	https://github.com/tidyverse/ggplot2
ClusterProfiler (v4.12.0)	Yu et al. (2012)	https://guangchuangyu.github.io/software/clusterProfiler/
enrichR (v3.4)	Jawaid, 2025	https://github.com/guokai8/Enrichr/
Seurat Rpackage (v5.3.1)	Hao et al., 2024	https://github.com/satijalab/seurat
DIA-NN software (v1.9.2)	Demichev et al. (2020)	https://github.com/vdemichev/diann
Perseus (v1.6.8.0)	Tyanova et al. (2016)	https://github.com/framesurge/perseus
DEP (v1.7.1)	N/A	https://github.com/arnesmits/DEP
omnideconv (v0.1.1)	Dietrich et al. (2026)	https://github.com/omnideconv/omnideconv
MOrgAna (v0.1.1)	Gritti et al. (2021)	https://github.com/LabTrivedi/MOrgAna
CellProfiler (v4.2.8)	Stirling et al. (2021)	https://cellprofiler.org/
**Other**		
μ-Slide 8 Well	IBIDI	80826
U-bottom 96-well plates	Greiner Bio-one	650970
96-well PhenoPlate	Revvity	6055302
Amersham Protran Premium 0.45 NC nitrocellulose Western blotting membranes	Cytiva	GE10600002
NEXTflex DNA barcodes	IDT Technologies	N/A

EXPERIMENTAL MODEL AND STUDY PARTICIPANT DETAILS

Cell culture

Human iPSCs (wild-type EBL.cl3 iPSCs (from Sanquin, used in the main figures of this study), SANi001-A (Hansen et al., 2017), SANi002-A (Hansen et al., 2017) and UCLi017-A (Mejecase et al., 2020)) were cultured on vitronectin XF (STEMCELL technologies, 100-0763) coated plates with either mTeSR Plus (STEMCELL Technologies) or StemFit Basic04CT medium (Ajinomoto) supplemented with 50U/mL penicillin-streptomycin (Gibco). For wild-type EBL.cl3 iPSCs, UCLi017-A, SANi001-A and SANi002-A short tandem repeat (STR) profiling was performed by Eurofins Genomics and the genomic DNA copy number profile was obtained from ChIP-seq input samples (Figure S9). Cells were clump passaged every 3 to 5 days using ReLeSR (STEMCELL Technologies) in a ratio of 1:2 to 1:5. For cryopreservation, cells were dissociated with ReLeSR and resuspended in 500 μl of CryoStor (STEMCELL Technologies), before the start of the project a large batch of early passage cells was stored, and fresh cells were taken in culture regularly. Cells were routinely tested for mycoplasma contamination.

Generation of P300-miniTurboID knockin cell line

To endogenously tag the C-terminus of P300 with miniTurboID, we employed a homology-directed repair strategy. A donor sequence was synthesized by Twist Bioscience, comprising a 498 bp left homology arm, a flexible linker, miniTurboID, a V5 epitope tag, a T2A-puromycin resistance cassette, and a 359 bp right homology arm. To enable intracellular linearization of the donor construct, the homology arms were flanked by a guide RNA target site (GTGTTGTGGACTGCGGCGGTCGG) (Grand et al., 2021). The resulting sequence was cloned into a homology donor plasmid using ClaI and MluI sites. For targeted insertion at the *EP300* genomic locus, we used the guide RNA 5′-TTTGCTCCCAAAATACTACA-3′. The two guide RNAs were cloned into pU6-(BbsI)_CBh-Cas9-T2A-mCherry plasmid (Addgene, #64324). Cells were transfected using an Amaxa Nucleofector II device (program DS-137) and the Lonza Nucleofector Kit P3, along with the donor plasmid, target guide RNA, and plasmid targeting guide RNA. Following transfection, the medium was supplemented with 10 μM Y-27632 ROCK inhibitor (Sigma) for 24 hours. Five days later, cells were cultured in puromycin-containing medium (0.25 μg/mL), and individual colonies were picked.

Trilineage differentiation

Trilineage differentiation was performed using the STEMdiff Trilineage Differentiation kit (STEMCELL Technologies) according to the manufacturer's instructions. Briefly, iPSC were seeded onto Matrigel-coated μ-Slide 8 Well (IBIDI) in maintenance medium (mTeSR Plus) supplemented with 10 μM Y-27632 ROCK inhibitor. Cells were seeded at 200 000 cells/cm^2 for ectoderm and mesoderm differentiation and 50 000 cells/cm^2 for mesoderm differentiation. Twenty-four hours after seeding, the maintenance medium was replaced with STEMdiff Trilineage Ectoderm, Mesoderm or Endoderm medium. Cells were cultured with daily medium changes for 5 days (mesoderm and endoderm) and 7 days (ectoderm). Afterwards, cells were fixed and assessed for lineage-specific markers by immunofluorescence.

Human somitoids

Human somitoids were generated as previously described (Sanaki-Matsumiya et al., 2022; Stelloo et al., 2024). Briefly, iPSCs were sorted using a BD FACSMelody cell sorter into U-bottom 96-well plates (Greiner Bio-one, 650970) at a density of 350 cells per well in 50 μL of somitoid induction medium (NDiff227 (Takara) supplemented with 10 μM SB431542 (STEMCELL Technologies), 10 μM CHIR99021 (Axon), 2 μM DMH1 (STEMCELL Technologies), 20 ng/mL bFGF (PeproTech), and 10 μM Y-27632 ROCK inhibitor). Following sorting, plates were centrifuged at 150 × g for 2 minutes. For SANi001-A, SANi002-A, and UCLi017 lines, a higher initial cell number was required to obtain good size aggregates, and a CHIR99021 concentration of 7 μM. In cases where cell survival after FACS was poor, aggregates were manually counted to ensure aggregate formation. At 24 hours post-sorting, 150 μL of somitoid medium without ROCK inhibitor was added to each well. The medium was subsequently refreshed with NDiff227 at 48 and 72 hours. At 96 hours, medium was replaced with NDiff227 supplemented with 10% Matrigel (Corning). For somitoids used in p300 proximity labeling, Matrigel was omitted because it interferes with downstream proteomic analysis. Although the absence of Matrigel resulted in structures that were not segmented morphologically, RNA-seq confirmed that their transcriptional profiles were comparable to segmented somitoids (Sanaki-Matsumiya et al., 2022). Brightfield images were acquired daily using a Zeiss Axiophot microscope (10× objective) equipped with an Axiocam camera. Zen imaging software was used to capture images.

Generation of BPTF, RBPJ and CITED2 knockout cell lines

Knockout of the selected targets in iPSCs was achieved by transfecting pairs of guide RNAs targeting either BPTF (5′-TAC-GAGGTACTGCGGAACTT-3′ and 5′-GCAACACAGTCAGTCACACC-3′), RBPJ (5′-TTTGCAGTATGCATTGCCTC-3′ and 5′-TGGCATGGCACTCCCAAGAT-3′), or CITED2 (5′-GTGTATGTGCTCGCCCATTA-3′ and 5′-GTTCGGGCAGCTCCTT-GATG-3′). For each pair, one guide RNA was cloned into the pSpCas9(BB)-2A-Puro (PX459) plasmid (Addgene, #48139) containing a puromycin resistance cassette and the other into the pU6-(BbsI)_CBh-Cas9-T2A-mCherry plasmid (Addgene, #64324) containing a mCherry cassette. Transfection of the guide RNA expression vectors was performed using an Amaxa Nucleofector II device with program DS-137 and the Lonza Nucleofector Kit P3. Following transfection, the culture medium was supplemented with 10 μM Y-27632 ROCK inhibitor for 24 hours. At 48 hours post-transfection, mCherry-positive cells were sorted using a BD FACSMelody cell sorter and treated with 0.25 μg/mL puromycin and 10 μM Y-27632 ROCK inhibitor for 24 hours. Single cell-derived clones were isolated and screened using primers listed in Table S8.

METHOD DETAILS

Western blotting

For western blot analysis of the knockout cell lines, cells were lysed in Laemmli buffer (120 mM Tris, 20% glycerol, 4% SDS and CPI). Lysates were sonicated for 6–9 cycles (30 seconds on, 30 seconds off) using a Diagenode Bioruptor Pico. Protein concentrations were determined using the BCA protein assay (Thermo Scientific, 23225). Prior to loading, samples were supplemented with 100 mM DTT and bromophenol blue, then boiled for 10 minutes at 95°C. Proteins were separated by SDS-PAGE and transferred onto Amersham™ Protran 0.45 μm nitrocellulose membranes. Membranes were blocked for 1 hour in 5% milk or BSA prepared in PBS-T and incubated for 1 hour at room temperature or overnight at 4°C with primary antibodies, followed by incubation with appropriate secondary HRP antibodies. Images were acquired using the LAS4000 imager (GE Healthcare). The following primary antibodies were used: RBPJ (1:1000), CITED2 (1:1000), V5 (1:1000), P300 (1:1000) and Tubulin (1:1000). Streptavidin-HRP was used to detect biotinylated proteins (Invitrogen, S911).

SP3 sample preparation

For BPTF knockout validation, technical replicates were performed, whereas time course analyses were carried out using two or three independent biological replicates. Each biological replicate consists of 96 aggregates at the 48h time point and 48 somitoid structures at the 72h, 96h and 120h timepoints. Cells and somitoids were washed with PBS and collected by centrifugation. Pellets were resuspended in lysis buffer (150 mM NaCl, 50 mM Tris pH 8.0, 1 mM EDTA, 10% glycerol, 1% NP-40, 1 mM DTT, and CPI) and sonicated (5 cycles, 30 on, 30 off) with a Bioruptor Pico (Diagenode). Protein concentration was determined with a BCA protein assay, and 20 μg of protein for each replicate was used whenever sufficient material was available. For Matrigel-only samples, 20 μg Matrigel was used as input. Prior to SP3 method, protein was precipitated with 10 volumes of acetone and pellets were dissolved in a Urea buffer (8M urea, 100 mM Tris-HCl pH 8.0, 10 mM DTT) followed by shaking at 1250 rpm at room temperature for 20 minutes. Following, iodoacetamide was added at a final concentration of 50 mM and incubated for 10 minutes at room temperature in the dark. For SP3 method, Sera-Mag™ SpeedBeads (Sigma, GE65152105050250) and Sera-Mag™ Carboxylate-Modified Magnetic Particles (Sigma, GE45152105050250) were mixed in a 1:1 ratio. Prewashed SP3 beads were added to the lysate, followed by the addition of 1.4 volumes of acetonitrile, and incubated at room temperature for 20 minutes. Beads were immobilized using a magnetic rack and washed with acetone (Sigma, 270725), twice with 70% ethanol and once with acetonitrile (Biosolve, 1204101BS). After air-drying, beads were resuspended in 50 μL of 50 mM ammonium bicarbonate (ABC) and supplemented with 0.1 μg trypsin (Promega, V5280). Proteins were digested for two hours while shaking at 37°C, after which the supernatants were collected. Another 50 μL of 50 mM ABC was added to the beads, incubated for 5 minutes in a thermoshaker and supernatants were combined with the first elution. An additional 0.1 μg trypsin was added and digestion was continued overnight at 37°C. The following day, peptides were acidified by adding 10 μL of 10% v/v TFA (Biosolve, 202341) and subsequently purified on C18 Stagetips.

Proximity labeling experiments

iPSCs and somitoids were treated with 50 μM biotin (Life Technologies, B20656) for 1 hour at 37°C. Following treatment, iPSCs were harvested by scraping on ice and pelleted by centrifugation for 5 minutes at 300 × g at 4°C. Somitoids were collected by centrifugation using custom made collection plates for 5 minutes at 300 × g at 4°C. Pelleted samples were washed twice with cold PBS (Thermo Fisher Scientific, 14190094) and resuspended in five volumes of cell lysis buffer (150 mM NaCl, 50 mM Tris pH 8.0, 1 mM EDTA, 10% glycerol, 1% NP-40, 1 mM DTT, and complete protease inhibitors

(CPI)). Lysates were rotated for 1-2 hours at 4°C and centrifuged for 30 minutes at 20,000 × g at 4°C. The supernatant was collected and protein concentration was measured using BCA protein assay (Thermo Scientific, 23225). Protein lysates from iPSCs were split into three replicates per sample, and two biological replicate experiments were performed. For somitoids, five 96-well plates treated without biotin and five 96-well plates treated with biotin were harvested as one replicate; this experiment was performed in three biological replicates to generate triplicates for mass spectrometry. For each pulldown, whole cell extract was incubated with 7.5 µL (15 µL slurry) pre-washed Streptavidin Sepharose High- Performance beads (Cytiva, 15511301) and 2 µL of 10mg/ml ethidium bromide (Sigma, E1510) in a total volume of 400 µL for 2 hours in a rotation wheel at 4°C. For all washing steps, beads were pelleted by centrifugation at 2,000 × *g* for 2 minutes at 4°C. The beads were washed twice with RIPA buffer (25 mM Tris, 7.5 pH, 150 mM NaCl, 1% NP-40, 1% sodium deoxycholate, 0.1% SDS, 0.5 mM DTT), twice with PBS containing 1% NP40 and twice with PBS. For mass spectrometry analysis, beads were processed as previously described (Stelloo et al., 2024). In brief, beads were resuspended in 50 µL of elution buffer (2 M urea, 100 mM Tris pH 8.0, 10 mM DTT) and incubated for 20 minutes at room temperature in a thermo shaker at 1,250 rpm. Following this, iodoacetamide (50 mM) was added to the beads and incubated for an additional 10 minutes in the dark while shaking at 1,250 rpm. Next, 0.25 µg trypsin (Promega, V5113) was added, and incubated for 2 hours at room temperature in a thermo shaker at 1,250 rpm. The supernatant was transferred to new tubes and further digested overnight at room temperature with an additional 0.1 µg of trypsin. Peptide solutions were acidified with 1% trifluoroacetic acid and desalted using homemade C18 StageTips (Rappsilber et al., 2007). For western blot analysis of the streptavidin pulldowns, beads were resuspended in Laemmli buffer (120 mM Tris, 20% glycerol, 4% SDS, 100 mM DTT, and CPI) and boiled for 10 minutes at 95°C.

LC-MS/MS

Digested peptides were eluted from homemade C18 StageTips using buffer B (0.1% formic acid, 80% acetonitrile) and reconstituted in buffer A (0.1% formic acid) to a final volume of 20 µL for SP3 samples and 12 µL for pulldown samples. Eluted peptides (1 µL for knockout validation samples, 1 µL for time-course SP3 samples and 3 µL for pulldown samples) were loaded onto a Vanquish Neo nano-LC system (Thermo Scientific) coupled to an Orbitrap Astral mass spectrometer (Thermo Scientific) operating in data-independent acquisition (DIA) mode. Peptides were separated on a 25 cm column (IonOpticks , AUR3-25075C18XT) using either a 42- minute (SP3 samples) or a 24-minute (pulldown samples) active gradient with a flow rate 400 nL/min increasing to 500 nL/min during the wash out. MS1 scans were acquired from 380 to 980 m/z at 240,000 resolution. RF lens (%) was set to 40 and the normalized AGC target was set to 500% with a maximum injection time of 5ms. For DIA MS2, a normalized HCD collision energy of 25% was applied to a 380-980 m/z precursor range using non-overlapping isolation windows of 2Th, with window placement optimization turned on. Scans were acquired in the Astral analyzer over a 150-2000 m/z range, with the normalized AGC target set to 500% with a maximum injection time of 3 ms.

Mass spectrometry data analysis

Raw mass spectrometry spectra were processed using DIA-NN software (version 1.9.2) (Demichev et al., 2020) with the library-free search method and match between runs enabled. We used a FASTA file of the *Homo sapiens* proteome, downloaded from UniProt in June 2024 (UniProtKB proteome ID: UP000005640). Additionally, a FASTA file containing common contaminants from the cRAP database (April 2019 release) was included as a contaminant list. Trypsin was specified as the proteolytic enzyme, allowing for up to two missed cleavages. Cysteine carbamidomethylation, methionine oxidation, N-terminal methionine excision, and N-terminal acetylation were included as modifications, with a maximum of five variable modifications per peptide. Protein groups with more than two unique peptides and quantified in all replicates of at least one experimental group were retained for downstream analysis. Missing values were imputed for statistical analysis using the "Replace missing values from normal distribution" function with default settings in Perseus (version 1.6.8.0) (Tyanova et al., 2016). For time course analyses, data normalization was performed using variance stabilizing normalization (VSN) within the DEP R package. Missing values were imputed by sampling from a left-shifted Gaussian distribution relative to the observed data distribution. Data visualization was performed using ggplot2 in R (v4.4.1) (Wickham, 2016). Gene ontology enrichment analysis was performed on protein clusters derived from temporal expression patterns using the compareCluster function from the clusterProfiler R package (v4.12.0) (Yu et al., 2012). Cell type deconvolution was performed using the R package omnideconv (Dietrich et al., 2026), employing the deep learning–based method Scaden. The model was trained on annotated single-cell RNA-seq data from human embryos at gastrulation stage (Zeng et al., 2023). Scaden deconvolution provides model-based estimates of the relative contribution of each reference cell type to the bulk expression profile. These scores can be interpreted as approximate cell-type fractions and are

most informative when comparing relative differences between conditions, rather than absolute proportions. For P300 pulldown experiments, consensus lists containing the significant proteins (p value < 0.05 and a $\log_2$ FC > 2 in at least one biological replicate, and a $\log_2$ FC > 1 in the other replicate) can be found in Table S1 (StemFit) and Table S2 (mTeSR Plus). Table S3 contains the significant proteins (p value < 0.05 and a $\log_2$ FC > 2) identified in somitoids.

ChIP-seq

In brief, iPSCs (32 x 10^6 cells per replicate) were crosslinked in solution A with 2 mM disuccinimidyl glutarate (Thermo Scientific) for 25 minutes, followed by 1% methanol-free formaldehyde (Thermo Scientific) for 10 minutes, and quenched with glycine. Chromatin extracts were sonicated for 3 cycles (30 seconds on, 30 seconds off) using a Diagenode Bioruptor Pico. For each ChIP, 4 μg of anti-V5 antibody (Invitrogen) was pre-conjugated to 40 μL of Protein A/G magnetic beads (Invitrogen). Immunoprecipitated DNA was processed for library preparation using the KAPA HyperPrep Kit (KAPA Biosystems), barcoded with NEXTflex DNA barcodes (IDT Technologies), and paired-end sequenced on an Illumina NextSeq500. Sequence reads were processed using the seq2science pipeline (v0.7.1) (van der Sande et al., 2023). Briefly, paired-end reads were trimmed with fastp (v0.20.1, default settings) and aligned to the *Homo sapiens* genome assembly GRCh38.p13 using bwa-mem2 (v2.2.1, with '-M' option). Aligned reads were filtered based on mapping quality (MAPQ $\geq$ 30), and duplicate reads and those mapping to ENCODE blacklisted regions were removed. Peaks were called using MACS2 (v2.2.7) with default settings in BAMPE mode. Only peaks present in both biological replicates and located on canonical chromosomes (chr1–chr22 and chrX) were used for downstream analysis. Genome browser snapshots were generated using EaSeq (v1.111) (Lerdrup and Hansen, 2020). Motif analysis was performed using the SeqPos motif tool with default settings, and genomic region enrichment analysis was conducted with CEAS (http://cistrome.org/ap/). Publicly available datasets used in this study were obtained from GEO (GSE17312 (Bernstein et al., 2010) and GSE52617 (Gafni et al., 2013)) and re-analyzed using the seq2science pipeline. Diffbind (v3.14.0) R package was used to make PCA plots from ChIP-seq data (Ross-Innes et al., 2012). Copy number profiles were derived from either ChIP samples or input samples that were processed similarly to ChIP samples, except these samples were sequenced on an Illumina NextSeq 2000 platform. Copy number analysis was performed using the CopywriteR R package (v1.0.2) (Kuilman et al., 2015).

RNA-seq

RNA was extracted using the Quick-RNA MicroPrep Kit (Zymo Research, R1051) according to the manufacturer's instructions, including on-column DNase I treatment. Libraries were generated from 30-50 ng of RNA using the KAPA RNA HyperPrep Kit with RiboErase (KAPA Biosystems, 08105952001 and 07962274001). Fragmentation and priming were performed at 94°C for 6 min. NEXTflex DNA barcodes were used for adaptor ligation. Libraries were amplified with 14-15 PCR cycles followed by a two steps library amplification cleanup using a 0.8x bead-based cleanup and then an 1x bead cleanup. Library size was determined using the High Sensitivity DNA bioanalyzer kit on a Bioanalyzer 2100 system (Agilent) and concentration was measured using the Qubit 1x dsDNA HS Assay kit (Invitrogen). Libraries were paired-end sequenced (59 bp) on an Illumina NextSeq2000. Sequence reads were processed using the seq2science pipeline (v0.7.1) (van der Sande et al., 2023). In short, paired-end reads were trimmed with fastp (v0.20.1, default settings) and aligned with STAR (v2.7.6a, default settings) to the Homo sapiens genome assembly hg38. Aligned reads were filtered based on a minimum mapping quality of 255. Sample sequencing strandedness was inferred using RSeQC (v4.0.0) and number of reads per gene were measured with HTSeq count (v0.12.4). Low-expressed genes were filtered out prior to differential expression analysis by retaining only those genes with normalized counts $\geq$10 in at least two samples. DESeq2 (v1.44.0) was used for downstream analysis. Log2 FC were shrunk using the lfcShrink function with the "apeglm" method (Zhu et al., 2019). Variance-stabilized expression values were obtained using the vst function in DESeq2 and used as input for heatmap visualization with the pheatmap R package (Kolde, 2025; Love et al., 2014). Gene set enrichment analysis was performed using the ClusterProfiler R package (v4.12.0) (Yu et al., 2012). The gseGO function was used to identify enriched GO terms within the Biological Process ontology. Genes were ranked based on log2 FC. In addition, enrichment was performed using the enrichR R package (v3.2) (Jawaid, 2025), querying the GO_Biological_Process_2023 database. Module scores were calculated based on gene sets defined by differentially expressed genes between wild-type and knockout samples, and applied to single-cell RNA-seq data from human embryos (Zeng et al., 2023) using the AddModuleScore function in Seurat Rpackage (v5.3.1) (Hao et al., 2024).

Immunofluorescence

For iPSCs, cells were grown on vitronectin XF coated coverslips for a maximum of 48 hours. For biotin treatment, cells were incubated with 50 μM biotin for 1 hour prior to fixation. Fixation was performed with 1% paraformaldehyde

(PFA) for 10 min, subsequently washed twice with PBS and permeabilized with 0.5% Triton X-100 in PBS for 10 min. Coverslips were blocked with 0.5% BSA in PBS for 1 hour followed by primary antibody incubation for 1 hour or overnight. After five PBS washes to remove unbound primary antibody, coverslips were incubated with secondary antibody together with Avidin-FITC (1:500) were appropriate for 1 hour. Excess antibody was removed with PBS washes before mounting the coverslips on a microscope slide with Fluoromount G mounting medium with DAPI (Invitrogen, 00-4959-52). For immunostaining of trilineage differentiated cells, cell were fixed in 4% PFA for 10 min, permeabilized with 0.5% Triton X-100 in PBS for 15 min and blocked with 0.5% BSA in PBS-0.1%Tween for 1 hour. Primary antibodies were incubated overnight at 4°C. Cells were washed three times with PBS followed by secondary antibody incubation together with Hoechst in PBS-0.1%Tween for 1 hour at room temperature. Cells were washed three times with PBS and stored in PBS at 4°C till imaging. For immunostaining of somitoids, day 5 human somitoid structures were harvested on ice and washed twice with ice-cold PBS, then fixed in 4% PFA for either 1 hour at room temperature or overnight at 4°C. PFA was then removed and the somitoids were washed three times with PBS supplemented with 1% BSA. Permeabilization and blocking was performed with 2% BSA and 0.5% Triton X-100 in PBS for 2 hours at room temperature. Primary antibodies were diluted in PBS with 2% BSA and 0.3% Triton X-100 and incubated with the somitoids for 3 days at 4°C. The primary antibody solution was then removed and somitoids were washed four times with PBS and 0.2% Triton X-100. Secondary antibodies and DAPI were diluted in PBS with 2% BSA and 0.3% Triton X-100 and incubated overnight at 4°C. The stained somitoids were washed four times with PBS containing 0.2% Triton X-100 and transferred to a 96-well PhenoPlate (Revvity, 6055302) for imaging on a Zeiss LSM900 confocal microscope. The antibodies used were anti-P300 antibody (1:500), anti-OCT3/4 (1:50), anti-SOX2 (1:200), anti-Nestin (1:100), anti-PAX6 (1:222), anti-SOX17 (1:500), anti-CXCR4 (1:500), anti-FOXA2 (1:200) anti-MEOX1 (1:500), Brachyury (1:200) and anti-ZO-1 (1:500). The secondary antibodies used include Alexa Fluor 647-conjugated (1:500), Alexa Fluor 488-conjugated (1:500) and Alexa Fluor 594-conjugated (1:500). Images were acquired using a Zeiss LSM880 or Zeiss LSM900 microscope with ZEN software.

QUANTIFICATION AND STATISTICAL ANALYSIS

MOrgAna analysis

For area, perimeter, and elongation ratio analysis, images of individual somitoids were quantified using the software MOrgAna (version 0.1.1) (Gritti et al., 2021). In brief, a training set consisting of 5-10 images with their corresponding masks was used to train the model with default settings: downscaling of 0.25, edge size of 2, pixel extraction of 0.5, and extraction bias of 0.5. Masks were inspected, and those with incorrect segmentation were manually corrected. The resulting data were exported and analyzed using Python and R. For elongation ratio calculation the following formula was used: 4*pi*area/(perimeter*perimeter), where a value closer to 1 indicates a more rounded morphology.

Quantification of lineage marker expression

For quantification, images were analyzed using CellProfiler software (v4.2.8) (Stirling et al., 2021). The number of cells in each image was quantified by nuclei segmentation based on Hoechst staining, by applying Smooth and then IdentifyPrimaryObject. The mean intensity of SOX17, TBXT, FOXA2 and PAX6 for each segmented object were measured using MeasureObjectIntensity module. Objects with extremely high signals were likely debris, therefore filtered out (SOX17 $\leq$ 0.04, FOXA2 $\leq$ 0.08, TBXT $\leq$ 0.5). For each experimental replicate, 2-3 tiled images were analyzed (n=2-3 technical replicates).

The statistical details of all other experiments can be found in the figure legends, figures, results, and method details.

Stem Cell Reports, Volume *21*

Supplemental Information

From stem cells to somites: Revealing genetic and exogenous factors of human embryogenesis

Maria Teresa Alejo-Vinogradova, Yunna E. Strøm, Lieke A. Lamers, Yolanda W. Chang, Martijn J. Hendriksen, Mariya Moosajee, Michiel Vermeulen, and Suzan Stelloo

Supplemental figures and legends

Figure S1

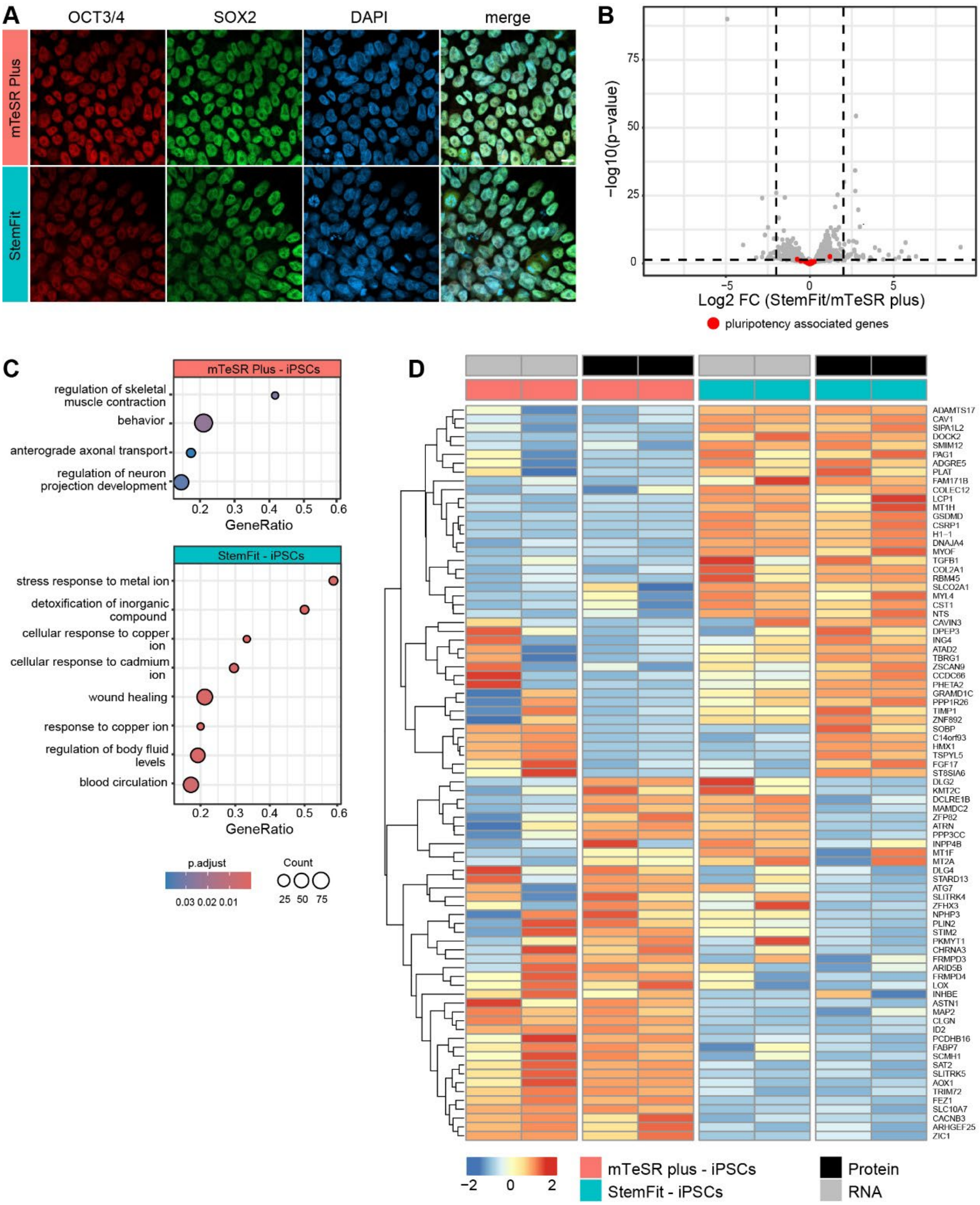

Figure S1. Comparing iPSCs cultured in mTeSR and StemFit media, related to Figure 1.

(A) Immunofluorescence staining of OCT3/4 and SOX2 with DAPI in undifferentiated wild-type EBL.cl3 iPSCs cultured in mTeSR Plus or StemFit medium. Scale bar = 10 µm.

(B) Volcano plot showing differential gene expression between iPSCs cultured in StemFit media versus iPSCs cultured in mTeSR Plus. Red dots represent 28 genes associated with the transcriptional regulation of pluripotent stem cells, based on the Reactome pathway database.

(C) GSEA performed on a ranked list of all genes comparing iPSCs cultured in StemFit versus mTeSR Plus medium. The plot displays the top activated (enriched in StemFit cultured iPSCs) and suppressed (enriched in mTeSR Plus cultured iPSCs) GO Biological Process terms.
(D) Heatmap displaying scaled RNA expression and corresponding protein expression. Only genes or proteins identified as differentially expressed in either modality (RNA-seq or proteomics) with a $\log_2$ FC > 2 and meeting significance thresholds are included.

Figure S2

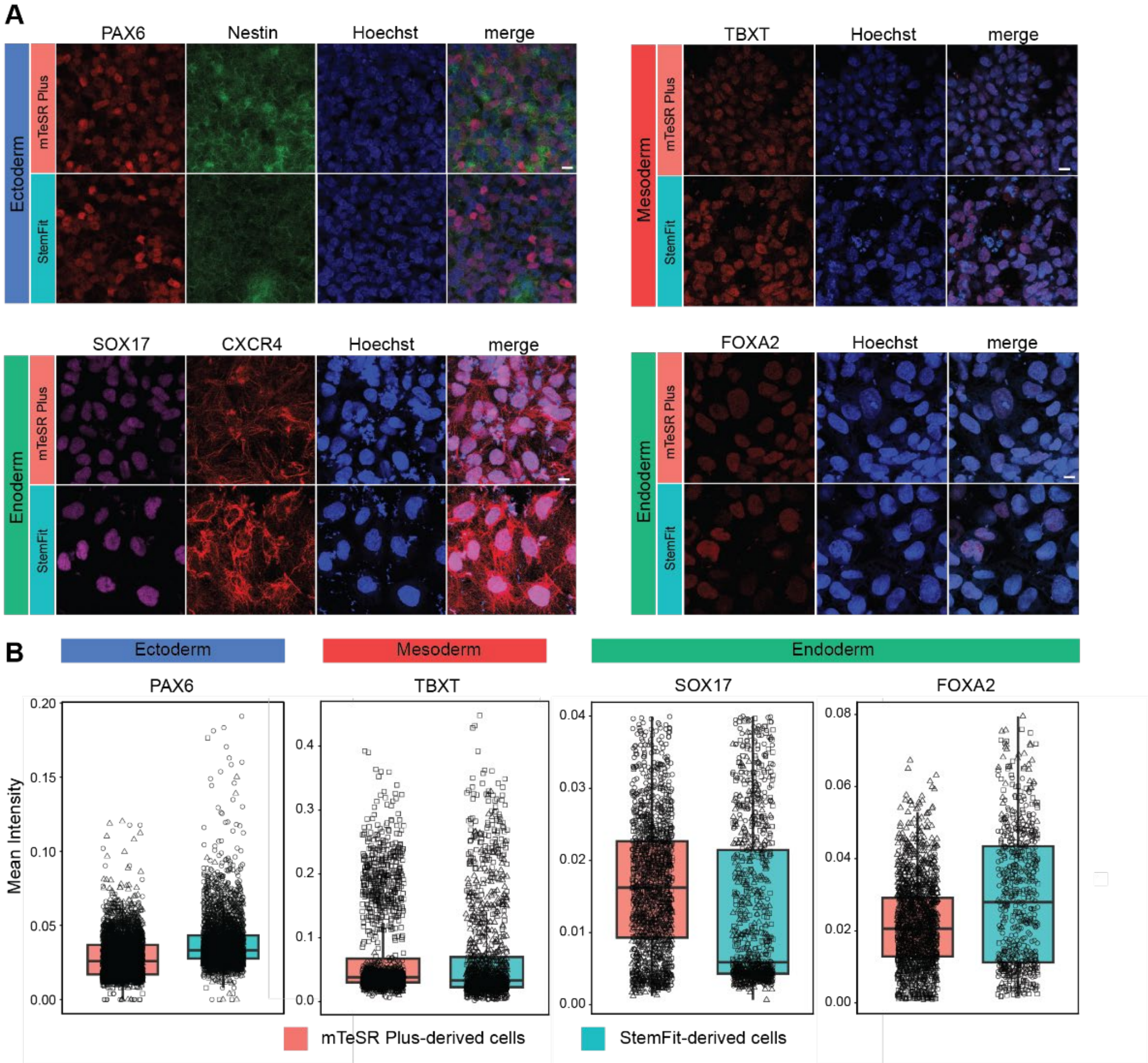

Figure S2. Trilineage differentiation potential of iPSCs maintained in mTeSR plus or StemFit medium, related to Figure 1.

(A) Wild-type EBL.cl3 iPSCs cultured in mTeSR Plus or StemFit medium were subjected to directed differentiation toward ectoderm, mesoderm, and endoderm lineages. Representative immunofluorescence images show expression of lineage-specific markers: ectoderm (PAX6/Nestin), mesoderm (Brachyury/TBXT), and endoderm (SOX17/FOXA2/CXCR4). Nuclei were stained with Hoechst. Scale bar = 10 μm.

(B) Quantification of marker-positive cells is shown as mean fluorescence intensity with each dot representing a single nucleus and different symbols denoting independent experiments.

Figure S3

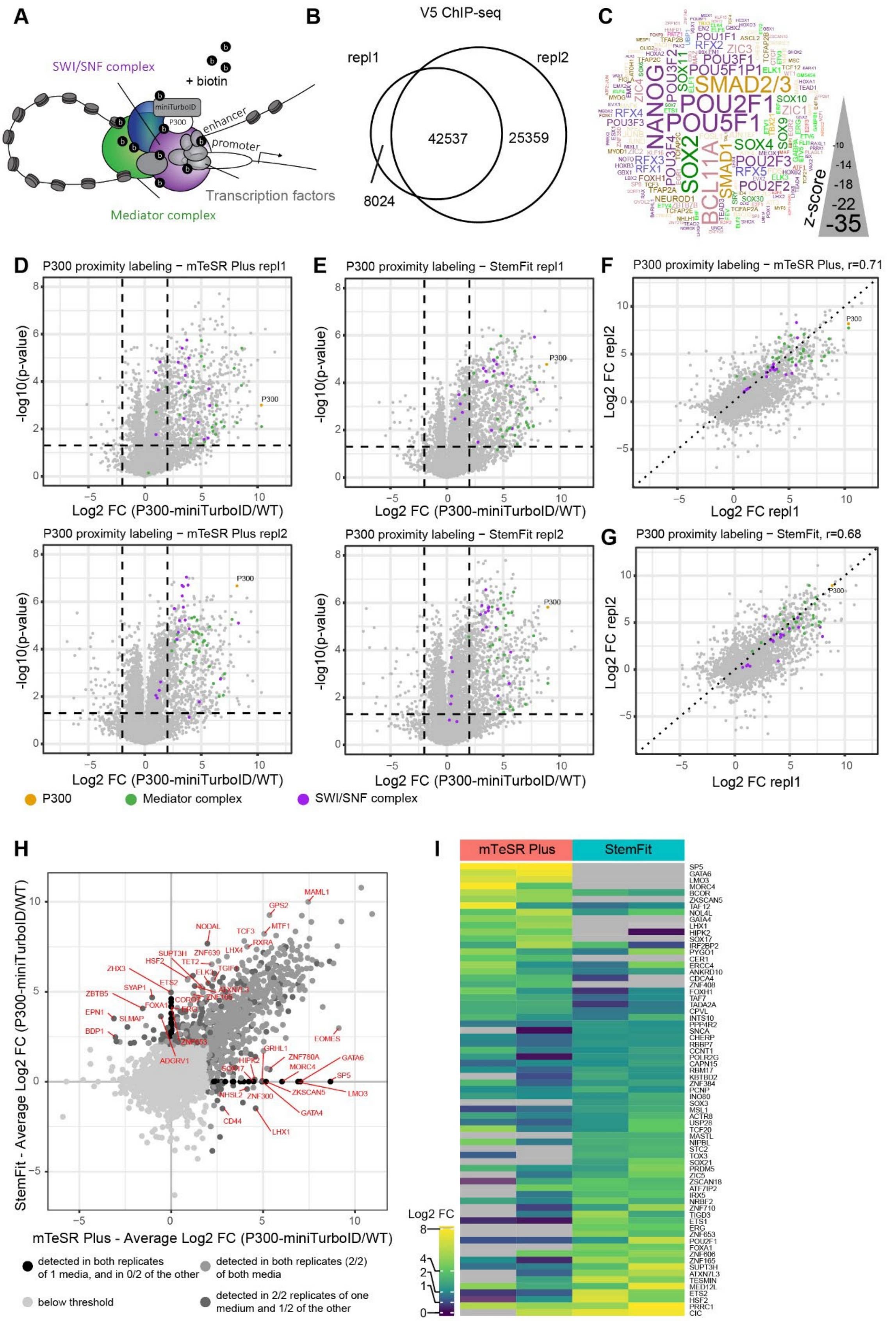
A
SWI/SNF complex
+ biotin
miniTurboID
P300
enhancer
promoter
Transcription factors
Mediator complex
B
V5 ChIP-seq
repl1
repl2
42537
25359
8024
C
z-score
-10
-14
-18
-22
-35
D
P300 proximity labeling − mTeSR Plus repl1
P300 proximity labeling − mTeSR Plus repl2
E
P300 proximity labeling − StemFit repl1
P300 proximity labeling − StemFit repl2
-log10(p-value)
Log2 FC (P300-miniTurboID/WT)
F
P300 proximity labeling − mTeSR Plus, r=0.71
G
P300 proximity labeling − StemFit, r=0.68
Log2 FC repl2
Log2 FC repl1
P300
Mediator complex
SWI/SNF complex
H
StemFit - Average Log2 FC (P300-miniTurboID/WT)
mTeSR Plus - Average Log2 FC (P300-miniTurboID/WT)
detected in both replicates of 1 media, and in 0/2 of the other
detected in both replicates (2/2) of both media
below threshold
detected in 2/2 replicates of one medium and 1/2 of the other
I
mTeSR Plus
StemFit
Log2 FC

Figure S3. P300 proximity labeling reveals culture medium specific transcription factors, related to Figure 1.

(A) Schematic of P300 proximity labeling. Upon biotin stimulation, all proteins in proximity to the fusion protein P300-miniTurboID become biotinylated, including P300 itself through self-biotinylation.

(B) Venn diagram illustrating the overlap of P300 binding sites between replicate 1 and replicate 2.

(C) Transcription factor motif enrichment at P300 binding sites. Font size represents the *Z* score (the smaller the *Z* score, the more enriched, the larger the font size), and colors represent transcription factor families.

(D) Volcano plot of streptavidin affinity purifications of biotin-treated P300-miniTurboID-expressing EBL.cl3 cells compared to biotin-treated wild-type EBL.cl3 iPSCs cultured in mTeSR Plus. Each plot corresponds to an individual biological replicate (with three technical replicates). Horizontal dashed lines represent a *p* value threshold of 0.05 and vertical dashed lines mark a $\log_2$ FC threshold of 2. Members of the SWI/SNF chromatin remodeling complex are highlighted in purple and components of the members of the Mediator complex are shown in green.

(E) Volcano plot of streptavidin affinity purifications of biotin-treated P300-miniTurboID-expressing EBL.cl3 cells compared to biotin-treated wild-type EBL.cl3 iPSCs cultured in StemFit. Each plot corresponds to an individual biological replicate (with three technical replicates). Horizontal dashed lines represent a *p* value threshold of 0.05 and vertical dashed lines mark a $\log_2$ FC threshold of 2. Members of the SWI/SNF chromatin remodeling complex are highlighted in purple and components of the members of the Mediator complex are shown in green.

(F) Correlation plot showing the $\log_2$ FC in protein enrichment from streptavidin affinity purifications of biotin-treated P300-miniTurboID-expressing cells compared to biotin-treated wild-type cells, cultured in mTeSR Plus medium.

(G) Correlation plot showing the $\log_2$ FC in protein enrichment from streptavidin affinity purifications of biotin-treated P300-miniTurboID-expressing cells compared to biotin-treated wild-type cells, cultured in StemFit medium.

(H) Scatter plot showing the mean $\log_2$ FC for proteins enriched in streptavidin affinity purifications of biotin-treated P300-miniTurboID-expressing cells compared to biotin-treated wild-type cells, comparing two different media conditions. $\log_2$ FC in each condition is calculated relative to a biotin-treated wild-type control and averaged across two independent replicates. Only proteins identified in both replicates are included, or retained if identified in both replicates of the other condition. The x-axis indicates the average $\log_2$ FC of iPSCs cultured in mTeSR Plus and the y-axis indicates the average $\log_2$ FC of iPSCs cultured in StemFit. Proteins enriched in either medium are labeled in red.

(I) Heatmap displaying $\log_2$ FC values for proteins enriched in streptavidin affinity purifications of biotin-treated P300-miniTurboID-expressing cells compared to biotin-treated wild-type cells. Proteins included have a $\log_2$ FC > 2 and *p* value <0.05 in both replicates of at least one condition but a $\log_2$ FC <1 in the other condition.

Figure S4

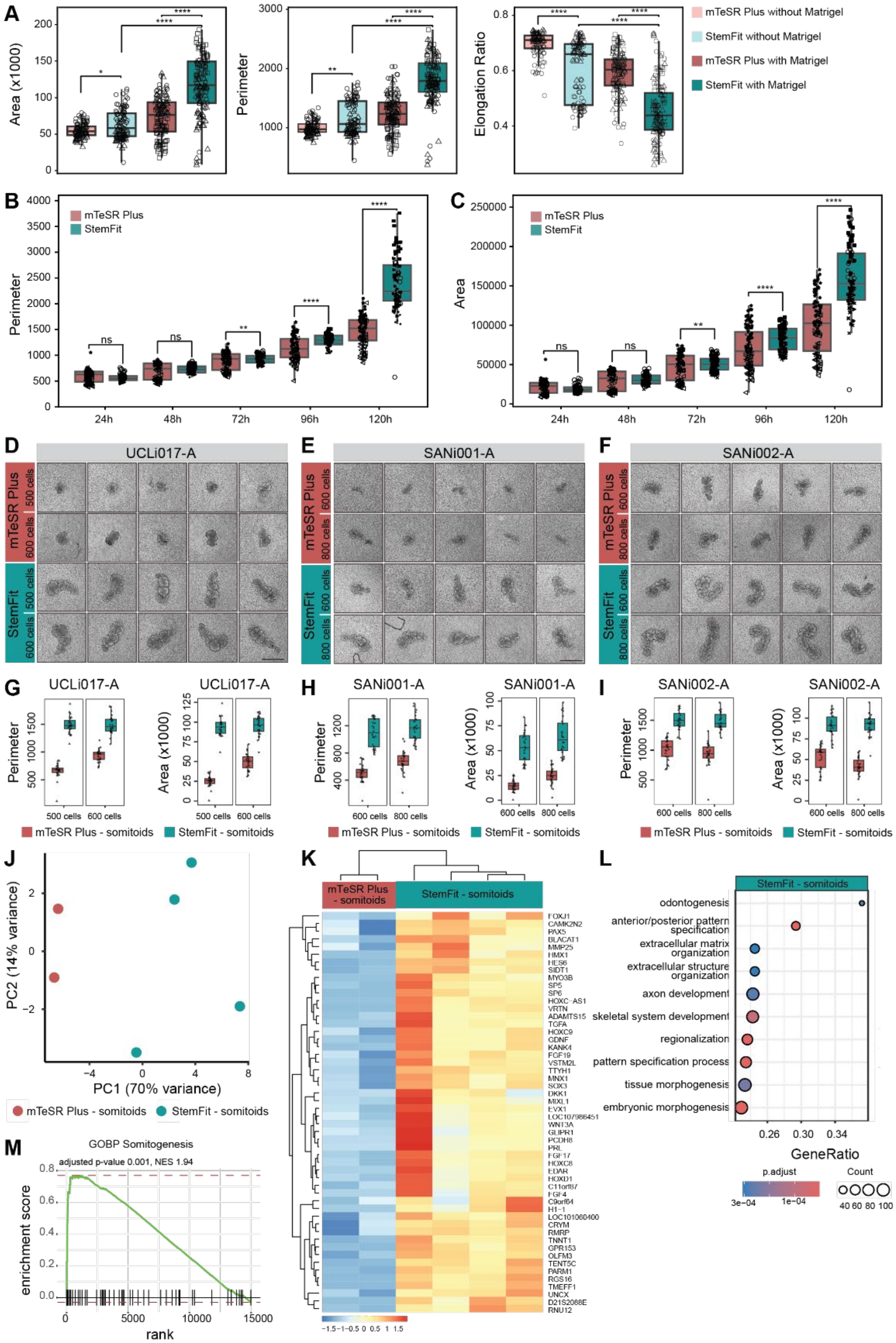

A
Area (x1000)
Perimeter
Elongation Ratio
mTeSR Plus without Matrigel
StemFit without Matrigel
mTeSR Plus with Matrigel
StemFit with Matrigel
B
C
mTeSR Plus
StemFit
Perimeter
Area
24h
48h
72h
96h
120h
D
UCLi017-A
E
SANi001-A
F
SANi002-A
mTeSR Plus
StemFit
500 cells
600 cells
800 cells
G
H
I
mTeSR Plus - somitoids
StemFit - somitoids
J
PC1 (70% variance)
PC2 (14% variance)
K
L
odontogenesis
anterior/posterior pattern specification
extracellular matrix organization
extracellular structure organization
axon development
skeletal system development
regionalization
pattern specification process
tissue morphogenesis
embryonic morphogenesis
GeneRatio
p.adjust
Count
M
GOBP Somitogenesis
adjusted p-value 0.001, NES 1.94
enrichment score
rank

Figure S4. Impact of maintenance medium on somitoid formation efficiency, related to Figure 2.
(A) Quantification of the area, perimeter and elongation ratio of somitoids generated from wild-type EBL.cl3 iPSCs pre-cultured in either mTeSR Plus or StemFit medium with or without Matrigel during the final 24 hours. Each dot represents a single somitoid. Different shapes indicate distinct biological replicates. Reported *p* values are determined with Mann-Whitney-Wilcoxon test two-sided with Bonferroni correction.
(B) Quantification of the perimeter of somitoids across time points generated from wild-type EBL.cl3 iPSCs pre-cultured in either mTeSR Plus or StemFit medium. Somitoids were embedded in Matrigel for the final 24h (120h_mg). Each dot represents a single somitoid. Different shapes indicate distinct biological replicates. Reported *p* values are determined with Mann-Whitney-Wilcoxon test two-sided with Bonferroni correction.
(C) Quantification of the area of somitoids across time points generated from wild-type EBL.cl3 iPSCs pre-cultured in either mTeSR Plus or StemFit medium. Somitoids were embedded in Matrigel for the final 24h (120h_mg). Each dot represents a single somitoid. Different shapes indicate distinct biological replicates. Reported *p* values are determined with Mann-Whitney-Wilcoxon test two-sided with Bonferroni correction.
(D) Representative images of day 5 Matrigel-embedded somitoids derived from UCLi017-A iPSCs pre-cultured in mTeSR Plus or StemFit medium. Differentiation was started with the indicated amount of cells. Scale bar = 200 µm.
(E) Representative images of day 5 somitoids derived from SANi001-A iPSCs pre-cultured in mTeSR Plus or StemFit medium. Differentiation was started with the indicated amount of cells. Scale bar = 200 µm.
(F) Representative images of day 5 somitoids derived from SANi002-A iPSCs pre-cultured in mTeSR Plus or StemFit medium. Differentiation was started with the indicated amount of cells. Scale bar = 200 µm.
(G) Quantification of the perimeter and area of somitoids generated from UCLi017-A iPSCs pre-cultured in either mTeSR Plus or StemFit medium. Each dot represents a single somitoid at day 5.
(H) Quantification of the perimeter and area of somitoids generated from SANi001-A iPSCs pre-cultured in either mTeSR Plus or StemFit medium. Each dot represents a single somitoid at day 5.
(I) Quantification of the perimeter and area of somitoids generated from SANi002-A iPSCs pre-cultured in either mTeSR Plus or StemFit medium. Each dot represents a single somitoid at day 5.
(J) PCA based on the top 500 most variable expressed genes in somitoids derived from iPSCs pre-cultured in either mTeSR Plus or StemFit medium. Each dot represents an independent biological replicate.
(K) Heatmap showing scaled expression of 52 differentially expressed genes (p value <0.05 and log2 FC >2) between somitoids derived from iPSCs maintained in different pre-differentiation media (mTeSR Plus vs. StemFit).
(L) GSEA performed on a ranked list of all genes comparing somitoids derived from iPSCs cultured in StemFit versus mTeSR Plus medium. The plot displays the top activated (enriched in StemFit cultured iPSCs) and suppressed (enriched in mTeSR Plus cultured iPSCs) GO Biological Process terms.
(M) Enrichment plot for the gene set “GOBP Somitogenesis” from the MSigDB database. The vertical black lines indicate the positions of genes from the set (67 genes) within the ranked list. Genes are ranked from most upregulated in somitoids derived from StemFit cultured iPSCs compared to somitoids derived from mTeSR Plus cultured iPSCs on the left to most downregulated on the right.

Figure S5

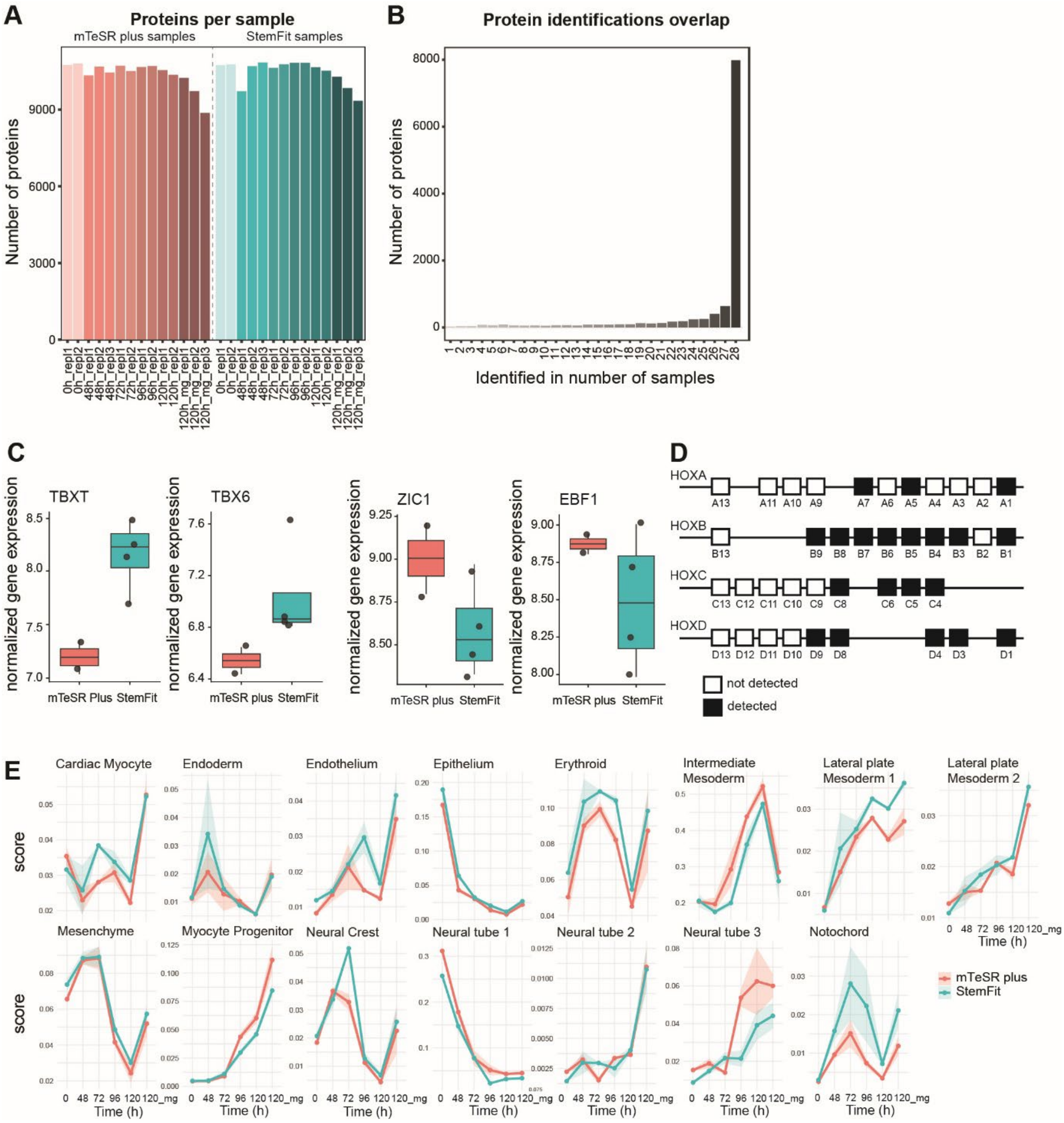

Figure S5. Temporal proteomic analysis of somitoid differentiation, related to Figure 3.

(A) Number of proteins retained after removal of contaminants and retaining proteins identified with at least two peptides.

(B) Distribution of protein overlap across the 28 samples.

(C) RNA expression levels corresponding to four selected differentially expressed proteins (DEPs).

(D) HOX protein detection across clusters HOXA–D. Black boxes indicate detection.

(E) Scaden-based deconvolution of proteomics data showing relative cell type contributions. Values represent relative abundances and are intended for comparison across time points rather than absolute quantification.

Figure S6

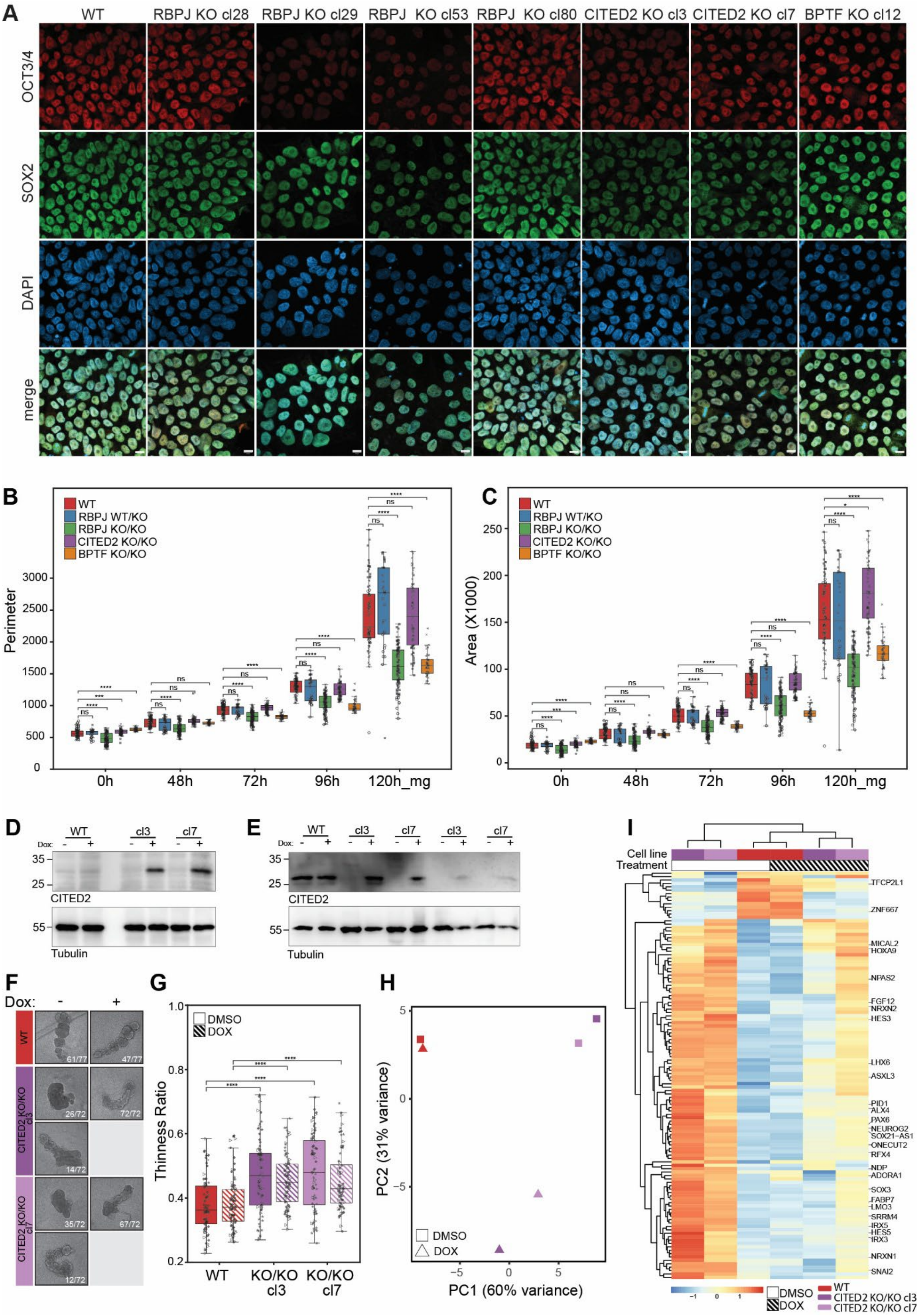
A
WT
RBPJ KO cl28
RBPJ KO cl29
RBPJ KO cl53
RBPJ KO cl80
CITED2 KO cl3
CITED2 KO cl7
BPTF KO cl12
OCT3/4
SOX2
DAPI
merge
B
WT
RBPJ WT/KO
RBPJ KO/KO
CITED2 KO/KO
BPTF KO/KO
Perimeter
0h
48h
72h
96h
120h_mg
C
Area (X1000)
D
E
CITED2
Tubulin
Dox:
F
G
DMSO
DOX
Thinness Ratio
WT
KO/KO cl3
KO/KO cl7
H
PC2 (31% variance)
PC1 (60% variance)
I
Cell line
Treatment
TFCP2L1
ZNF667
MICAL2
HOXA9
NPAS2
FGF12
NRXN2
HES3
LHX6
ASXL3
PID1
ALX4
PAX6
NEUROG2
SOX21-AS1
ONECUT2
RFX4
NDP
ADORA1
SOX3
FABP7
LMO3
SRRM4
IRX5
HES5
IRX3
NRXN1
SNAI2
CITED2 KO/KO cl3
CITED2 KO/KO cl7

Figure S6. Pluripotency and morphological characterization of BPTF, RBPJ, and CITED2 knockout iPSCs and somitoids, related to Figure 5.

(A) Immunofluorescence staining of OCT3/4 and SOX2 with DAPI in undifferentiated wild-type EBL.cl3 iPSCs and BPTF, RBPJ, and CITED2 knockout iPSCs. Scale bar = 10 μm.

(B) Quantification of the perimeter of somitoids across time points generated from wild-type EBL.cl3 iPSCs and RBPJ, CITED2, and BPTF knockout iPSCs. Somitoids were embedded in Matrigel for the final 24h (120h_mg). Each dot represents a single somitoid. Different shapes indicate distinct biological replicates. Reported *p* values are determined with Mann-Whitney-Wilcoxon test two-sided with Bonferroni correction.

(C) Quantification of the area of somitoids across time points generated from wild-type EBL.cl3 iPSCs and RBPJ, CITED2, and BPTF knockout iPSCs. Somitoids were embedded in Matrigel for the final 24h (120h_mg). Each dot represents a single somitoid. Different shapes indicate distinct biological replicates. Reported *p* values are determined with Mann-Whitney-Wilcoxon test two-sided with Bonferroni correction.

(D) Western blot of protein extracts from wild-type EBL.cl3 iPSCs and CITED2 knockout clones with a doxycycline inducible expression for CITED2 integrated. Cells were untreated (DMSO) or treated with doxycycline for 24 hours before collection. Blots were incubated with anti-CITED2 and anti-Tubulin antibodies.

(E) Western blot of protein extracts from Matrigel-embedded somitoids derived from wild-type EBL.cl3 iPSCs and CITED2 knockout clones with a doxycycline inducible expression for CITED2 integrated. Cells were untreated (DMSO) or treated with doxycycline over the course of differentiation. Blots were incubated with anti-CITED2 and anti-Tubulin antibodies.

(F) Representative images of Matrigel-embedded somitoids derived from wild-type EBL.cl3 iPSCs and CITED2 knockout clones with a doxycycline inducible expression for CITED2 integrated. The notation *x/x* indicates the number of somitoids displaying the representative phenotype out of the total number analyzed. If the total is not accounted for, remaining samples exhibited mixed or wild-type-like phenotypes.

(G) Quantification of the elongation ratio of Matrigel-embedded somitoids generated from wild-type EBL.cl3 iPSCs and CITED2 knockout iPSCs with a doxycycline inducible expression for CITED2 integrated. Cells were untreated (DMSO) or treated with doxycycline over the course of differentiation. Each dot represents a single somitoid. Different shapes indicate distinct biological replicates, comprising both knockout clones. Reported *p* values are determined with Mann-Whitney-Wilcoxon test two-sided with Bonferroni correction.

(H) PCA based on the most variable expressed genes in somitoids generated from wild-type EBL.cl3 iPSCs and CITED2 knockout iPSCs with a doxycycline inducible expression for CITED2 integrated. Cells were untreated (DMSO; squared) or treated with doxycycline (triangles) over the course of differentiation.

(I) Heatmap showing scaled expression of differentially expressed genes (p value <0.05 and $\log_2$ FC >2) between Matrigel-embedded somitoids derived from wild-type cells and CITED2 knockout iPSCs, untreated (DMSO) or treated with doxycycline.

Figure S7

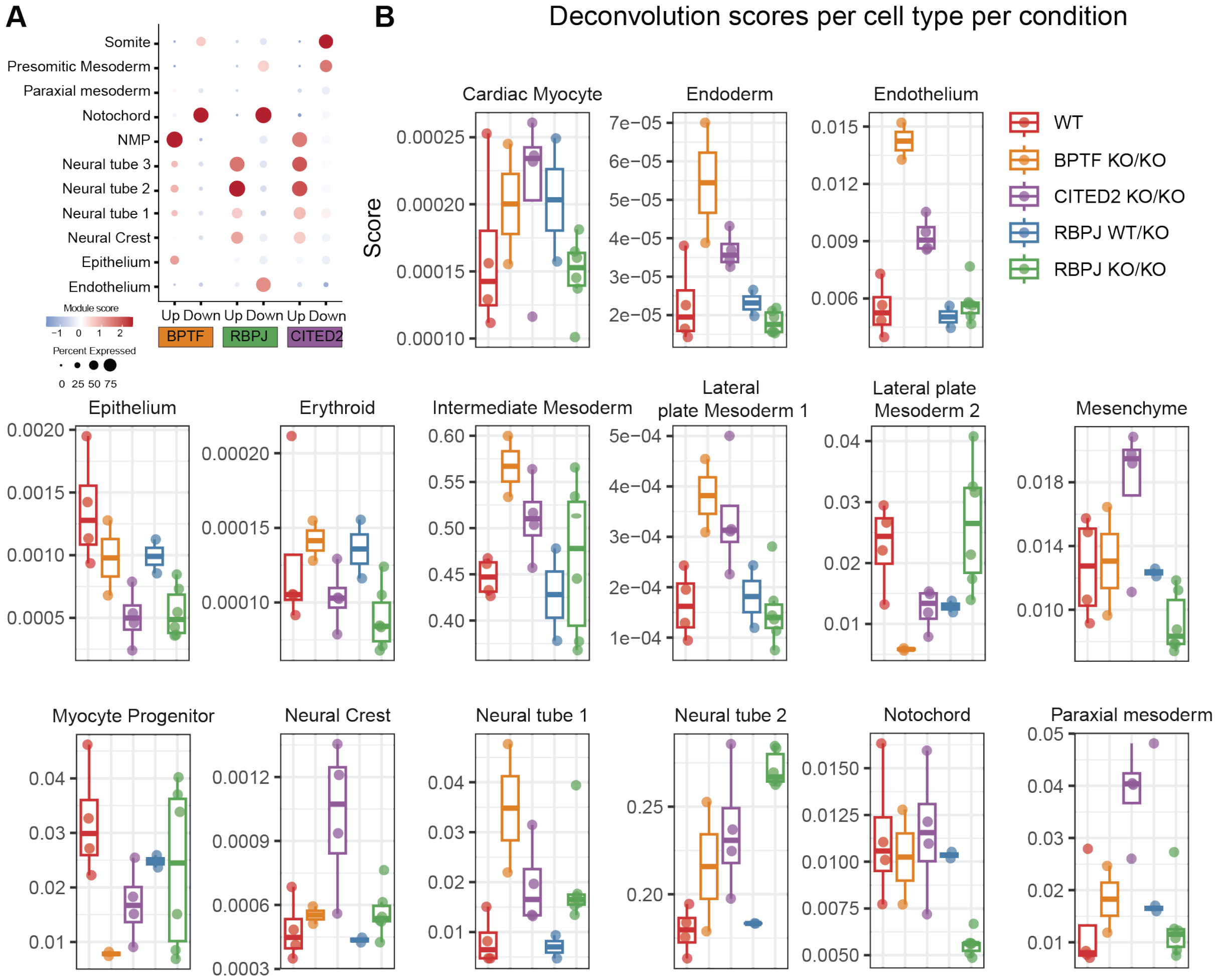

Figure S7. Targeted knockout of BPTF, RBPJ and CITED2 affects gene expression in somitoids, related to Figure 5.

(A) Dot plot of AddModuleScore enrichment across cell types in scRNA-seq of human embryos at gastrulation stage (GSE155121). Module scores were computed from gene sets defined by the differentially expressed genes, separated into up- and downregulated subsets for each knockout. Dot color indicates module score, and dot size reflects the fraction of expressing cells. Only enriched cell types are shown.

(B) Boxplot of cell type predictions inferred by Scaden in knockout samples, using human embryo scRNA-seq as reference. Distributions reflect relative cell type abundances and enable comparison between conditions rather than absolute quantification.

Figure S8

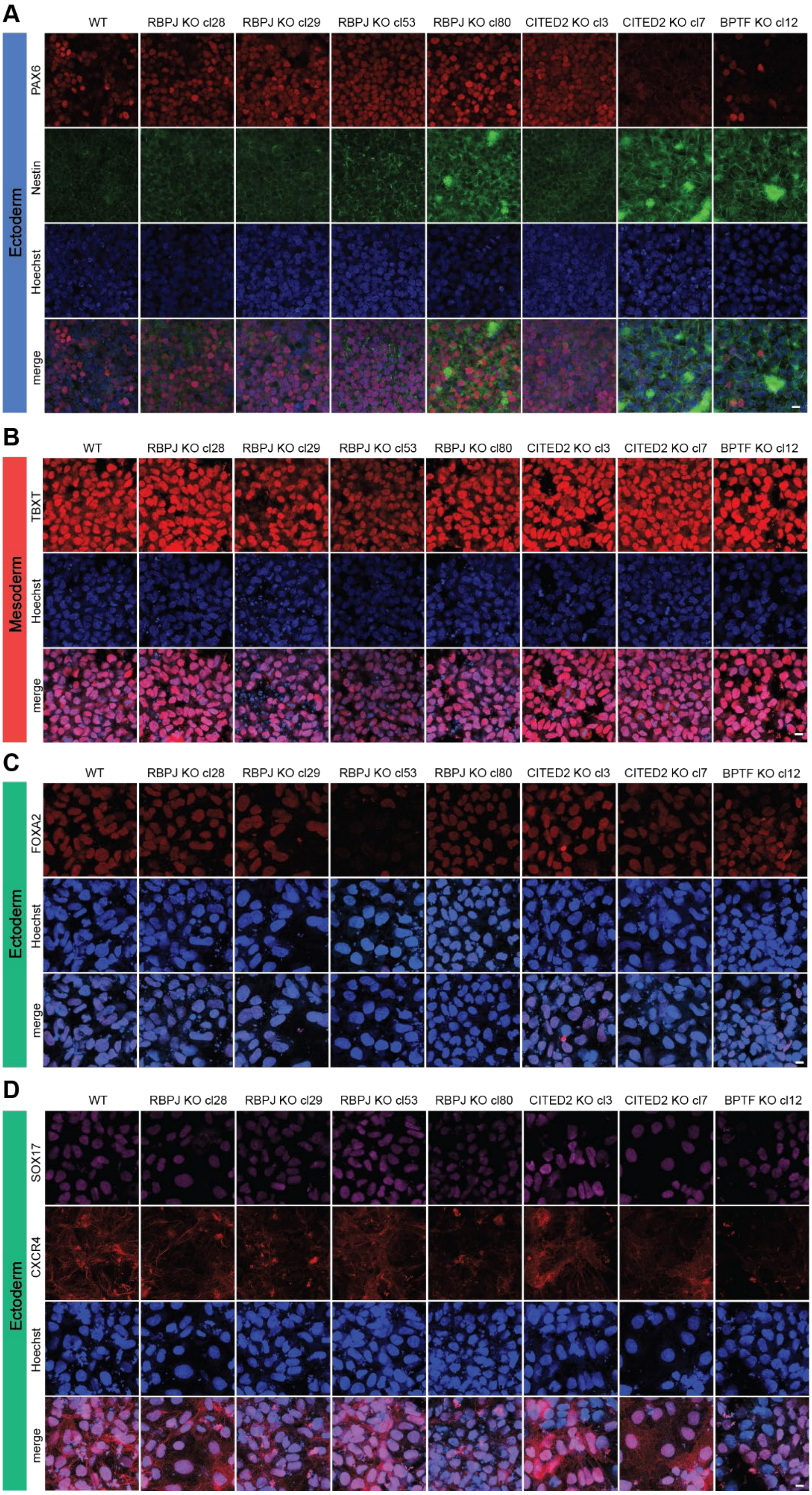
A
WT
RBPJ KO cl28
RBPJ KO cl29
RBPJ KO cl53
RBPJ KO cl80
CITED2 KO cl3
CITED2 KO cl7
BPTF KO cl12
Ectoderm
PAX6
Nestin
Hoechst
merge
B
Mesoderm
TBXT
Hoechst
merge
C
Ectoderm
FOXA2
Hoechst
merge
D
Ectoderm
SOX17
CXCR4
Hoechst
merge

Figure S8. Trilineage differentiation potential of BPTF, CITED2 and RBPJ knockout iPSCs, related to Figure 5.
wild-type EBL.cl3 iPSCs (WT) and BPTF, CITED2 and RBPJ knockout iPSCs cultured in mTeSR Plus medium were subjected to directed differentiation toward ectoderm, mesoderm, and endoderm lineages. Representative immunofluorescence images show expression of lineage-specific markers: (A) ectoderm (PAX6/Nestin), (B) mesoderm (Brachyury/TBXT), and (C and D) endoderm (SOX17/FOXA2/CXCR4). Nuclei were stained with Hoechst. Scale bar = 10 μm.

Figure S9

A

	Wild-type iPSCs
D8S1179	13,15
D21S11	28,31.2
D7S820	8,10
CSF1PO	11,12
D3S1358	16,18
TH01	7,9.3
D13S317	13,13
D16S539	13,14
D2S1338	18,24
D19S433	14,14
vWA	14,18
TPOX	8,8
D18S51	13,15
AMEL	X,X
D5S818	11,12
FGA	22,24

B

	SANi001-A	SANi002-A	UCLi017-A
D8S1179	13,14	12,14	11,14
D21S11	28,29	30,30	28,30
D7S820	10,11	9,12	10,11 **
CSF1PO	9,11	10,11	-*
D3S1358	17,18	14,15	15,17
TH01	8,9.3	7,7	7,9.3
D13S317	12,12	12,13	12,12
D16S539	11,12	11,12	12,13
D2S1338	19,20	19,23	-*
D19S433	13,15	15,15	14,15**
vWA	16,17	16,17	16,19
TPOX	8,8	8,11	8,8
D18S51	13,15	12,16	-*
AMEL	X,Y	X,X	X,Y
D5S818	11,12	11,12	11,11
FGA	22,23	23,23	-*

C

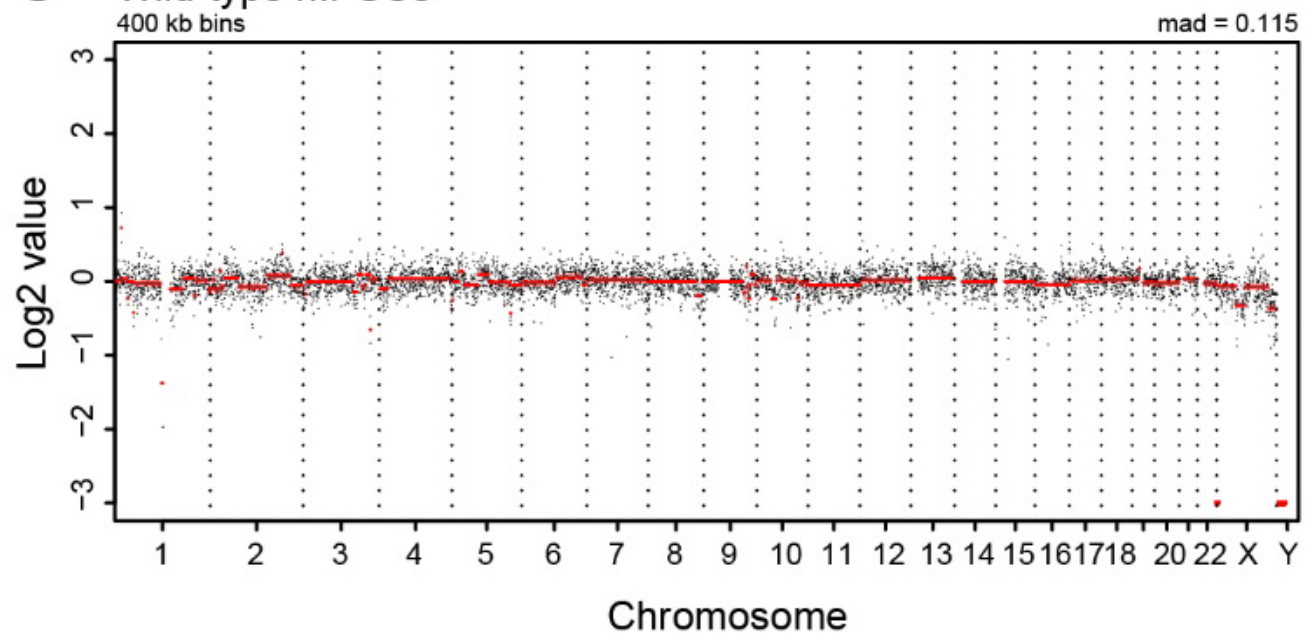

D

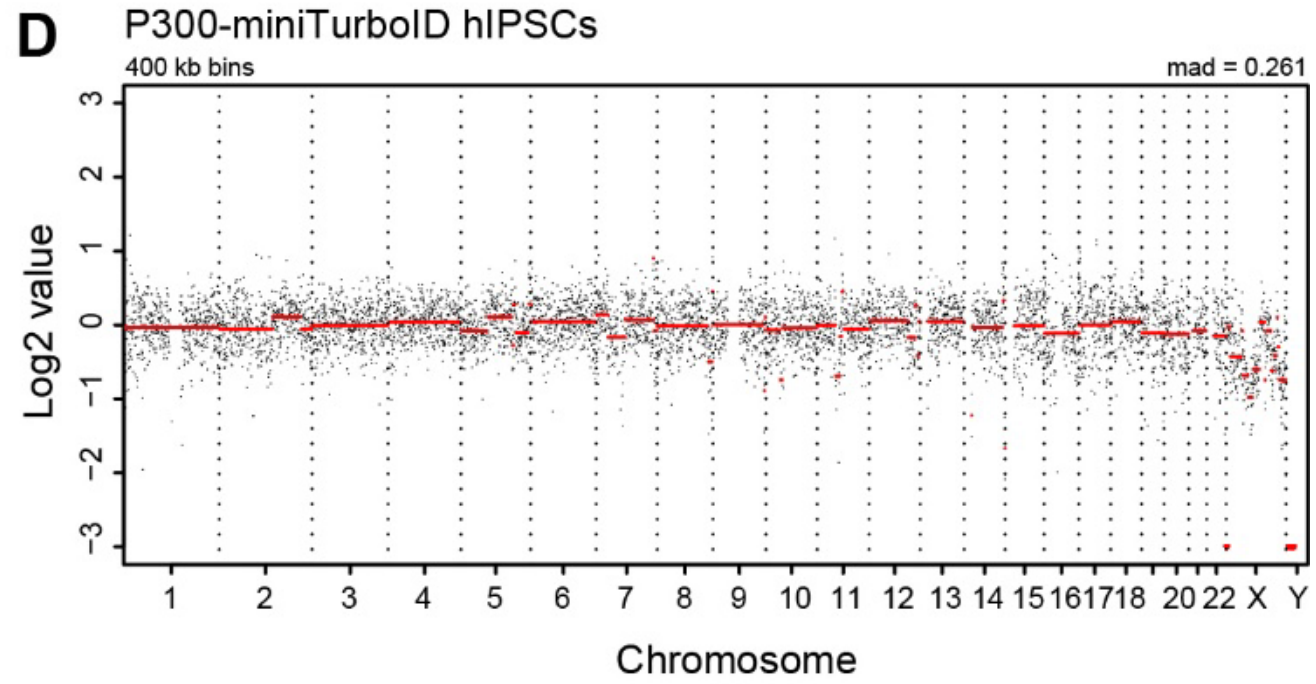

Figure S9. STR and copy number profile validation in iPSCs, related to Methods.
(A) STR analysis of the wild-type EBL.cl3 iPSCs used in the main figures of this study.
(B) STR analysis of SANi001-A, SANi001-2-A, and UCLi017-A iPSCs. ** weak signal, * no signal.
(C) Copy number profile of wild-type EBL.cl3 iPSCs.
(D) Copy number profile of EBL.cl3 iPSCs expressing p300-V5-miniTurboID-T2A-puro.

Supplemental tables

Table S8. Primer sequences, related to Methods.

**Name**	**Sequence 5'-3'**
169_RBPJ_outside_F	TTCTTTTGCTCCCTCCCCAC
170_RBPJ_outside_R	ACATTGGGATATGGCAGGTACC
171_RBPJ_inside_R	AAAAGGCTCCCCACTGCTG
175_BPTF_outside_F	ATCCCGCCCCTTGAATTTCC
176_BPTF_outside_R	GCTCTGCTACATCAATCACGAAG
177_BPTF_inside_F	TGGGGATTTGCTTTGCTGTG
190_CITED2_outside_F	TTCACGCCGAAGAAGTTGG
191_CITED2_outside_R	GCCCTTTTCACTTCCAGGAC
